# *Staphylococcus aureus* adapts to the host nutritional environment by coordinating the activity of central metabolic enzymes

**DOI:** 10.1371/journal.ppat.1014183

**Published:** 2026-05-04

**Authors:** Reginald A. Woods, Iván C. Acosta, Zachary J. Resko, Charles Agbavor, Luka Svet, Manaar Yousef, Wei Ping Teoh, Victor J. Torres, Laty A. Cahoon, Francis Alonzo

**Affiliations:** 1 Department of Pharmaceutical Sciences, University of Illinois at Chicago – College of Pharmacy, Chicago, Illinois, United States of America; 2 Department of Microbiology and Immunology, University of Illinois at Chicago – College of Medicine, Chicago, Illinois, United States of America; 3 Department of Microbiology and Immunology, Loyola University Chicago - Stritch School of Medicine, Maywood, Illinois, United States of America; 4 Department of Biological Sciences, University of Pittsburgh, Pittsburgh, Pennsylvania, United States of America; 5 Department of Host-Microbe Interactions, St Jude Children’s Research Hospital, Memphis, Tennessee, United States of America; 6 Lead contact; Trinity College Dublin, IRELAND

## Abstract

The nutritional demands imposed by disparate infection sites represent a significant barrier to bacterial survival. Yet, the proclivity of pathogens such as *Staphylococcus aureus* to cause disease at nearly all host sites implies significant metabolic flexibility to promote infection. *S. aureus* catabolizes glucose in several ways, including the phosphotransacetylase (Pta) – acetate kinase (AckA) pathway. The Pta-AckA pathway uses acetyl-CoA derived from the major glycolytic end-product, pyruvate, to rapidly generate ATP, producing acetate as a byproduct. Yet, flux through Pta-AckA necessitates coordinating glycolytic activity with the production of acetyl-CoA and its delivery to Pta-AckA. The generation of acetyl-CoA occurs through the pyruvate dehydrogenase (PDH) complex, an enzyme that requires attachment of the metabolic cofactor, lipoic acid, for its function. Thus, delivery of lipoic acid to enzyme complexes has the potential to serve as a determinant of metabolic adaptation. Here, we provide evidence for a functional link between the lipoic acid transfer enzyme, LipL, and Pta. We demonstrate *pta* and *lipL* are transcriptionally coupled and that Pta and LipL directly interact, with potential to direct metabolic flux through Pta-AckA. Both Δ*pta* and Δ*lipL* mutants are defective for acetate production with evidence for alternative fates for pyruvate that depend on blockade upstream or downstream of the pyruvate node. The functional pairing of Pta and LipL is required for optimal skin infection, whereas Pta and LipL have separable functions in bloodstream infection. Furthermore, we found that a complete block to acetate production leads to significant attenuation *in vivo*, cementing a direct role for acetogenesis in infection. Together, our results establish a mechanism by which *S. aureus* regulates metabolite flux by coupling enzymes in linked metabolic pathways to promote energy balance and survival during infection.

## Author summary

*Staphylococcus aureus* causes infections within nearly all host tissues. Several virulence traits contribute to its survival at diverse sites, including the ability to produce myriad toxins, adhesins, and immunomodulatory enzymes. *S. aureus* also has remarkable metabolic plasticity, allowing it to overcome host-imposed nutrient restriction. In this work, we establish a genetic and functional coupling that links two metabolic pathways involved in *S. aureus* central carbon metabolism. Our work suggests that the coordinated expression and activity of an enzyme required for the delivery of the metabolic cofactor, lipoic acid, and an enzyme involved in acetogenesis, facilitates optimal metabolite flux and promotes *S. aureus* survival during infection. Furthermore, our studies support a direct role for acetate production in *S. aureus* survival during infection. Altogether, this work introduces a new facet of regulatory control over metabolic plasticity in *S. aureus* with important implications for pathogenesis.

## Introduction

The host nutritional landscape can dramatically affect the ability of bacterial pathogens to establish infection [1–6]. The adaptation of microbes to host nutrient deprivation is crucial for pathogens that cause infection in a wide range of host tissues [7]. *Staphylococcus aureus* is a Gram-positive pathogen that can infect nearly all host tissues and causes significant morbidity and mortality globally [8,9]. To successfully establish infection, this bacterium not only uses tissue-specific defenses that block immune clearance, but also harnesses the host environment to optimize metabolism and energy expenditures [3,4,6,10,11]. *S. aureus* adapts to the host nutritional milieu by employing a complex network of transcriptional regulators that fine-tune metabolic gene expression to promote survival and growth [4,12]. The ability of *S. aureus* to optimize metabolic efficiency during infection extends beyond transcriptional regulation. Several bioinformatic and functional studies indicate that the directionality and efficiency of metabolite production are enhanced by high-affinity or transient protein-protein interactions and post-translational modifications [13–16], highlighting critical post-translational checkpoints in metabolic adaptation. As a pathogen that evolved closely with humans, *S. aureus* also adapts by engaging in piracy of host nutrients during infection [1,2,5,17,18]. Indeed, *S. aureus* can use host lipids, carbohydrates, metals, and proteins to augment metabolite flux or bypass energetically demanding biological processes [2–4,19–21]. Work from our lab and others suggests that the ability of *S. aureus* to establish infection in diverse tissues is due in part to adaptations that permit scavenging of key nutrients or redirecting of metabolism to promote survival [1,2,5,17,18,22].

One molecule with low bioavailability in the host is the metabolic cofactor lipoic acid, on account of its synthesis as a protein-bound precursor and its rapid clearance after dietary consumption [23–25]. Lipoic acid is an organosulfur derivative of the medium-chain fatty acid, octanoic acid, that is required for the activation of α-ketoacid dehydrogenase complexes. Its redox-sensitive dithiolane ring aids in shuttling substrates through each complex [25]. The lipoic acid-dependent enzymes of *S. aureus* include pyruvate dehydrogenase (PDH), 2-oxoglutarate dehydrogenase (OGDH), and branched-chain 2-oxoacid dehydrogenase (BCODH) complexes, as well as the glycine cleavage system (GCS) [1,26]. *S. aureus* synthesizes lipoic acid *de novo* and scavenges it from host environments to promote survival [1]. The use of both pathways during infection is exemplified by evidence that shows lipoic acid scavenging is required for survival in some host tissues (kidneys), but not others (heart) [1]. Central to lipoic acid acquisition is the amidotransferase, LipL, which harnesses lipoic acid bound to the H subunits of the glycine cleavage system (GcvH) or the GcvH homolog, GcvH-L, for delivery to conserved lysine residues on the E2 subunits of each α-ketoacid dehydrogenase complex (E2-PDH, E2-OGDH, and E2-BCODH) [1,26]. Lipoic acid attachment to these enzyme complexes represents an essential post-translational modification, as LipL-deficient strains of *S. aureus* have compromised growth *in vitro* and are highly attenuated during bloodstream infection [5,26]. Altogether, these studies suggest that dynamic delivery of lipoic acid to metabolic enzyme complexes is likely required to facilitate metabolic plasticity and pathway adaptations needed to promote bacterial survival during infection.

LipL-dependent lipoylation of the E2 subunits of PDH and OGDH is critical for glycolysis and the tricarboxylic acid (TCA) cycle, respectively [26]. PDH generates acetyl-CoA from pyruvate upon exit from glycolysis, and OGDH generates succinyl-CoA from α-ketoglutarate in the TCA cycle [1,26]. The acetyl-CoA generated by PDH has several fates depending on the status of the cell and the nutrients available for use. These include the potential to enter fermentative pathways, fatty acid biosynthesis, TCA cycle, and nucleotide biosynthesis pathways [27–29]. During carbon overflow *in vitro, S. aureus* primarily ferments glucose by shuttling acetyl-CoA into the phosphotransacetylase (Pta) - acetate kinase (AckA) pathway to rapidly synthesize ATP [28]. Pta catalyzes the generation of acetyl-phosphate from acetyl-CoA, which is used in a substrate-level phosphorylation reaction by AckA to generate acetate and ATP [27,28]. The Pta-AckA pathway promotes rapid growth *in vitro* during overflow metabolism when glucose and oxygen are abundant [27]. Extensive prior work established that the Pta-AckA pathway is vital for energy production and metabolic homeostasis during overflow metabolism in *S. aureus* [28,30]. Though *pta* is commonly positioned near, or co-transcribed with, *ackA* in many bacterial pathogens, we previously determined that in *S. aureus*, the start codon of *lipL* is located two nucleotides downstream of the *pta* stop codon [26]. The potential genetic linkage between *pta* and *lipL* and the dependency on efficient LipL-dependent lipoyl transfer for PDH activity highlights the relevance of LipL to central metabolism and suggests a potential functional link to acetate fermentation pathways.

As a facultative anaerobe, *S. aureus* can replicate in both high and low oxygen environments [3,31]. Past studies indicate that Pta-AckA activity increases during growth in low oxygen to support NAD+ generation and fermentative processes [27,32]. This extends the role of Pta beyond overflow metabolism and implies relevance of the pathway to conditions that occur during host infection (e.g., low oxygen). Inactivation of the Pta-AckA pathway shifts *S. aureus* metabolism away from acetate fermentation, leading to increased carbon flux through glycolysis and the TCA cycle [28]. Pathway inactivation also leads to early bacterial cell death in some *S. aureus* strains due to the induction of the *cid* genes [28]. This is thought to be due to the imposed metabolic block at pyruvate and the cellular requirement to combat intracellular pyruvate accumulation [28]. The *cid* locus comprises three genes, *cidABC*, which play a vital role in stationary phase cell death, autolysis, and biofilm formation [33–36]. CidA is a functional holin that supports endolysin-induced cell lysis, and CidC is a pyruvate:menaquinone oxidoreductase that converts pyruvate directly to acetate [37,38]. Although its mechanism of action is not fully understood, CidB is thought to act in tandem with CidA to regulate cell lysis and is necessary for full activation of CidC [39]. Work from Thomas *et al.* found that inactivation of CidC decreased the rate of stationary phase cell death, which disrupts *S. aureus* biofilm development and biofilm-related disease outcomes [36]. Despite the connection between Pta-AckA and CidC as compensatory pathways to accommodate carbon flow, we do not yet have a complete picture of how Pta, CidC, and LipL interface to promote metabolic adaptability during infection. To date, no studies have assessed the individual versus combined contributions of Pta and CidC during *S. aureus* infection with planktonic cells.

The prevailing evidence thus far suggests there are contributions from several metabolic pathways, including glycolysis, as well as lipoic acid biosynthesis and salvage, to *S. aureus* adaptation to host tissues [1,40–42]. The unusual positioning of *pta* and *lipL* genes in the *S. aureus* genome may further link lipoic acid transfer and acetogenesis in ways that dictate the fate of major glycolytic metabolites. In this work, we investigated the convergence of lipoic acid relay and Pta-AckA pathways and determined the relative contribution of each to pathogenesis. We show that *pta* and *lipL* share a distinct genetic organization in *Staphylococci* that diverges from pathogenic firmicutes and Gram-negative pathogens. *pta* and *lipL* are co-transcribed, and the two encoded proteins (Pta and LipL) directly interact. This genetic and functional linkage promotes infection in the skin, whereas separable functions for Pta and LipL are required for systemic infection. Furthermore, we verified the established block at the pyruvate node in the absence of Pta and determined that a similar block occurs in the absence of LipL, leading to a commensurate increase in *cidC* transcripts and lactate production during aerobic growth. Finally, we found that bacterial acetate production is required for survival in both skin and systemic infection. Altogether, these studies provide new insight into the factors governing the metabolic plasticity of *S. aureus* during infection with potential applicability to other pathogens.

## Results

### *pta* and *lipL* are genetically linked in *Staphylococci*

**Fig 1:**
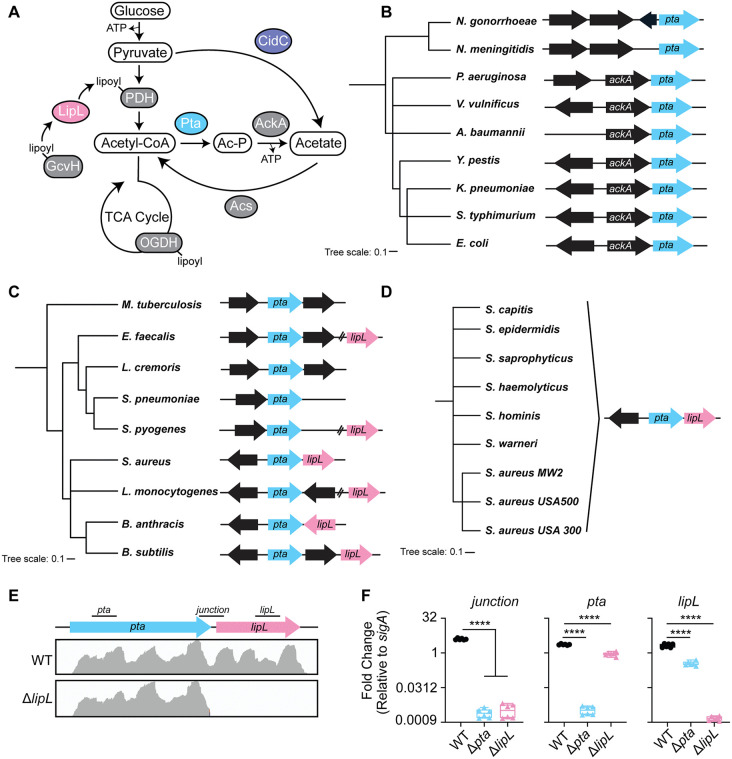
*pta* and *lipL* are genetically linked in *Staphylococci.* **(A)** Model of acetate fermentation in *S. aureus*. Glucose is converted to pyruvate, the primary end-product of glycolysis. Pyruvate has several fates, one of which is its oxidative decarboxylation by pyruvate dehydrogenase (PDH) to generate Acetyl-CoA. Pyruvate can also be directly decarboxylated (CidC) to produce acetate. Acetyl-CoA can enter the tricarboxylic acid (TCA) cycle or is used by the phosphotransacetylase (Pta) to generate Acetyl-phosphate (Ac-P), the substrate for acetate kinase (AckA). The activity of AckA generates Acetate and ATP. PDH activity is regulated by lipoic acid (lipoyl) attachment, which is mediated by delivery of lipoic acid from the H subunit of the glycine cleavage system (GcvH) by the amidotransferase, LipL. **(B-D)** Synteny analysis of *pta* and *lipL* genes. **(B)** Phylogenetic tree of Gram-negatives, including *pta* synteny analysis adjacent to the relevant microorganism. **(C)** Phylogenetic tree of Gram-positives and *M. tuberculosis*, including *pta* synteny analysis adjacent to the respective microorganism. **(D)** Phylogenetic tree of *Staphylococci* including *pta* synteny analysis. **(E)** Normalized and mapped transcript reads across the *pta*-*lipL* region in WT and a Δ*lipL* mutant. **(F)** qRT-PCR analysis of RNA extracted from WT, Δ*pta*, and Δ*lipL* strains at 6 hours of growth. ****, p < 0.0001 by one-way ANOVA with Tukey’s post hoc test. qRT-PCR experiments were conducted with two independent biological replicates in technical triplicate. Boxplots indicate the median and quartiles. Whiskers indicate the range.

*S. aureus* preferentially uses glycolysis to metabolize glucose (Fig 1A) [4,19,43]. The lipoic acid-dependent PDH complex serves as a major exit point from the glycolytic pathway, where the decarboxylation of pyruvate leads to the generation of acetyl-CoA. During periods of carbon overflow *in vitro*, acetyl-CoA is predominantly shuttled into the Pta-AckA pathway to generate ATP and acetate via substrate-level phosphorylation (Fig 1A), in part because carbon catabolite repression suppresses TCA cycle gene expression [44,45]. Following glucose consumption, acetate is reassimilated via acetyl-CoA synthase (Acs), for use in the TCA cycle (Fig 1A). Central to glycolysis and TCA cycle function is LipL-dependent lipoylation of the E2 subunits of PDH and OGDH (Fig 1A). In a prior study, we determined that the *lipL* (SAUSA300_0571) open reading frame begins two nucleotides downstream from the stop codon of *pta* (SAUSA300_0570), implying a gene pairing that links lipoyl transfer activity with the Pta-AckA pathway [26]. Considering *pta* and *ackA* are genetically linked in classically studied microorganisms such as *Escherichia coli*, we wondered if this unusual *pta*-*lipL* linkage observed in *S. aureus* was widespread. We conducted a comparative synteny analysis of the *pta* gene environment. We surveyed representative Gram-negative or Gram-positive bacterial pathogens and *Mycobacterium tuberculosis*, as well as commensals *Lactococcus lactis* subs*. cremoris, Bacillus subtilis*, and *E. coli*, and found that most Gram-negative pathogens surveyed were likely to have *ackA* and *pta* positioned in an operon, like *E. coli* (Fig 1B). By contrast, analysis of *M. tuberculosis* and Gram-positive pathogens showed that *pta* and *lipL* were in close genetic proximity for some Gram-positives (*Bacillus anthracis* and *S. aureus*), whereas others were positioned at greater than 70kb nucleotide distances (~70kb for *Streptococcus pyogenes*, ~ 2500kb for *Listeria monocytogenes*, and ~300kb for *Enterococcus faecalis*) (Fig 1C). *S. aureus* was the only species with *pta* and *lipL* genetically linked in a putative operon, potentially implying a unique genetic arrangement for *Staphylococci* (Fig 1C). Indeed, coagulase-positive and negative *Staphylococci* all have the same *pta*-*lipL* organization, indicating it is a conserved feature of the genus (Fig 1D).

To establish that *S. aureus* co-transcribes *pta* and *lipL*, we conducted RNA sequencing of WT *S. aureus* and a Δ*lipL* mutant grown to late exponential phase. The read analysis indicated that *pta* and *lipL* transcripts consist of a single mRNA encoding both genes (Fig 1E). The deletion of *lipL* led to the generation of a truncated transcript but did not negatively impact *pta* expression (Fig 1E). To validate the RNAseq results, we measured transcript levels of *pta*, *lipL* and the junction between the *pta* and *lipL* genes via qRT-PCR. Transcript levels were similar between the *pta*, *lipL*, and the junction in WT *S. aureus* (Fig 1F). Junctional amplicons were not detected from the Δ*pta* and Δ*lipL* mutants (Fig 1F). Altogether, these data indicate that the genetic link between *pta* and *lipL* is conserved in Staphylococci and the genes are co-transcribed.

### Loss of either *pta* or *lipL* compromises acetate production

**Fig 2:**
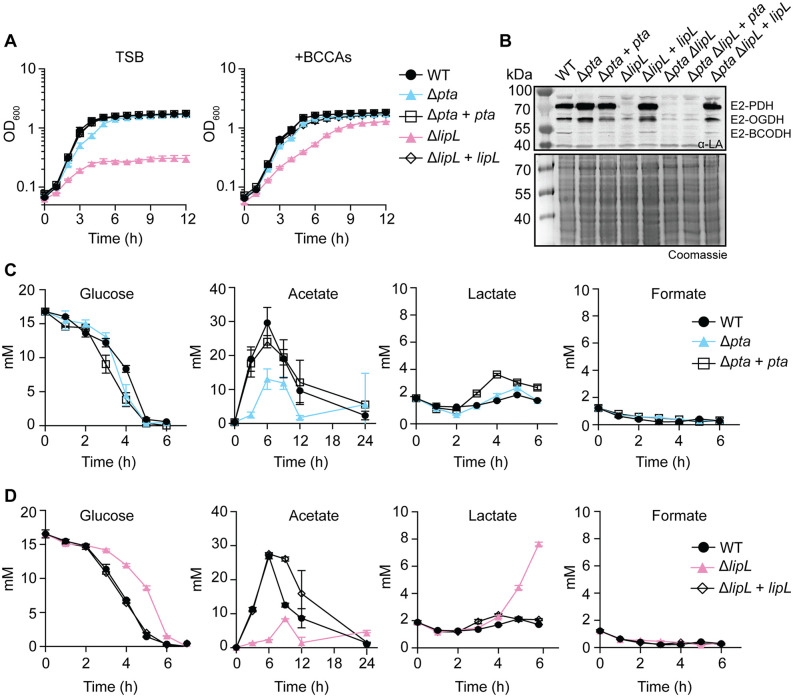
Loss of either *pta* or *lipL* compromises acetate production. **(A)** Growth of WT, Δ*pta*, Δ*lipL*, Δ*pta* + *pta*, and Δ*lipL* + *lipL* strains in TSB and TSB + BCCAs [10 mM isobutyric acid (IB), 9 mM 2-methylbutyric acid (2MB), 9 mM isovaleric acid (IV) + 10 mM sodium acetate (NaAc)] containing 14 mM glucose. **(B)** α-lipoic acid immunoblots of whole cell lysates from the indicated strains after 9-hours of growth in TSB medium. The presented blot and Coomassie-stained gel are representative of at least three independent experiments. **(C)** Quantification of glucose, acetate, lactate and formate from WT, Δ*pta*, Δ*pta* + *pta* culture supernatants over time in TSB with 14 mM glucose. **(D)** Quantification of glucose, acetate, lactate and formate from WT, Δ*lipL*, Δ*lipL* + *lipL* culture supernatants over time in TSB with 14 mM glucose and BCCA supplementation. Each metabolite quantification assay is representative of three independent experiments, with each timepoint measured in technical triplicate. Errors bars indicate standard deviation from the mean. Some lines on growth curve graphs (Δ*pta* + *pta*, and Δ*lipL* + *lipL*) are obscured because they overlap.

Given the evidence for co-transcription of *pta* and *lipL* in *S. aureus* and the role of Pta in overflow metabolism, we sought to determine how these genes contribute to acetate production via Pta-AckA and metabolic flux in general. We generated in-frame single and double deletions of *pta* and *lipL* in the methicillin-resistant *Staphylococcus aureus* (MRSA) strain LAC [46,47]. We complemented *pta* and *lipL* in single copy in the chromosome with constitutive expression [48], and subsequently determined if the loss of *pta* or *lipL* resulted in growth defects or shifts in metabolite production. The Δ*pta* mutant had a marginal delay in exit from logarithmic phase growth in tryptic soy broth (TSB) compared to WT (Fig 2A). A Δ*lipL* mutant, on the other hand, had compromised growth kinetics, including dramatically reduced growth in TSB, consistent with previous work from our group (Fig 2A) [1]. A significant component of the Δ*lipL* mutant growth defect in TSB is due to loss of LipL-dependent lipoylation of the BCODH complex, which is essential for branched-chain fatty acid synthesis [5]. Indeed, growth of Δ*lipL* mutant was substantially restored in TSB supplemented with branched-chain carboxylic acids (BCCAs) (isobutyric acid, 2-methylbutyric acid, and isovaleric acid) that bypass the requirement for BCODH or with use of the *lipL* complement strain [1,26] (Fig 2A). To test for defects in lipoylation of E2 subunits of enzyme complexes, we conducted immunoblots on whole cell lysates of the Δ*pta* and Δ*lipL* mutants and their complement strains grown in TSB + BCCAs. Consistent with prior results, the Δ*lipL* mutant had defective lipoylation compared to WT [26] (Fig 2B), whereas the Δ*pta* mutant had evidence for increased lipoylation on E2 subunits (Fig 2B). Thus, while lipoylation itself is not a direct effect of Pta, the degree of lipoylation on E2 subunits is increased in its absence (see below). These data indicate that the effects of *pta* and *lipL* on growth kinetics and lipoylation in broth are separable and largely driven by LipL-dependent effects on BCODH activity.

We next assessed glycolytic flux over time by measuring glucose and acetate levels in media containing 14 mM glucose under aerobic conditions to promote carbon overflow metabolism [28,49]. In addition to acetate, we measured lactate and formate as alternative pyruvate fermentation products in *S. aureus* [31,32,50]. As previously reported, glucose was rapidly consumed within 6 hours of growth along with a commensurate increase in acetate that peaked at 6 hours and was reassimilated in stationary phase (Fig 2C) [27,28]. The Δ*pta* mutant consumed all the glucose in the media by 6 hours with no differences compared to the WT strain (Fig 2C). Peak acetate levels from the Δ*pta* mutant were ~13 mM, a ~ 57% reduction compared to WT (Fig 2C). There were no differences in lactate or formate levels produced by the Δ*pta* mutant compared to WT (Fig 2C). Conversely, the Δ*lipL* mutant had a delay in the glucose consumption rate relative to WT (Fig 2D). Peak acetate production from the Δ*lipL* mutant was around 8 mM, a ~ 70% reduction compared to WT (Fig 2D). There was also a commensurate increase in lactate production by the Δ*lipL* mutant, which peaked at ~8 mM at 6 hours (Fig 2D). Growth and metabolite quantification assays using glucose-free media or low glucose supplementation (3.5 mM) established that acetate production requires LipL and that levels depend on the amount of glucose added. Compensatory increases in lactate production by a Δ*lipL* mutant are only observed when glucose is provided as a carbon source (14 mM and 3.5 mM) (S1 Fig and Fig 2D). Together, these data suggest that both Pta and LipL are required for optimal acetate fermentation during overflow metabolism and under conditions where glucose is limiting. While deletion of either *pta* or *lipL* leads to compensatory acetate production, presumably by other acetogenic pathways, lactate fermentation is only induced upon deletion of *lipL* in the presence of glucose, potentially to offset a pyruvate bottleneck during overflow that is associated with inactivation of PDH.

### LipL and Pta coordinate PDH activity and acetate production

**Fig 3:**
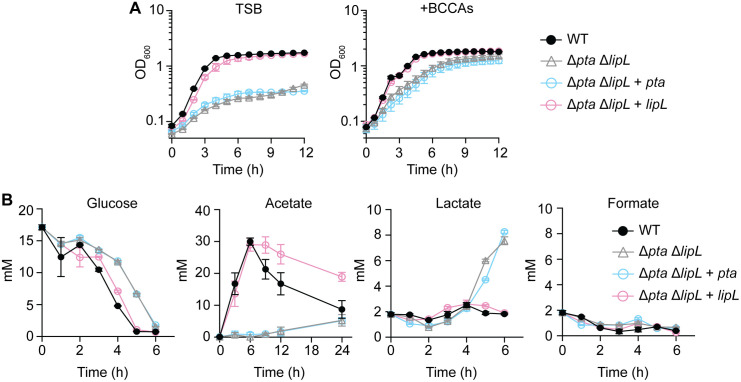
Pta and LipL coordinate PDH activity and acetate production. **(A)** Growth of WT, Δ*pta* Δ*lipL*, Δ*pta* Δ*lipL* + *pta*, and Δ*pta* Δ*lipL* + *lipL* strains in TSB and TSB + BCCAs [10 mM isobutyric acid (IB), 9 mM 2-methylbutyric acid (2MB), 9 mM isovaleric acid (IV) + 10 mM sodium acetate (NaAc)] containing 14 mM glucose. **(B)** Quantification of glucose, acetate, lactate, and formate from WT, Δ*pta* Δ*lipL*, Δ*pta* Δ*lipL* + *pta*, and Δ*pta* Δ*lipL* + *lipL* culture supernatants over time in TSB with 14 mM glucose and BCCA supplementation. Each metabolite quantification assay is representative of three independent experiments, with each timepoint measured in technical triplicate. Error bars indicate standard deviation from the mean.

*pta* and *lipL* are co-transcribed (Fig 1). The deletion of either gene alone shifts acetate production and metabolic flux either downstream (Δ*pta*) or upstream (Δ*lipL*) of PDH (Figs 1A and 2). We hypothesized that the tandem expression of *pta-lipL* might promote coordination of pyruvate decarboxylation with the delivery of acetyl-CoA to Pta-AckA to improve metabolic efficiency. To test this, we assessed the effect of loss of the entire *pta*-*lipL* locus on growth and metabolite production. The Δ*pta* Δ*lipL* mutant had severely compromised growth in TSB, which was largely complemented when TSB was supplemented with BCCAs or when using a Δ*pta* Δ*lipL* + *lipL* complement strain (Fig 3A). The Δ*pta* Δ*lipL* + *pta* strain did not restore growth in TSB without BCCAs (Fig 3A). Immunoblots on whole cell lysates of the Δ*pta* Δ*lipL* double mutant and complement strains revealed that only those strains lacking *lipL* had defective lipoylation profiles compared to WT (Fig 2B).

The WT and Δ*pta* Δ*lipL* + *lipL* strains consumed all glucose by 6 hours, whereas the Δ*pta* Δ*lipL* double mutant and Δ*pta* Δ*lipL* + *pta* had delays in glucose consumption (Fig 3B). Notably, the Δ*pta* Δ*lipL* double mutant did not produce additional acetate (Fig 3B). The Δ*pta* Δ*lipL* + *pta* strain mirrored this phenotype and acetate levels did not accumulate beyond ~5 mM, a ~ 83% reduction compared to peak acetate production in the WT and Δ*pta* Δ*lipL* + *lipL* strains (Fig 3B). Intriguingly, the Δ*pta* Δ*lipL* + *lipL* strain had delayed acetate reassimilation. Furthermore, compensatory lactate production by the Δ*pta* Δ*lipL* double mutant and Δ*pta* Δ*lipL* + *pta* strains was consistent with a redirection of pyruvate toward lactate fermentation (Fig 3B). Taken together, these data confirm that defects in growth associated with the Δ*pta* Δ*lipL* mutant are due to activities of LipL that are independent of Pta-AckA and flux through PDH. Furthermore, loss of both *pta* and *lipL* halts acetate production entirely, redirecting pyruvate towards alternative fates, such as lactate fermentation. Constitutive expression of *lipL* favors alternative modes of acetate production that do not depend on Pta-AckA.

### Pta and LipL proteins directly interact

**Fig 4:**
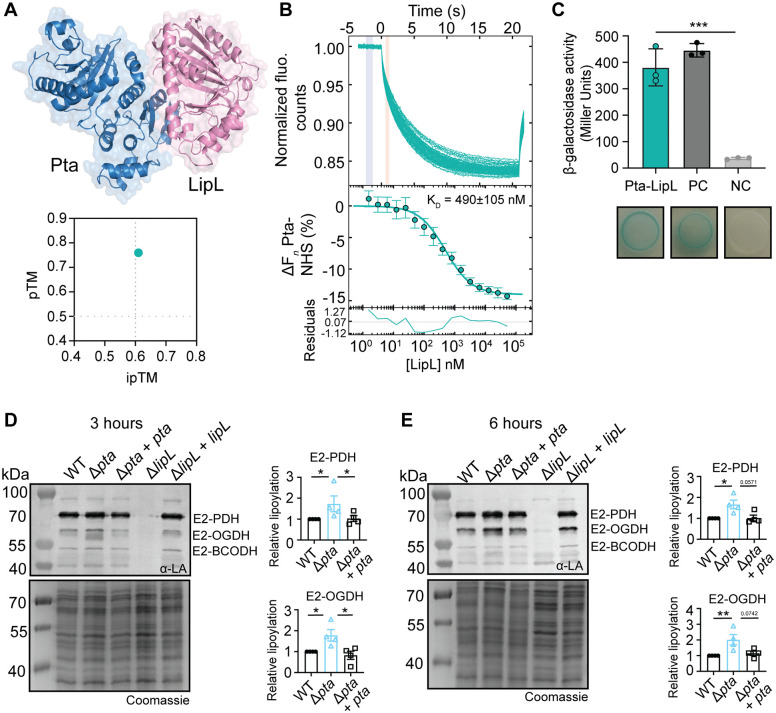
Pta and LipL proteins directly interact. **(A)** AlphaFold3 prediction of the interaction between Pta (blue) and LipL (pink). pTM (0.76) and an ipTM (0.61) are displayed graphically below the model. **(B)** Microscale thermogram of Pta-NHS titrated against the recombinant LipL. The top panel shows the thermophoretic time-traces of four independent experiments, and the blue and pink areas represent time spans used to obtain the fluorescence cold (F_c_) and hot (F_h_) regions, respectively. The middle panel shows the binding curve with the line of best fit using the 1:1 binding model with an error surface projection confidence of 95%. The residuals between the data and fit are shown in the bottom panel. *Kd* is presented as the mean ± standard deviation of four independent replicates. **(C)** Bacterial adenylate cyclase two-hybrid (BACTH) assay was used to assay an interaction between Pta and LipL. Graph displays β-galactosidase activity (Miller Units) from *E. coli* BTH101 co-transformed with pUT18C-*pta* and pKT25-*lipL*, pUT18C-zip and pKT25-zip (positive control – PC) or pUT18C and pKT25 vectors (negative control – NC). ***, p < 0.001 by one-way ANOVA with Tukey’s post hoc test. Cultures of *E. coli* BTH101strains co-transformed with the aforementioned plasmid combinations were also spotted on LB agar plates with X-Gal as an indicator. Data are representative of at least three independent experiments. **(D-E)** α-lipoic acid immunoblots of whole cell lysates from WT, Δ*pta*, and Δ*pta* + *pta* strains after 3 and 6-hours of growth in TSB medium. Densitometric quantification of E2-PDH and E2-OGDH bands in Δ*pta* and Δ*pta* + *pta* strains relative to WT bands. *, *p* < 0.05; **, p < 0.01 by Kruskal-Wallis test with Dunn’s post hoc analysis. The presented blots and Coomassie-stained gels are representative of four independent experiments. The mean and standard error of the mean are shown.

Prior functional and computational studies suggest that metabolic flux can be enhanced by protein-protein interactions and protein complex formation [13–15]. Considering our demonstration of the coordinated expression of *pta* and *lipL* and the necessity of both for optimal acetate production, we hypothesized there might be a direct interaction between Pta and LipL that could facilitate acetate fermentation. We first used AlphaFold3 to predict the likelihood of a direct interaction between Pta and LipL. The AlphaFold3 model predicted a potential interaction between Pta and LipL, with a template modeling of 0.76 and an interface predicted template modeling (ipTM) score of 0.61 (Fig 4A). Considering the ipTM score of the predicted interaction falls at the likelihood threshold for a potential interaction, we opted to validate this prediction by expressing and purifying Pta (Pta-6xHis) and LipL (LipL-6xHis) and conducting a biophysical assessment of a direct interaction using microscale thermophoresis (MST). Recombinant Pta was labeled with the amine-reactive dye, N-hydroxysuccinimide (NHS) and titrated against unlabeled LipL (Fig 4B). MST determined that Pta interacts with LipL in solution with moderate affinity (*Kd* of 490 + /- 105 nM) (Fig 4B). To determine if the proteins interact *in vivo*, we cloned the coding sequences of both *pta* and *lipL* into the adenylate cyclase-based Bacterial two-hybrid (BACTH) system [51,52]. Pta and LipL interacted strongly when expressed in *E. coli*, achieving similar levels of β-galactosidase activity as the leucine zipper positive control (Fig 4C). In considering the possibility that an interaction between Pta and LipL might improve efficiency of metabolic flux through PDH to Pta-AckA, we reasoned that loss of Pta would enhance the demand for LipL-mediated delivery of lipoic acid to E2 proteins of PDH and OGDH as a compensatory measure to drive carbon flux. Indeed, a Δ*pta* mutant had increased lipoylation on E2-PDH and E2-OGDH at 3 and 6 hours compared with WT and the Δ*pta* + *pta* strain (Fig 4D and 4E). This increase in lipoylation was not associated with an increase in *pdhC* (E2-PDH) or *sucB* (E2-OGDH) transcripts at 3 or 6 hours (S2A Fig), suggesting increased delivery of lipoic acid to E2-PDH and E2-OGDH by LipL. Collectively, these results indicate Pta and LipL directly interact *in vitro* and *in vivo*. Furthermore, the loss of Pta results in increased delivery of lipoic acid to E2-PDH and E2-OGDH as a compensatory measure.

### The coordinated activity of Pta and LipL is important during infection of the skin, whereas additional functions of LipL promote systemic infection

**Fig 5:**
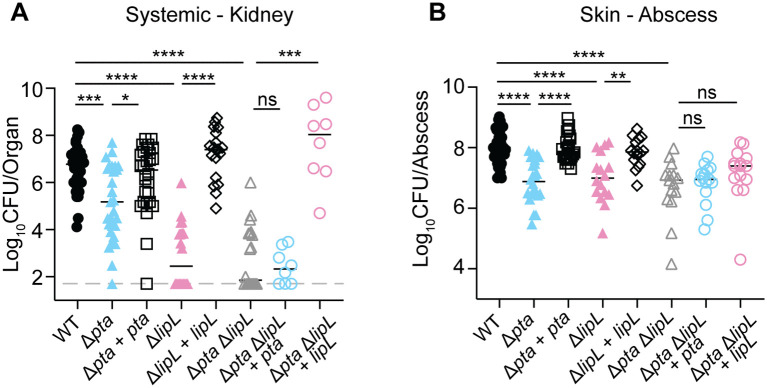
The coordinated activity of Pta and LipL is important during infection of the skin, whereas additional functions of LipL promote systemic infection. **(A)** Bacterial burden in the kidneys of mice 96 hours after bloodstream infection with 1.0 x 10^7^ CFU of WT, Δ*pta*, Δ*pta* + *pta*, Δ*lipL*, Δ*lipL* + *lipL*, Δ*pta* Δ*lipL*, Δ*pta* Δ*lipL* + *pta, and* Δ*pta* Δ*lipL* + *lipL* strains. Animal numbers displayed are as follows: WT, N = 36; Δ*pta*, N = 29; Δ*pta* + *pta*, N = 28; Δ*lipL*, N = 20; *ΔlipL* + *lipL*, N = 19; Δ*pta* Δ*lipL*, N = 20; Δ*pta* Δ*lipL* + *pta*, N = 8; and Δ*pta* Δ*lipL* + *lipL*, N = 8. ns, not significant; *, p < 0.05; ***, p < 0.001; ****, p < 0.0001 by Kruskal-Wallis test with Dunn’s post hoc analysis. **(B)** Bacterial burden in the skin of mice 72 hours after intradermal infection with 1.0 x 10^7^ CFU of WT, Δ*pta*, Δ*pta* + *pta*, Δ*lipL*, Δ*lipL* + *lipL*, Δ*pta* Δ*lipL*, Δ*pta* Δ*lipL* + *pta*, and Δ*pta* Δ*lipL* + *lipL*. Animal numbers displayed are as follows: WT, N = 16; Δ*pta*, N = 16; Δ*pta* + *pta*, N = 16; Δ*lipL*, N = 16; Δ*lipL* + *lipL*, N = 16; Δ*pta* Δ*lipL*, N = 16; Δ*pta* Δ*lipL* + *pta*, N = 16; and Δ*pta* Δ*lipL* + *lipL*, N = 16. ns, not significant; **, p < 0.01; ****, p < 0.0001 by Kruskal-Wallis test with Dunn’s post hoc analysis. log_10_ CFU per organ or abscess is displayed for each infected mouse along with the median as a measure of central tendency. Graphs represent combined data from at least two independent experiments.

Previous work from our group found that LipL is required for optimal *S. aureus* infection in both a murine sepsis model and a murine skin and soft tissue infection model [26]. Follow up studies determined that *S. aureus* is mostly refractory to lipoic acid deficiency in murine skin but not kidneys on account of its ability to bypass LipL-dependent branched-chain fatty acid synthesis by incorporating host unsaturated fatty acids [5]. These results do not preclude the possibility that the coordinated activity of Pta and LipL that drives acetate fermentation might contribute to infection in one or both infection models. To test this, we infected mice intravenously or intradermally with WT, Δ*pta*, Δ*pta* + *pta*, Δ*lipL*, Δ*lipL* + *lipL*, Δ*pta* Δ*lipL*, Δ*pta* Δ*lipL* + *pta*, and Δ*pta* Δ*lipL* + *lipL* strains. At 96 hours post intravenous infection, kidneys from Δ*pta* mutant-infected animals had ∼100-fold fewer CFUs compared to WT (Fig 5A). By contrast, the kidneys from Δ*lipL* mutant-infected mice had ~ 10000-fold fewer CFUs (Fig 5A). The kidneys from Δ*pta* Δ*lipL*, and Δ*pta* Δ*lipL* + *pta* infected mice phenocopied Δ*lipL* mutant-infected mice with ~10000-fold fewer CFUs (Fig 5A). Systemic infection with Δ*pta* + *pta*, Δ*lipL* + *lipL, and* Δ*pta* Δ*lipL* + *lipL* complement strains restored CFUs in the kidney to nearly WT levels (Fig 5A). At 72 hours post intradermal infection, abscesses from mice infected either with Δ*pta*, Δ*lipL*, and Δ*pta* Δ*lipL* mutants phenocopied one another with ∼10-fold fewer CFUs compared to WT (Fig 5B). Infection with the Δ*pta* + *pta* and Δ*lipL* + *lipL* complement strains restored abscess CFUs to nearly WT levels (Fig 5B). Infection with either Δ*pta* Δ*lipL* + *pta* or Δ*pta* Δ*lipL* + *lipL* strains did not restore CFU to WT levels (Fig 5B). Considering the identical attenuation for Δ*pta*, Δ*lipL*, and Δ*pta* Δ*lipL*, Δ*pta* Δ*lipL* + *pta* or Δ*pta* Δ*lipL* + *lipL* strains during infection of the skin, we conclude that the coordinated activity between Pta and LipL is required for *S. aureus* survival at this site. In contrast, during systemic infection, the contributions of Pta and LipL to virulence are separable and dominated by independent roles for LipL in promoting branched fatty acid synthesis, as we have previously published [5].

### Loss of *lipL* induces significant transcriptional changes in *S. aureus*

**Fig 6:**
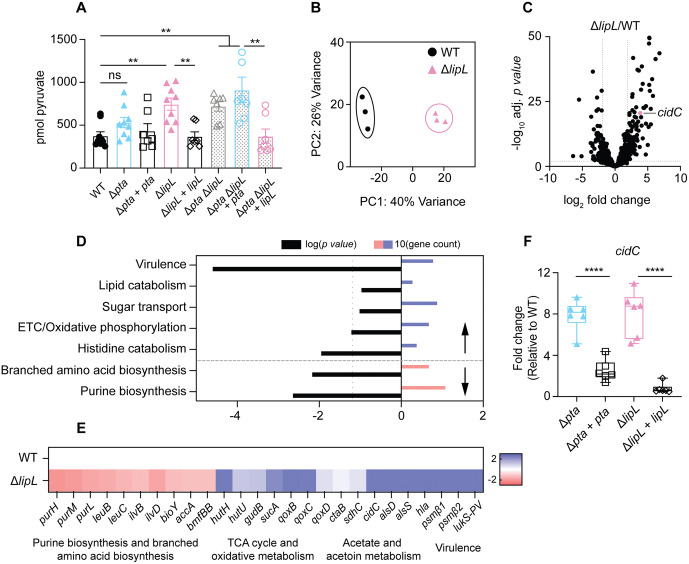
Loss of *lipL* induces significant transcriptional changes in *S. aureus.* **(A)** Quantification of intracellular pyruvate from WT, Δ*pta*, Δ*lipL*, Δ*pta* + *pta*, Δ*lipL* + *lipL*, Δ*pta* Δ*lipL*, Δ*pta* Δ*lipL* + *pta, and* Δ*pta* Δ*lipL* + *lipL* strains after 3 hours of growth in TSB. ns, not significant; **, *p* < 0.01 by Kruskal-Wallis test with Dunn’s post hoc analysis. Graphs represent combined data from three independent experiments. **(B)** Principal component analysis (PCA) of RNA sequencing datasets for the WT and Δ*lipL* mutant triplicate samples. **(C)** Volcano plot of transcriptional changes observed in a Δ*lipL* mutant compared to WT *S. aureus* during late-exponential phase growth. Each dot represents one gene. Vertical dotted lines represent log_2_ fold change = -2 and 2 cut-offs. Horizontal dotted line represents adj. p value = 0.05. **(D)** Analysis of upregulated and downregulated pathways in a Δ*lipL* mutant compared to WT. Black bars represent log(p-value); vertical dotted line is p-value < 0.05 threshold of significance as determined by NIH DAVID Bioinformatics pipeline. Blue bars represent the number of downregulated genes associated with the respective pathway, and pink bars represent the number of upregulated genes associated with the respective pathway. **(E)** Fold change of specific genes in the Δ*lipL* mutant compared to WT. (F) qRT-PCR analysis of RNA extracted from WT, Δ*pta*, Δ*lipL*, Δ*pta* + *pta*, and Δ*lipL* + *lipL* strains after 3 hours of growth in TSB. ****, p < 0.0001 by one-way ANOVA with Tukey’s post hoc test. qRT-PCR experiments were conducted with two independent biological replicates in technical triplicate. Boxplots indicate the median and quartiles. Whiskers indicate the range.

Our data suggest that a block to exit from glycolytic metabolism at PDH redirects metabolic flux on account of pyruvate accumulation in the cell. To test this, we quantified intracellular pyruvate levels from WT, Δ*pta*, Δ*pta* + *pta*, Δ*lipL*, Δ*lipL* + *lipL*, Δ*pta* Δ*lipL*, Δ*pta* Δ*lipL* + *pta*, and Δ*pta* Δ*lipL* + *lipL* strains at 3 hours. We found that all strains harboring a Δ*lipL* mutation had approximately twice the amount of intracellular pyruvate compared to WT (~745 pmol for a Δ*lipL* mutant versus ~363 pmol for WT) (Fig 6A). The redirection of pyruvate metabolism is further substantiated by increases in lactate and Pta-AckA - independent generation of acetate (Figs 2D and 3B). Given the importance of LipL in activating the PDH, OGDH, and BCODH complexes [1,26], we hypothesized that, in addition to the shifts in glucose metabolism at the PDH node, LipL might also drive a metabolic landscape that is shifted in a Δ*lipL* mutant on account of disruptions to TCA cycle activity and branched fatty acid synthesis. To test this possibility, we grew WT and Δ*lipL* mutant strains to late-exponential phase and performed bulk RNA-sequencing. Our results confirmed several major changes in gene expression patterns between the WT and Δ*lipL* mutant (Fig 6B and 6C). The Δ*lipL* mutant significantly upregulated genes involved in virulence, histidine catabolism and oxidative metabolism and significantly downregulated genes involved in purine nucleotide biosynthesis and branched-chain amino acid biosynthesis (Fig 6D). Specifically, the loss of *lipL* increased expression of several cytoxin genes (*lukS-PV, hla, psmβ1, and psmβ2)*, *gudB* (glutamate dehydrogenase), the *hut* operon (histidine uptake) and the *qox* operon (electron transport chain), while decreasing expression of the *pur* operon (purine biosynthesis), as well as the *leu* and the *ilv* operons (branched-chain amino acid biosynthesis) (Fig 6E). Furthermore, we saw substantially increased levels of the pyruvate oxidase *cidC* (SAUSA300_2477) (Fig 6C, 6E and 6F). CidC generates acetate directly from pyruvate and does not require PDH activity [37]. We also saw increased levels of *alsD* transcripts (Fig 6E). AlsSD diverts carbon flow towards neutral byproducts to prevent CidC-mediated acidification of the cell [36]. To validate our RNAseq results, we assessed *cidC* transcript levels in WT, Δ*pta*, Δ*lipL*, Δ*pta + pta*, and Δ*lipL + lipL* strains via qRT-PCR. Indeed, we found a ~ 8-fold increase of *cidC* transcripts in both the Δ*pta* and Δ*lipL* mutants (Fig 6F). Overall, RNAseq analysis confirmed that the loss of *lipL* significantly alters the *S. aureus* transcriptome, including changes to pathways involved in virulence, oxidative metabolism, amino acid metabolism, and nucleotide biosynthesis. Furthermore, the RNAseq dataset revealed increased levels of *cidC* transcripts, which were confirmed by qRT-PCR in both the Δ*pta* and Δ*lipL* mutants.

### The deletion of both *pta* and *cidC* significantly reduces acetate production by *S. aureus*

**Fig 7:**
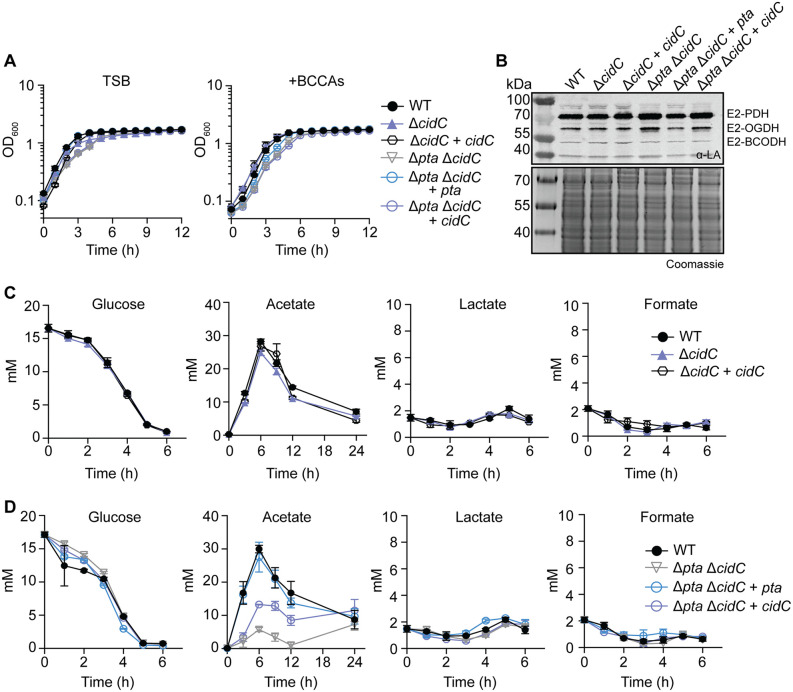
The deletion of both *pta* and *cidC* significantly reduces acetate production by *S. aureus.* **(A)** Growth of WT, Δ*cidC*, Δ*pta* Δ*cidC*, Δ*cidC* + *cidC*, Δ*pta* Δ*cidC* + *pta*, and Δ*pta* Δ*cidC* + *cidC* strains in TSB and TSB + BCCAs [10 mM isobutyric acid (IB), 9 mM 2-methylbutyric acid (2MB), 9 mM isovaleric acid (IV) + 10 mM sodium acetate (NaAc)] containing 14 mM glucose. **(B)** α-lipoic acid immunoblots of whole cell lysates from the indicated strains after 9-hour subculture growth in TSB medium. The presented blot and Coomassie-stained gel are representative of three independent experiments. **(C)** Quantification of glucose, acetate, lactate, and formate from WT, Δ*cidC, and* Δ*cidC* + *cidC* culture supernatants over time in TSB with 14 mM glucose. **(D)** Quantification of glucose, acetate, lactate, and formate from WT, Δ*pta* Δ*cidC*, Δ*pta* Δ*cidC* + *pta*, and Δ*pta* Δ*cidC* + *cidC* culture supernatants over time in TSB with 14 mM glucose. Each metabolite quantification assay is representative of three independent experiments, with each timepoint measured in technical triplicate. Error bars indicate standard deviation from the mean.

Prior studies showed that inactivation of the Pta-AckA pathway led to redirection of carbon into the TCA cycle [28,53]. Furthermore, loss of LipL or Pta leads to transcriptional changes that are suggestive of premature entry into the TCA cycle, increased oxidative metabolism, and increased acetate production by CidC (Fig 6F) [28]. Increased *cidC* levels in both Δ*pta* and Δ*lipL* strains signify demand for acetate production in *S. aureus* regardless of whether the block occurs before or after PDH. To confirm prior studies and test if Pta and CidC are the primary sources of acetate, we generated an in-frame deletion of *cidC* in both WT and a Δ*pta* mutant of *S. aureus*. The Δ*cidC* mutant had no effect on growth in TSB, whereas a Δ*pta* Δ*cidC* double mutant had a marginal delay in transition to stationary phase growth (Fig 7A), like the Δ*pta* single mutant (Fig 2A). The Δ*pta* Δ*cidC* and Δ*pta* Δ*cidC* + *cidC* strains, but not the Δ*cidC* or the Δ*pta* Δ*cidC* + *pta* strains had evidence of increased lipoylation on E2 subunits consistent with more lipoylation occurring in the absence of Pta, but not CidC (Fig 7B).

When measuring glucose, acetate, lactate, and formate levels, we noted the Δ*cidC* mutant consumed all glucose in the media by 6 hours with no difference compared to the WT (Fig 7C). Peak acetate production was ~ 30 mM for all strains (Fig 7C). Furthermore, there was negligible production of lactate and formate (Fig 7C). The Δ*pta* Δ*cidC* double mutant also consumed all glucose by 6 hours and had no delay in the glucose consumption compared to the WT (Fig 7D). Consistent with previous reports, there was negligible production of acetate from the Δ*pta* Δ*cidC* double mutant (~5 mM, a ~ 83% reduction compared to WT). (Fig 7D) [28]. Peak acetate production from the Δ*pta* Δ*cidC* + *cidC* was ~ 13 mM (Fig 7D), whereas acetate production from Δ*pta* Δ*cidC* + *pta* was the same as WT (Fig 7D). There was no lactate or formate produced, consistent with our earlier observations that compensatory lactate fermentation only occurs when PDH is inactivated (Fig 7D). These data corroborate prior work and indicate that *cidC* is dispensable for *S. aureus* growth in broth yet is activated to compensate for the loss of Pta to promote acetate production (compare Figs 2C and 7D) [28,36].

### Acetate production is required for *S. aureus* infection

**Fig 8:**
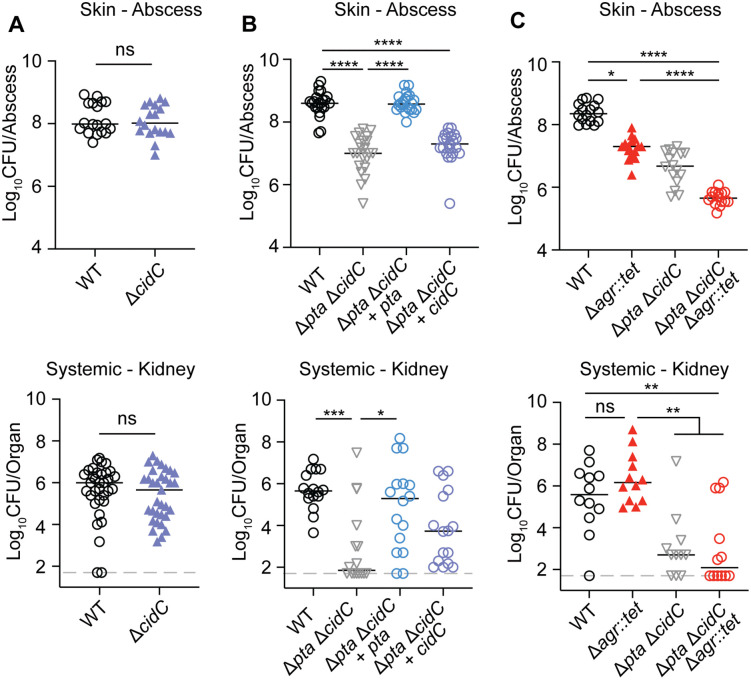
Acetate production is required for *S. aureus* infection. **(A)** Upper panel - Bacterial burden in the skin of mice 72 hours after intradermal infection with 1.0 x 10^7^ CFU of WT and Δ*cidC* strains. Animal numbers displayed are as follows: WT, N = 18; Δ*cidC*, N = 18. Lower panel - Bacterial burden in the kidneys of mice 96 hours after bloodstream infection with 1.0 x 10^7^ CFU of WT, Δ*cidC* strains. Animal numbers displayed are as follows: WT, N = 34; Δ*cidC*, N = 33. NS, not significant. Statistics were calculated by Mann-Whitney test. **(B)** Upper panel - Bacterial burden in the skin of mice 72 hours after intradermal infection with 1.0 x 10^7^ CFU of WT, Δ*pta* Δ*cidC*, Δ*pta* Δ*cidC* + *pta*, and Δ*pta* Δ*cidC* + *cidC* strains. Animal numbers displayed are as follows: WT, N = 29; Δ*pta* Δ*cidC*, N = 29; Δ*pta* Δ*cidC* + *pta*, N = 28; Δ*pta* Δ*cidC* + *cidC*, N = 28. Lower panel - Bacterial burden in the kidneys of mice 96 hours after bloodstream infection with 1.0 x 10^7^ CFU of WT, Δ*pta* Δ*cidC*, Δ*pta* Δ*cidC* + *pta*, and Δ*pta* Δ*cidC* + *cidC* strains. Animal numbers displayed are as follows: WT, N = 16; Δ*pta* Δ*cidC*, N = 16; Δ*pta* Δ*cidC* + *pta*, N = 16; Δ*pta* Δ*cidC* + *cidC*, N = 16. ***, p < 0.001; ****, p < 0.0001 by Kruskal-Wallis test with Dunn’s post hoc analysis. **(C)** Upper panel - Bacterial burden in the skin of mice 72 hours after intradermal infection with 1.0 x 10^7^ CFU of WT, Δ*agr::tet*, Δ*pta* Δ*cidC*, and Δ*pta* Δ*cidC* Δ*agr::tet* strains. Animal numbers displayed are as follows: WT, N = 16; Δ*agr::tet*, N = 16; Δ*pta* Δ*cidC*, N = 16; Δ*pta* Δ*cidC* Δ*agr::tet*, N = 16. Lower panel - Bacterial burden in the kidneys of mice 96 hours after bloodstream infection with 1.0 x 10^7^ CFU of WT, Δ*agr::tet*, Δ*pta* Δ*cidC*, and Δ*pta* Δ*cidC* Δ*agr::tet* strains. Animal numbers displayed are as follows: WT, N = 12; Δ*agr::tet*, N = 12; Δ*pta* Δ*cidC*, N = 12; Δ*pta* Δ*cidC* Δ*agr::tet*, N = 12. *, p < 0.05; **, p < 0.01; ****, p < 0.0001 by Kruskal-Wallis test with Dunn’s post hoc analysis. log_10_CFU per organ or abscess is displayed for each infected mouse along with the median as a measure of central tendency. All graphs represent combined data from at least two independent experiments.

In prior studies, CidC was implicated in the pathogenesis of biofilm-associated infection in the heart, suggesting CidC has a role in virulence [36]. Furthermore, the increase of *cidC* transcripts in the Δ*pta* and Δ*lipL* mutants (Fig 6F) led us to hypothesize that acetate generation via CidC could be an important compensatory strategy to promote *S. aureus* survival *in vivo*, especially if acetate generation in and of itself were important for infection. To begin to test this possibility, we determined if *cidC* was required for *S. aureus* bloodstream and skin infection using planktonic cultures. We infected mice intravenously or intradermally with WT, Δ*cidC*, and Δ*cidC* + *cidC* strains. At 96 hours post intravenous infection, we isolated and homogenized kidneys from Δ*cidC* mutant-infected animals and found no difference in CFUs compared to WT (Fig 8A). At 72 hours post intradermal infection, there was also no difference in CFUs between strains (Fig 8A). We then infected mice intravenously or intradermally with WT, Δ*pta* Δ*cidC*, Δ*pta* Δ*cidC + pta*, and Δ*pta* Δ*cidC* + *cidC* strains. After systemic infection, the kidneys from Δ*pta* Δ*cidC* double mutant-infected animals had ∼10000-fold fewer CFUs compared to WT (Fig 8B). This result was in stark contrast to a Δ*pta* mutant, which had a ~ 100-fold reduction in CFUs (Fig 5A). Complementation of the double mutant with *pta* restored kidney CFUs to nearly WT levels (Fig 8B), whereas complementation of the double mutant with *cidC* partially restored virulence to levels previously observed for a Δ*pta* mutant (~100-fold fewer CFUs compared to WT) (Fig 8B). At 72 hours post intradermal infection, we found ∼100-fold fewer CFUs were recovered from the abscesses of Δ*pta* Δ*cidC* double mutant compared to WT (Fig 8B). Like the kidneys, complementation with *pta* restored CFU levels to WT levels (Fig 8B), whereas complementation with *cidC* in the skin was not evident (Fig 8B).

Among the pathways significantly upregulated in RNAseq analysis of a Δ*lipL* mutant (Fig 6E) were genes involved in virulence. Toxin genes, including *hla*, *psm*β*1*, *psm*β*2*, and *lukS-PV*, were amongst the most significantly upregulated (Fig 6F). These toxin genes are all regulated by the Accessory Gene Regulatory (Agr) two-component quorum sensing system [54,55]. Despite the increase in Agr-dependent virulence gene expression, a Δ*lipL* mutant remained attenuated *in vivo* (Fig 5) arguing that virulence factor expression is not contributing to *in vivo* phenotypes in this strain. Nevertheless, this outcome prompted us test if *in vivo* phenotypes linked to acetate production were potentially due to changes in Agr activity. To test this possibility, we infected mice intravenously or intradermally with WT, Δ*agr::tet*, Δ*pta* Δ*cidC*, and Δ*pta* Δ*cidC* Δ*agr::tet* strains. At 96 hours post-intravenous infection, kidneys from Δ*agr::tet* mutant-infected animals had similar CFUs to WT (Fig 8C), consistent with previous reports [56,57]. Conversely, kidneys from Δ*pta* Δ*cidC* and Δ*pta* Δ*cidC* Δ*agr::tet* mutant-infected animals had identical reduction in CFUs compared to WT (∼10000-fold) (Fig 8C). At 72 hours post intradermal infection, abscesses from mice infected with Δ*agr::tet* mutant had ∼10-fold fewer CFUs compared to WT, which is consistent with previous literature [58] (Fig 8C). Abscesses from mice infected with Δ*pta* Δ*cidC* double mutant had ∼100-fold fewer CFUs compared to WT (Fig 8C). Mice infected with Δ*pta* Δ*cidC* Δ*agr::tet* triple mutant had ∼1000-fold fewer CFUs, demonstrating an additive effect (Fig 8C). Taken together, these data suggest that acetate production mediated by Pta and CidC is required for infection and is Agr independent.

## Discussion

**Fig 9:**
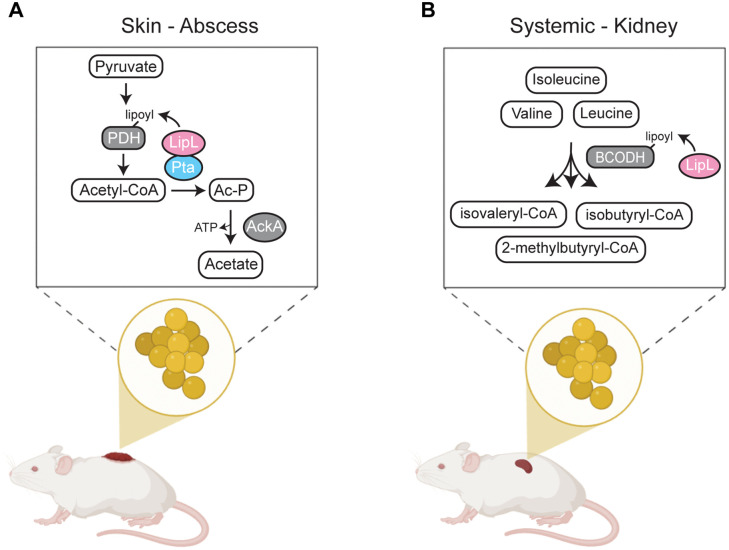
Proposed model of tissue-specific LipL-mediated requirement for virulence. **(A)** During skin and soft tissue infection, Pta and LipL activities coordinate metabolic flux through the Pta-AckA pathway. **(B)** During systemic infection, there is a dominant role for LipL-dependent lipoylation of BCODH that is vital for BCFA synthesis and *S. aureus* survival, which is distinct from roles for Pta in virulence. Figure Created in BioRender. Woods, R. (2026) https://BioRender.com/vbnk4gt.

In this study, we investigated the genetic and functional link between lipoic acid transfer and acetate production by *S. aureus*. We found that the genes encoding phosphotransacetylase, Pta, and the lipoic acid amidotransferase, LipL, are co-transcribed and that this regulatory arrangement is conserved amongst *Staphylococci*. At the protein level, Pta and LipL directly interact and their activities promote acetate fermentation (Pta-AckA) over oxidative metabolism. Deletion of either Pta or LipL significantly hindered acetate production, leading to an intracellular buildup of pyruvate and compensatory shifts in metabolism. This includes a substantial increase in *cidC* transcript, encoding the pyruvate oxidase, CidC, which generates acetate from direct decarboxylation of pyruvate. The coordinated function of Pta and LipL is required for infection of the skin, whereas the roles of Pta and LipL in systemic infection are separable (Fig 9). Moreover, we found that acetate production by Pta and CidC was required for *S. aureus* infection in both systemic and skin infections. Altogether, our data highlight a previously unidentified mechanism to regulate carbon flow by integrating the dynamic delivery of lipoic acid with metabolite shuttling. This work provides a compelling example of how metabolic adaptations that coordinate nutrient flux are beneficial for *S. aureus* survival in the host by allowing the bacterium to optimize its nutritional resources.

In prior work, we found that the *lipL* open reading frame is positioned two nucleotides downstream of the *pta* gene in *S. aureus*, suggesting the two genes are co-regulated at the transcriptional and likely translational levels (Fig 1E) [26]. RNAseq and qRT-PCR data validated the presence of a single *pta-lipL* transcript (Fig 1E and 1F). This finding raised the question of the frequency with which *lipL* and *pta* genes coincide. *In silico* synteny analyses herein revealed that, within the Gram-positive pathogens assessed, some had *lipL* in close genetic proximity to *pta* (Fig 1B-1D). This phenomenon was not observed in representative Gram-negative pathogens, where, in most instances, *ackA* and *pta* are co-transcribed (Fig 1B). The evolutionary differences between the lipoic acid biosynthesis and salvage pathways of Gram-positive and Gram-negative bacteria offer some insight into these *in silico* results. The lipoic acid biosynthesis pathway in *E. coli* and other Gram-negatives consists of two enzymes, LipA and LipB [25,59,60]. The octanoyltranferase, LipB, attaches octanoic acid directly to E2 and H protein subunits, followed by sulfur insertion by LipA. Thus, lipoic acid is synthesized directly on each metabolic enzyme complex [25,59,60]. Notably, Gram-negatives do not encode amidotransferases such as LipL and thus do not perform lipoyl relay between E2 and H proteins [25]. On the other hand, Gram-positives harbor a more complex lipoic acid biosynthesis pathway that includes LipM, a transferase that delivers octanoic acid to the H protein of GcvH, the sole site of sulfur insertion by LipA [25,61,62]. The transfer of lipoic acid from GcvH to other enzyme complexes is mediated by LipL. Without LipL, complete transfer to E2 subunits does not occur [1,26,62]. Furthermore, the presence of LipL confers a dynamic flexibility that potentially allows for dynamic redistribution of lipoic acid based on the metabolic state of the cell [1,25,26,62]. The absence of LipL in Gram-negatives and direct synthesis on each enzyme complex precludes the regulatory advantages offered by a lipoyl relay pathway. These fundamental differences may explain why lipoic acid synthesis and carbon overflow pathways are only linked in Gram-positives. Indeed, the transcriptional coupling of *pta* and *lipL* in Staphylococci hinted at a direct coupling of lipoic acid transfer and acetate fermentation that was validated in this work.

Assays of acetate levels in Δ*pta* and Δ*lipL* mutants revealed different degrees of impairment in acetate production, where a Δ*pta* mutant produced more acetate over time compared to a Δ*lipL* mutant (Fig 2C and 2D). This is likely accounted for by the fact that a Δ*lipL* mutant also produces substantial lactate, suggesting that blockade upstream of PDH necessitates redirection of pyruvate through both lactate fermentation and CidC (Fig 2D). In support of this point, the Δ*lipL* mutant also had considerably higher pyruvate levels (Fig 6A). In addition to these observations that center on pyruvate accumulation, our RNA sequencing data also showed that loss of *lipL* led to a global change to the transcriptome including upregulation of genes involved in histidine catabolism (*hut* operon) and oxidative metabolism (*qox* operon) and downregulation of genes involved in branched-chain amino acid biosynthesis (*leu* and *ilv* operons) and purine biosynthesis (*pur* operon) (Fig 6D and 6E). The downregulation of genes involved in branched-chain amino acid biosynthesis was accompanied by the downregulation of *bmfBB* (encoding E2-BCODH), *accA* (a subunit of acetyl-CoA carboxylase), and *bioY* (biotin transporter) (6E). These results suggest that a Δ*lipL* mutation leads to reduced branched-chain fatty acid biosynthesis since *bmfBB* encodes the E2 subunit of the enzyme complex that generates the coA derivatives required for branched chain fatty acid synthesis, while acetyl-CoA carboxylase is required for fatty acid synthesis itself with the biotin transporter aiding in acquisition of biotin, an essential cofactor for this process [63–65]. Conversely, upregulation of the *hut* operon suggests there is increased TCA cycle activity in a Δ*lipL* mutant because it is directly involved in the catabolism of histidine to glutamate, which is directly fed into the TCA cycle via *gudB* [45]. Indeed, *gudB* transcript levels were also elevated in a Δ*lipL* mutant relative to WT (Fig 6E). The shift toward a transcriptional profile that favors TCA cycle activity in a Δ*lipL* mutant is further supported by the fact that, of the lipoylated E2 subunits, only E2-OGDH maintains its lipoylation status in the absence of LipL (Fig 2B) [1,5,26]. The residual lipoylation of E2-OGDH is mediated by the *S. aureus* encoded lipoic acid ligases, LplA1 and LplA2 [1,66]. Furthermore, our RNAseq data showed increased *sucA* (encodes E1-OGDH) transcripts (Fig 6E), supporting the idea that TCA cycle activity is increased in a Δ*lipL* mutant (Fig 2B). A Δ*lipL* mutant also had increased expression of genes with roles in oxidative metabolism (Fig 6D) such as the *qox* operon, *sdhC* and *ctaB* (Fig 6E). The *qox* operon is involved in aerobic respiration, *sdhC* is a gene involved in both the TCA cycle and the electron transport chain, and *ctaB* produces the heme O cofactor required for QoxABCD terminal oxidase function [67–71]. All together these data support the idea that LipL has a key role in precisely modulating *S. aureus* metabolic flux, favoring acetate fermentation while limiting the TCA cycle and oxidative metabolism.

Our RNAseq analysis also uncovered several other genes that were upregulated in a Δ*lipL* mutant. We observed a significant increase in *cidC* transcripts, which was corroborated by qRT-PCR (Fig 6E and 6F). These results validate work from others, which suggests that induction of *cidC* represents a compensatory strategy to overcome intracellular pyruvate accumulation [28,36]. CidC is encoded in an operon alongside genes encoding the holin, CidA, and a potential support protein, CidB [34,72]. Transcription of the *cid* operon is under the control of multiple promoters and transcriptional regulators [33,72,73]. During exponential phase growth, sigma factor B (σB) induces the expression of *cidBC* [72]. During stationary phase, the LysR-type transcriptional regulator CidR induces expression of *cidABC* [39,33]. CidR induction of *cidABC* is known to promote maintenance of biofilm structure, where the holin activity of CidA aids in promoting bacterial cell death [39]. To determine if σB or CidR-dependent transcriptional upregulation was occurring in a Δ*lipL* mutant, we assessed our RNAseq dataset for *cidA* or *cidB* transcripts. Intriguingly, we found that the baseMean values for *cidC* were 3231.85, compared to 611.48 for *cidB* and 161.65 for *cidA*, suggesting the *cidC* transcript was upregulated independent of *cidA* and *cidB* in this scenario. Furthermore, a read analysis of *cidA*, *cidB*, and *cidC* showed a markedly higher read density across *cidC* compared to *cidA* and *cidB* (S2B Fig). The elevated *cidC* signal, coupled with its higher baseMean value, suggests the possibility of an alternative transcriptional start site for *cidC* that is independent of *cidAB*. Future work will address this possibility.

Work from Sadykov *et al.* highlights the importance of Pta and AckA for *S. aureus* fitness and metabolism. They found that the metabolome of both a Δ*pta* and a Δ*ackA* mutant is marked by a redirection of carbon flux into glycolysis, TCA cycle, pentose phosphate pathway, and amino acid biosynthetic pathways [28]. Our lipoylation profile data support this redirection of metabolic flux toward glycolysis and TCA cycle, wherein we noted increased lipoylation of E2-PDH and E2-OGDH in the Δ*pta* mutant (Fig 4D and 4E). Furthermore, we found that a Δ*pta* mutant does not have a commensurate increase in *pdhC*, or *sucB* transcripts (S2A Fig), suggesting the increase in lipoylation on E2-PDH and E2-OGDH is due to a redirection of lipoic acid transfer by LipL. Sadykov *et al.* also found that the loss of *pta* causes a fitness defect in which there is a delay in exponential phase growth, which we reproduced herein (Fig 2A) [28]. Interestingly, an *ackA* mutant in *S. aureus* has a faster growth rate during exponential phase compared to a *pta* mutant [28]. These results suggest the coordinated activity of Pta and LipL in *S. aureu*s, which occurs upstream of AckA activity, is more critical to overall fitness. Intriguingly, the reduced exponential phase growth rate of a Δ*pta* mutant compared to a Δ*ackA* mutant appears to be specific to *S. aureus*. Studies in other pathogens suggest a Δ*ackA* mutant has a more severe effect on growth rate compared to a Δ*pta* mutant, citing accumulation of acetyl-phosphate as the potential culprit [28,29,74,75]. This was the case for strains where *ackA*-*pta* were in an operon (*E. coli*), *pta* and *lipL* were in close genetic proximity but not linked (*B. subtilis*), and strains where *pta* and *lipL* were divergently transcribed (*B. anthracis*) [29,53,76]; thus, it is difficult to draw conclusions on the basis of these observations from gene positioning alone. Nevertheless, these prior studies coupled with our *in-silico* analysis (Fig 1B–1D) suggest that *Staphylococci* have adapted in ways that allow coordination of Pta and LipL activity to maintain fine-tuned control over metabolic flux through pathways that are critical for fitness.

Functional and computational studies suggest that metabolic flux is enhanced through improved channeling of substrates from one enzyme to another via protein complex formation and protein-protein interactions [13–15,77]. We used AlphaFold to predict the likelihood of a direct interaction between Pta and LipL and confirmed this model with *in vitro* and *in vivo* evidence (Fig 4). These studies suggest, at minimum, Pta and LipL may interact to drive metabolism in the direction of Pta-AckA. In these experiments, Pta and LipL had components of adenylate cyclase or 6x-his epitope tags appended to the C-terminus of each protein. Efforts to append CyaA fragments or epitope tags to the N-terminus prevented detection of an interaction between Pta and LipL (S2C Fig). Prior structural analysis of Pta in Gram-negative and positive organisms revealed that the protein contains two functional domains separated by an interdomain cleft, which is vital for the binding of acetyl-phosphate [78–80]. Crystal structures of Pta in Gram-positive organisms such as *B. subtilis* and *S. pyogenes* revealed that the N-terminus of Pta is a component of this interdomain cleft and may provide a potential reason why the N-terminus of Pta is not a productive site for epitope tags, as it is required for binding of substrates and presence in the interdomain cleft may preclude attachment of even small tags [78,80,81]. Future studies in our lab aim to identify specific residues critical for the Pta–LipL interaction and to test their impact on metabolite flux.

Additionally, previous biochemical assessments of Pta in Gram-negatives such as *E. coli* and *Salmonella enterica* Typhimurium found that the N-terminus of Pta serves a regulatory role through the allosteric binding of cofactors and substrates, such as NADH and pyruvate [82,83], whereas biochemical studies in Gram-positives reveal Pta activity is not altered by allosteric effectors [81]. Our inability to generate epitope tags of Pta or LipL that maintain protein-protein interaction characteristics suggests that the N-terminus of *S. aureus* Pta is, at minimum, important for interactions (S2C Fig). The increase in LipL-dependent lipoylation of E2-PDH and E2-OGDH in the absence of Pta could imply that LipL increases delivery of lipoic acid to E2 subunits due to increased metabolic demand or that Pta negatively impacts LipL function (Figs 2B, 4E–4D, and 7B). It is also possible that LipL improves Pta function. Future biochemical experiments will directly examine these possibilities [84–86]. Furthermore, our bacterial two-hybrid assays also detected evidence for an interaction between Pta and E2-PDH (S2C Fig); however, Alphafold modeling of the interaction had low predictive values (PTM of 0.47 and iPTM of 0.2) (S1D Fig). Nevertheless, the bacterial two-hybrid results may support the possibility of a larger complex between Pta, LipL, and PDH that improves substrate delivery. Additional evidence in support of this idea includes a protein interaction network analysis in *S. aureus* that identified an interaction between Pta and E2-PDH [87]. Further assessment of a potential Pta-LipL-PDH complex is warranted. We are currently pursuing these areas of investigation.

In a systemic infection model, the roles of Pta and LipL in virulence are separable (Fig 5A). While Pta contributes to infection in the kidney, the attenuation of Δ*lipL* and Δ*pta* Δ*lipL* mutants is far more dramatic, with the Δ*lipL* mutant and Δ*pta* Δ*lipL* mutants phenocopying each other (Fig 5A). LipL is known to transfer lipoic acid to enzyme complexes that extend beyond overflow metabolism pathways and include enzymes that facilitate branched-chain fatty acid synthesis (BCODH) and TCA cycle activity (OGDH). In prior work, we noted a strong dependency on branched-chain fatty acid synthesis during systemic infection that is bypassed in the skin via uptake of host unsaturated fatty acids [5]. Thus, the contribution of LipL to infection in the kidney is largely driven by deficiencies in branched-chain fatty acid synthesis, whereas Pta contributes to a lesser degree (Fig 5A). This conclusion is further supported by the finding that constitutive expression of *lipL*, but not *pta*, in a Δ*pta* Δ*lipL* mutant background nearly restores full virulence in the kidneys (Fig 5A). In contrast, Δ*pta*, Δ*lipL*, and Δ*pta* Δ*lipL* strains phenocopy one another (Fig 5B) for infection defects in the skin, implying a direct role for flux through PDH and toward Pta-AckA in skin infection. In agreement with this idea, complementation with either *pta* or *lipL* alone fails to restore full virulence in the skin (Fig 5B). These results further cement the importance of regulating acetate production during *S. aureus* skin infection (Fig 9).

Several studies have considered the roles of *S. aureus* acetate production genes during infection. One study found that infection with a Δ*ackA* mutant had reduced lesion area during skin infection of hyperglycemic mice [40]. Another study assessed a Δ*cidC* mutant in a rabbit model of infective endocarditis and found no difference between the Δ*cidC* mutant and WT at the primary site of the vegetation [36]. Instead, the authors found that a Δ*cidC* mutant had significantly fewer CFUs in secondary sites of infection, leading them to suggest CidC may play a role in dispersal from biofilms in the host [36]. Here, we find that a requirement of acetate production is not contingent on a high glycemic status (mice are on standard chow). Furthermore, our Δ*cidC* mutant infection data suggest that during planktonic growth, CidC alone is not required for infection. However, infection with a Δ*pta* Δ*cidC* strain leads to dramatic attenuation compared to either single deletion, implying compensatory demand for acetate production *in vivo* (Fig 8B). Altogether, this infection data highlights the critical importance of acetate-producing genes in several models of infection.

Recent work from Thurlow *et al.* determined that glycolytic flux enhances activity of the accessory gene regulatory (Agr) system [40]. Agr is a two-component system (TCS) that responds to changes in bacterial cell density and is a master regulator of virulence factors, controlling the expression of leukotoxins, hemolysins, lipases, and proteases [88–90]. Agr-dependent toxin production by *S. aureus* requires ATP production from processes such as overflow metabolism through Pta-AckA [40]. The requirement of glycolysis to enhance Agr-dependent gene expression is accounted for by a reduced affinity of AgrC, the sensor histidine kinase of the Agr system, for ATP [40,91,92]. Thus, ATP must be high for maximal production of Agr-dependent virulence factors in *S. aureus*. We found that a Δ*lipL* mutant increases oxidative metabolism to maintain energy production when flux through the PDH node is blocked (Fig 6E–6F). RNAseq analysis further revealed increased expression of Agr-regulated virulence genes in the Δ*lipL* mutant (Fig 6E–6F). Although not yet directly tested, these findings raise the possibility that ATP generated through oxidative metabolism might also promote Agr-dependent virulence factor production. Regardless, these studies further support the idea that the metabolic status of *S. aureus*, as defined by the nutritional environment, can have direct impacts on pathogenic potential.

The innate immune system serves as the body’s first line of defense against bacterial infections, with macrophages and neutrophils acting as key early responders that initiate the host immune response. Our data demonstrates that the loss of acetate production is detrimental for *S. aureus* survival in the host (Fig 8), suggesting that acetate might play a role in modulating the host response to infection. Given this, we suspect that *S. aureus*-derived acetate might alter the function of innate immune cells. Indeed, acetate is a ligand for the G-protein-coupled receptors FFAR2 and FFAR3, which are present on the surface of macrophages and neutrophils [93–96]. FFAR2 primes innate immune cells, facilitating productive immune responses during bloodstream, gastrointestinal, and pulmonary infections caused by both Gram-negative and Gram-positive pathogens [97–100]. More recent work from Xu *et al* showed that acetate and formate produced by *S. pyogenes* inhibited innate immune cell accumulation and cytokine production, which delayed host wound healing and bacterial clearance during soft tissue infections [101]. Thus, a paradox emerges in which acetate can either enhance or suppress the innate immune response. Given the importance of Pta and CidC for *S. aureus* infection (Fig 8), our data may imply a role for acetate in dampening the innate immune response. Ongoing work aims to study the potential impact of acetate on innate immune cells during *S. aureus* infections.

Collectively, this work supports the idea that *S. aureus* coordinates metabolic flux through transcriptional and post-transcriptional mechanisms to promote adaptation and survival during infection. These findings have implications for our understanding of coordinated enzyme complex assembly that drives the delivery of key metabolites to their targets and argue for a potential direct role for acetate production in bacterial virulence. Further, it highlights the importance of tissue-specific dependencies on major metabolic processes for virulence, which supports a critical examination of pathogenesis using several infection models.

## Methods

### Ethics statement

All animal experiments were conducted in ABSL2 facilities under Institutional Animal Care and Use Committee (IACUC)-approved protocols from the University of Illinois Chicago (Protocol #25–042) and Loyola University Chicago Health Sciences Division (Protocol #2020025), in accordance with United States Department of Agriculture (USDA), Public Health Service (PHS), and Office of Laboratory Animal Welfare (OLAW) guidelines. The University of Illinois Chicago holds a current PHS Animal Assurance (#A3460-01, through 04/30/2027), is Association for Assessment and Accreditation of Laboratory Animal Care (AAALAC) accredited (#000186, 04/01/2024), and USDA licensed (#33-R-0018). Loyola University Chicago holds a current PHS Animal Assurance (#A3460-01, through 02/28/2026), is AAALAC-accredited (#000180, 11/18/2022), and USDA-licensed (#33-R-0024). All procedures complied with institutional ethical standards.

### Phylogenetic tree construction and synteny analysis

The *pta* coding sequence was used in independent searches of the Seed analysis viewer (https://pubseed.theseed.org/FIG/seedviewer.cgi?page=Minimal) [102,103]. The *pta* gene in *Staphylococcus aureus* subsp. *aureus* USA300 used for search was taxon ID 367830; feature ID fig|367830.3.peg.650) and is annotated as phosphate acetyltransferase (EC 2.3.1.8). All genomes were compared by a standard search of the Seed database. The genomes of *M. tuberculosis* and selected Gram-positive and Gram-negative bacteria representing the most frequent genetic organizations of *pta*, *lipL*, and *ackA* were then chosen for subsequent analysis. Taxonomic assignments for these genomes were confirmed in NCBI (TaxIDs: 242231, 604162, 99287, 575584, 1051003, 272620, 83333, 196600, 187410, 451515, 224308, 1027396, 160490, 1773, 226185, 416870, 373153, 191218, 1385523, 1242971, 1155131, 904338, 1189311, 435837, 1134914, 342451). Phylogenetic trees were constructed using the selected genomes in PhyloT_v2_ (https://phylot.biobyte.de) and visualized using the interactive Tree of Life tool, iTOL_v7_ (https://itol.embl.de) [104].

### Bacterial strains and growth conditions

The strains used in this study are described in S1 Table. *Escherichia coli* strains were grown in Lysogeny Broth (LB) (BD Biosciences), and *S. aureus* strains were grown in Tryptic Soy Broth (TSB) (BD Biosciences). For experiments with no or low glucose (3.5 mM), *S. aureus* strains were growin in TSB without dextrose (BD Biosciences) with or without D-glucose supplementation (Amresco). Unless stipulated, strains were grown at 37°C, shaking at 200 RPM. For strains harboring a Δ*lipL* mutation, branched-chain carboxylic acids (BCCAs) (10 mM isobutyric acid [Sigma-Aldrich], 9 mM 2-methylbutyric acid [Alfa Aesar], 9 mM isovaleric acid [Sigma-Aldrich]) and 10 mM sodium acetate (NaAc; Sigma-Aldrich) were added to the media to promote growth [23,61]. When needed, media were supplemented with the following agents of selection: ampicillin (100 µg/ml), chloramphenicol (10 µg/ml), kanamycin (50 µg/ml), cadmium chloride (0.1 mM), anhydrous tetracycline (1 μg/ml) (AnTet), and sodium citrate (10 mM).

### Generation of in-frame deletion mutants

Oligonucleotide pairs (S2 Table) were designed to amplify fragments of ~500–1000 nucleotides upstream and downstream of the targeted genes for mutagenesis using *S. aureus* LAC genomic DNA as a template. Purified amplicons were then used as templates in a splicing by overlap extension (SOE) PCR to yield the final 1,000–2,000 bp amplicon containing a fusion of the upstream and downstream gene fragments. Each amplicon was digested with KpnI and SacI restriction endonucleases and ligated into the multiple cloning site (MCS) of pIMAY using T4 DNA ligase [105]. Ligation reactions were transformed into *E. coli* IM08B [46], and transformants containing the ligated amplicon were verified by PCR. Plasmids were purified from IM08B and subsequently transformed into *S. aureus* competent cells by electroporation (1800 V, 10 μF, 600 Ω, 2 mm cuvette). Mutagenesis was performed as previously described [47]. Briefly, *S. aureus* cells containing pIMAY plasmids were grown at 30°C to allow for plasmid replication. Plasmid recombination into the chromosome was induced at 37°C with selection on chloramphenicol. To facilitate plasmid excision, cells were returned to the permissive temperature for plasmid replication (30°C) and passaged without antibiotic to promote plasmid loss. Bacteria were then plated on TSA plates supplemented with AnTet and incubated at 30°C to counter-select for cells that still contain the pIMAY plasmid. Chloramphenicol sensitive and AnTet-resistant colonies were screened for the desired mutation by PCR.

### Generation of complement strains and phage transduction

Complementation strains were generated using the pJC1111 plasmid [48]. Genes for complementation were amplified from *S*. *aureus* genomic DNA using the primer pairs listed in S2 Table, while the constitutive *P*_HELP_ promoter was amplified from the pIMAY plasmid as template [47]. Purified amplicons were fused by SOE PCR and subsequently cloned into the MCS of pJC1111 using Sac1 and Sal1 restriction endonucleases for digestion and T4 DNA ligase for ligation. The recombinant plasmids were propagated in *E*. *coli* DH5α and transformed into SaPI-1 integrase-expressing *S*. *aureus* (RN9011) to allow single-copy integration in the chromosome at the SaPI-1 site [48]. Bacteriophage Φ11 was used to package the integrated complementation plasmids from RN9011, and the phage was used for transduction of genetic material into the recipient strain of interest, as previously described [106]. Briefly, a single colony of the RN9011 donor strain was grown overnight at 37°C with shaking. The overnight culture was diluted to an OD_600_ of 0.2 in TSB. 5 mL of diluted culture was added to 5 mL of TMG buffer (10 mM Tris pH 7.5, 5 mM MgCl_2_, 0.01% gelatin (v/v), 5 mM CaCl_2_) in a 15 mL conical tube containing 100 μL of Φ11 phage and incubated at room temperature overnight until lysis was complete. The following day, 100 μL of the filter sterilized phage lysate was added to an overnight culture of the recipient strain and incubated at 37°C with shaking for 20 minutes. The infected recipient was washed three times with 1 mL of 40 mM sodium citrate and then plated on TSA supplemented with 0.1 mM cadmium chloride and 40 mM sodium citrate to select for transductants. All transductants were confirmed by PCR.

### Generation of Δ*agr* Δ*pta* Δ*cidC* strain

A marked Δ*agr*::*tet* mutation was transduced into a Δ*pta* Δ*cidC* mutant strain, as described above, to generate the Δ*agr::tet* Δ*pta* Δ*cidC* mutant strain. The Δ*agr*::*tet* mutation spans the *agr* operon [58].

### Generation of 6x-Histidine tagged Pta and LipL expression plasmids

The *pta* and *lipL* genes were amplified from *S. aureus* LAC genomic DNA using the primer pairs in S2 Table. The resulting amplicons were cloned into the pET21a (Novagen) expression vector using Nde1 and Sal1 restriction endonucleases for digestion and T4 DNA ligase for ligation. The resulting plasmids were transformed into chemically competent *E. coli* DH5α. Plasmids were purified from DH5α and transformed into chemically competent *E. coli* lysY/I^q^ (NEB). All recombinant expression plasmids were verified by whole plasmid sequencing (Plasmidsaurus).

### Purification of 6x-Histidine-tagged proteins

To purify Pta-6xHis and LipL-6xHis, a single colony of *E. coli* lysY/I^q^ containing pET21a-*pta-6xHis* or pET21a-*lipL-6xHis* was used to start an overnight culture in 20 mL of LB at 37°C with shaking at 200 RPM. The bacteria were diluted (1:100) in 1 L of fresh LB and incubated at 37°C with shaking at 200 RPM until reaching an OD_600_ of ~0.3. Protein expression was induced by supplementing the culture with IPTG (0.1 mM) and incubating at 37°C for 3 hours with shaking at 200 RPM. Following induction, bacteria were collected by centrifugation at 10000 RPM for 20 min, and pellets were stored at -80°C until use. Frozen bacterial pellets were thawed and resuspended in 60 mL lysis buffer (25 mM imidazole, 50 mM Tris-HCl, 300 mM NaCl, 1 mM phenylmethylsulfonyl fluoride (PMSF), pH 8). The bacterial suspensions were sonicated on an ice bath for 10 minutes with intervals of 10 s ON and 50 s OFF at an amplitude of 340 W using a Branson 550 Sonicator. Cell debris was removed by centrifugation at 15000 RPM for 30 min, and the lysate was collected and passed through a 0.22 µm syringe filter. The clarified lysates were then mixed with 1 ml of nickel-NTA resin (Qiagen), preequilibrated with lysis buffer, and incubated on a rotisserie overnight a 4°C. After incubation, the mixture was poured into a glass gravity flow chromatography column (Bio-Rad), and the resin was washed with 50 mL of lysis buffer, followed by elution of the bound protein with 6 ml elution buffer (500 mM imidazole, 50 mM Tris-HCl, 300 mM NaCl, pH 8). Purified proteins were dialyzed at 4°C in 10-kDa molecular weight cutoff (MWCO) dialysis cassettes (Thermo Scientific). Dialysis conditions were as follows: 1 L buffer 1 (100 mM imidazole, 50 mM Tris-HCl, 300 mM NaCl, pH 8) for 3 hours, 1 L buffer 2 (25 mM imidazole, 50 mM Tris-HCl, 300 mM NaCl, pH 8) for 3 hours, 1 L buffer 3 (50 mM Tris-HCl, 300 mM NaCl, pH 8) overnight, and 1 L buffer 4 (50 mM Tris-HCl, 300 mM NaCl, pH 8) for 3 hours. Protein concentration was measured using a bicinchoninic acid (BCA) kit (Thermo Scientific). Protein purity was confirmed by resolving the purified proteins using sodium dodecyl sulfate polyacrylamide gel electrophoresis (SDS-PAGE), followed by staining gels with Coomassie blue. Purified proteins were stored in aliquots at -80°C until use.

### RNA purification

*S. aureus* strains were struck onto fresh TSA plates and incubated overnight at 37°C. The next day, a single colony from the plate was inoculated into 3 mL of TSB in a 15 mL conical tube and incubated overnight at 37°C with shaking at 200 RPM. The following day, 250 μL of overnight culture was added to 25 mL of TSB + BCCAs in a 250 mL flask. Flasks were placed at 37°C with shaking at 200 RPM. For RNA sequencing, cultures were grown to late exponential phase (OD_600_ = 0.6) and for qPCR, strains were grown for 6 hours. 10 mL of each culture was removed and subsequently normalized to the OD_600_ of the strain with the lowest turbidity to ensure equivalent biomass. Cultures were centrifuged at 11000 RPM and washed twice with 15 mL PBS. Cell pellets were fixed in 15 mL of a 1:1 ethanol: acetone solution and stored at -80°C until RNA extraction. RNA was subsequently purified using a RNeasy Micro kit (Qiagen) with an additional DNase digestion using Turbo DNase (Thermo). Briefly, bacterial pellets were thawed on ice and centrifuged for 15 minutes at 4000 RPM at 4°C to remove the ethanol:acetone solution. The pellet was air-dried and was washed twice with 500 μL of 1x PBS, followed by suspension of the washed pellet in 500 μL RLT buffer containing 10 μL/mL of β-mercaptoethanol. Suspensions were transferred to RNase-free Lysing Matrix B tubes (MP Biomedicals). Tubes were placed in a Fast Prep-24 5G (MP Biomedicals) bead disruption system, followed by two sequential cell lysis steps at 5.0 speed for 20 s and 4.5 speed for 20 s. Samples were kept on ice for 5 minutes between the two lysis steps. After lysis, samples were centrifuged at 10000 RPM for 20 min, and the supernatant was removed. 500 μL of RLT buffer was added to supernatants and the samples were vortexed, followed by addition of 500 μL of 100% ethanol to precipitate the RNA, followed by addition to a Qiagen RNeasy spin column. All remaining steps were performed according to the manufacturer’s protocol. The RNA was eluted in 40 μL of DEPC-treated water. A second DNase digestion was conducted using 1 U Turbo DNase in solution (Invitrogen). The RNA was precipitated with 100% ethanol, added to an RNeasy spin column, washed 3 times in 450 μL wash buffer, and eluted in 50 μL of DEPC-treated water. The purified RNA was quantified via Nanodrop and RNA quality was assessed either by resolving the RNA on a 1.2% agarose/formaldehyde gel or by tape station analysis. RNA was stored at -80°C until cDNA synthesis or RNA sequencing.

### qRT-PCR and RNA sequencing

For qRT-PCR, cDNA was first synthesized from 5 ng of purified RNA using the GoScript reverse transcription system following the manufacturer’s protocol (Promega). Gene expression was assessed using SYBR Green (Bio-Rad) with the primer pairs listed in S2 Table on a QuantStudio 5 real-time PCR system. Fold change relative to housekeeping genes (*gyrB* or *sigA*) was calculated using the threshold cycle (2 − ΔΔCT) method. RNA sequencing was completed by SeqCoast Genomics LLC. Briefly, samples were prepared for RNA sequencing using an Illumina Stranded Total RNA Prep Ligation with Ribo-Zero Plus Microbiome and IDT For Illumina Unique Dual Indexes. Sequencing was performed on the Illumina NextSeq2000 platform using a 300-cycle flow cell kit to produce 2 x 150 bp paired reads. Read demultiplexing, read trimming, and run analytics were performed using DRAGEN v4.2.7, an on-board analysis software on the NextSeq2000. The quality of the sequencing data was first assessed using FastQC v0.12.0. Next, all reads were trimmed and quality filtered using BBDuk v39.33 employing Illumina’s adapters and the program’s default settings. To remove any potential contaminating reads, the data were subjected to another round of quality processing. This was performed by splitting the reads and removing the contaminating reads, using BBSplit v39.33 and the reference genome of *S. aureus* subsp. *aureus* LAC. Due to the lower support of *S. aureus* subsp. *aureus* LAC reference genome (NC_007793) in the downstream analysis tools and databases, the processed reads were instead aligned to the reference genome of *S. aureus* subsp. *aureus* TCH1516 using Bowtie 2 v2.5.4. SAMtools v1.22.1 were then employed to convert the output files of Bowtie 2, followed by the counting of the mapped reads to the genomic features using featureCounts (from the Subread package, v2.1.1). Finally, differential expression analysis was conducted employing DESeq2 (version 1.40.2). Specifically, count matrices and sample metadata were imported with the DESeqDataSetFromMatrix function and normalized using DESeq2’s median-of-ratios method. Afterwards, gene-wise dispersions were estimated, and a negative-binomial generalized linear model was fit to the counts using a design formula that included the condition factor. Statistical significance of coefficients was assessed using Wald test, and resulting p-values were adjusted for multiple testing by the Benjamini–Hochberg procedure to control the false discovery rate. Log2-fold changes were reported after effect-size shrinkage using the apeglm method, which provides a more stable and interpretable log-fold change estimate for ranking and visualization. Genes with adjusted p-value ≤ 0.05 were considered differentially expressed and consequently further analyzed. The data obtained from the differential expression analysis were further processed employing Pathview v1.48.0 for mapping and rendering the differentially expressed genes on relevant pathway graphs, clusterProfiler v4.16.0 for gene set enrichment analysis, and the individual genes manually parsed using Microsoft Excel (v2505). Normalized and mapped reads were visualized in Integrative Genomics Viewer (IGV).

### Pathway analysis

Functional pathway enrichment was carried out using approaches similar to those described in a prior study [107]. In summary, the set of differentially expressed genes was assigned to biological pathways using the free Database for Annotation, Visualization and Integrated Discovery (DAVID) provided by NIAID/NIH [108,109]. Gene identifiers were converted to NCTC8325 locus tags in the SAOUHSC ortholog format with the help of AureoWiki, an online resource for *S. aureus*. The DAVID analysis returned unbiased functional annotations, suggested pathway groupings, and the number of genes associated with each pathway.

### Growth curves

*S. aureus* strains were struck onto fresh TSA plates and incubated overnight at 37°C. The next day, a single colony from the plate was inoculated into 3 mL of TSB in a 15 mL conical tube and incubated overnight at 37°C with shaking at 200 RPM. Strains containing a Δ*lipL* mutation were grown with complete BCCAs overnight. The next day, strains were washed three times with 5 mL 1 x PBS and subcultured 1:100 (2 μL into 198 μL fresh TSB) in a flat-bottom 96-well plate (Costar) in technical triplicate. Strains were grown in TSB + BCCAs without the addition of sodium acetate. The plate was incubated in a Tecan Spark plate reader at 37°C with orbital shaking at 3 mm with a frequency of 180 RPM. Bacterial growth was monitored by measuring OD_600_ every hour for 24 hours.

### Metabolite quantification

*S. aureus* strains were struck onto fresh TSA plates and incubated overnight at 37°C. The next day, a single colony from the plate was inoculated into 3 mL of TSB in a 15 mL conical tube and incubated overnight at 37°C with shaking at 200 RPM. The following day, 250 μL of overnight culture was added to 25 mL of TSB + BCCAs without sodium acetate in a 250 mL flask. Flasks were placed at 37°C with shaking at 200 RPM. For glucose, lactate, and formate measurements, 120 μL of culture was removed every hour for six hours. For acetate measurements, 60 μL of culture was collected every three hours for 12 hours, with a final collection at 24 hours. Samples were collected in 1.5 mL Eppendorf tubes at each time point and were centrifuged at 13000 RPM for 5 minutes. The cellfree supernatant was stored at -20°C until assaying metabolite levels. Acetate, glucose, formate, and D- and L-lactate concentrations were determined using kits purchased from R-Biopharm, according to the manufacturer’s protocol with minor changes. Briefly, for all metabolites, 10 μL of each sample was incubated with 200 μL of reagent 1 for 3 minutes at room temperature in a flat-bottom 96-well plate (Costar) in technical triplicate. Following the primary incubation, 50 μL of reagent 2 was added to the reaction mixture and incubated for 15 minutes. A Tecan Spark plate reader was used to read the absorbance at 340 nm for each metabolite. Final calculations of metabolite concentrations were determined using a standard curve.

### Intracellular pyruvate quantification

*S. aureus* strains were struck onto fresh TSA plates and incubated overnight at 37°C. The next day, a single colony from the plate was inoculated into 3 mL of TSB in a 15 mL conical tube and incubated overnight at 37°C with shaking at 200 RPM. The following day, 250 μL of overnight culture was added to 25 mL of TSB + BCCAs without sodium acetate in a 250 mL flask and placed at 37°C with shaking at 200 RPM. At 3 hours, cultures were normalized to the lowest optical density and 1 mL of normalized culture was added to 1.5 mL Eppendorf tubes, centrifuged at 10,000 RPM for two minutes, and washed twice with 1 mL 1x PBS. The bacterial suspension was mixed with pyruvate assay buffer from a commercial Pyruvate Assay Kit (Abcam) and transferred to screw cap microcentrifuge tubes (Fisher Scientific), which were preloaded with 250 µL of 0.1 mm glass beads (Electron Microscopy Sciences). Tubes were placed in a Fast Prep-24 5G (MP Biomedicals) bead disruption system, followed by two sequential cell lysis steps at 5.0 speed for 20 s and 4.5 speed for 20 s. Samples were kept on ice for 5 minutes between the two lysis steps. After lysis, samples were centrifuged at 10000 RPM for 20 min, and 150 µL of the supernatant was collected and assayed according to the manufacturer’s protocol.

### Determination of protein lipoylation and densitometry

*S. aureus* strains were struck onto fresh TSA plates and incubated overnight at 37°C. The next day, a single colony from the plate was inoculated into 3 mL of TSB in a 15 mL conical tube and incubated overnight at 37°C with shaking at 200 RPM. Overnight cultures were diluted 1:100 in 10 mL fresh TSB in 50 mL conical tubes and incubated for 9 hours at 37°C with shaking at 200 RPM. Bacteria were centrifuged at 8000 RPM for 10 minutes, supernatants were removed, and cell pellets were resuspended in 1 mL 1x PBS. Bacterial suspensions were transferred to screw cap microcentrifuge tubes (Fisher Scientific), which were preloaded with 250 µL of 0.1 mm glass beads (Electron Microscopy Sciences). Tubes were placed in a Fast Prep-24 5G (MP Biomedicals) bead disruption system, followed by two sequential cell lysis steps at 5.0 speed for 20 s and at 4.5 speed for 20 s. Samples were kept on ice for 5 minutes between the two lysis steps. After lysis, samples were centrifuged at 11,000 RPM for 20 minutes, and 150 µL of the supernatant was collected in 1.5 mL centrifuge tubes containing 50 µL of 4X SDS sample buffer (0.2 M Tri-HCl, pH 6.8, 8% SDS, 5.5 M glycerol, 0.02 M EDTA, 0.6 M β-mercaptoethanol, and 6 mM Bromophenol blue). Samples were boiled for 10 minutes and stored at -20°C. Thawed cell lysates were loaded onto 15% polyacrylamide gels after normalizing to the original OD_600_ of the culture, followed by SDS-PAGE at 100 V for 2 hours. Resolved proteins were either stained with Coomassie blue or were transferred to 0.2 μm Immobilon polyvinylidene difluoride (PVDF) membranes (Millipore Sigma) at 20 V for 90 min. After transfer, membranes were incubated for 1 hour in Tris-buffered saline + 0.1% TWEEN 20 (Amresco) (TBST) supplemented with 5% bovine serum albumin (BSA) (GoldBio) and human IgG (Sigma) (1:2,000). Rabbit polyclonal α-lipoic acid antibody (Calbiochem) was added to the membranes at a 1:7,500 dilution followed by incubation overnight at 4°C. The following day, the membrane was washed three times for 10 minutes each in ∼20 mL of TBST. Alkaline phosphatase (AP)-conjugated goat anti-rabbit IgG (H + L) (Invitrogen) was then added at a 1:5000 dilution in 5% BSA in TBST for 1 hour followed by three 10 minutes washes in ∼20 mL of TBST. Membranes were developed with 5-bromo-4-chloro-3-indoyl-phosphate/nitro blue tetrazolium color development substrate (GoldBio). Densitometry analysis was performed using the ImageJ software on four independent experiments to determine the lipoylation of each band relative to the control band set at a value of 1.

### Bacterial Adenylate Cyclase Two-Hybrid (BACTH) system

*S. aureus* *pta* and *lipL* genes were amplified (S2 Table) and cloned into plasmids pKT25 and pUT18c to generate translational fusions with the T25 or T18 domains of the *Bordetella pertussis* adenylate cyclase [51]. To clone the *lipL* gene into BACTH vectors, Sma1 and Pst1 restriction endonucleases were used for digestion and T4 DNA ligase for ligation. To clone the *pta* gene into BACTH vectors, Sma1 and Xba1 restriction endonucleases were used for digestion and T4 DNA ligase for ligation. The resulting plasmids were transformed into *E. coli* DH5α and plated on LB agar containing kanamycin (50 μg/mL) or ampicillin (100 μg/mL). Transformants containing the ligated amplicon were verified by PCR. The recombinant plasmids were purified from DH5α *E. coli* and then co-transformed into *E. coli* strain BTH101 and plated on LB agar containing both kanamycin (50 μg/mL) and ampicillin (100 μg/mL). To assay for protein-protein interactions, single colonies were inoculated into 3 mL LB in 15 mL conical tubes containing both antibiotics overnight. The following day, strains were diluted 1:100 and grown for 6 hours at 30°C with shaking at 200 RPM. Upon reaching late-exponential phase (~6 hours), 10 μL culture was spotted on LB agar containing IPTG (0.5 mM), ampicillin (100 μg/mL), kanamycin (50 μg/mL), and X-Gal (100 μg/mL). Plates were incubated for 24 hours at 30°C to allow for bacterial growth. An interaction was defined by the presence of blue color relative to positive and negative controls. The negative control, empty BACTH plasmids, yield colorless colonies. The positive control was BTH101 co-transformed with pKT25-zip and pUT18C-zip, plasmids expressing leucine zipper - adenylate cyclase fusions that dimerize to yield blue colonies.

β-galactosidase activity was quantified based on previous methods [110]. Briefly, single colonies were inoculated in 3 mL LB in 15 mL conical tubes containing both antibiotics overnight. The next day, the cultures were diluted 1:100 in 3 mL LB and grown for 6 hours with IPTG (0.5 mM), ampicillin (100 μg/mL) and kanamycin (50 μg/mL) with shaking at 200 RPM at 30°C until reaching an OD_600_ ~ 1.0. Strains were OD normalized and 100 μL of each sample was added to a 96-well plate (Costar) in triplicate. The cultures were centrifuged at 4000 RPM, and the bacterial pellets were resuspended in 80 μL of Z-buffer (60 mM Na₂HPO₄·7H₂O, 40 mM NaH₂PO₄·H₂O, 10 mM KCl, 1 mM MgSO₄, 50 mM β-mercaptoethanol, pH 7). The resuspended cells were then permeabilized with 10 μL of 0.1% SDS and 10 μL of chloroform. Plates were centrifuged at 4000 RPM, the supernatant was transferred to a new 96-well plate, mixed with 20 μL ONPG (4 mg/mL), and the reaction was allowed to proceed for 15–30 min. Reactions were stopped by adding 30 μL 1 M Na_2_CO_3_ and absorbance was measured at 420 nm. The negative control, BTH101 carrying the empty pKT25 and pUT18c plasmids, and the positive control, BTH101 containing pKT25-zip and pUT18c-zip plasmids, are described above. β-galactosidase activity is reflected relative to the negative and positive controls, with the negative control assigned an activity value of 0 in arbitrary units and positive control assigned an activity of 1000 arbitrary units.

### Microscale Thermophoresis (MST)

Experiments were performed as described previously with minor modifications [111]. Briefly, pure recombinant Pta was labeled with a NanoTemper 2nd Generation Red N-hydroxysuccinimide (NHS) Dye (Nanotemper Technologies). For protein labeling, Pta was first buffer exchanged into buffer M (20 mM MES, 100 mM NaCl, 10% glycerol, pH 7.5) to remove incompatible buffer components. 10 µM Pta was mixed with 3X excess of the Red NHS dye (300 µM) and incubated in the dark for 30 minutes for the dye to react with the protein. The protein-dye mixture was passed through a gel filtration column (Column B, Nanotemper Technologies) to remove unreacted and excess dye. For a 1:1 protein-dye conjugate, the degree of label (DOL) of the protein was determined using the formula: A650/195,000/M/cm × concentration of labeled protein. A650; absorbance at 650 nm, and 195,000/M/cm is the molar absorbance of the Red NHS dye. A 1:1 protein dye ratio occurred at a DOL value between 0.6 and 1. After labeling, protein samples were aliquoted into 10µL volumes, flash frozen using liquid nitrogen, and stored at -80^o^C until used. For microscale thermophoresis experiments, the unlabeled protein partner was serially diluted in low-binding tubes with buffer M supplemented with 1 mM BME and 0.05% Tween 20, pH 7.5, and titrated against 20 nM of the labeled proteins. The protein mixtures were loaded into standard Monolith NT.115 capillary tubes, and thermophoresis was determined using the following parameters: 20% excitation power and high MST Power at 25°C.

Thermophoresis results were analyzed using the PALMIST [112] and GUSSI [112] analysis pipeline. Briefly, data from the MST software (Mo. Control v1.6.1) were imported into the PALMIST software and a preset T-jump (TJ) was applied to the data using a 1:1 binding model with a 95% confidence interval. Datasets were examined for kinetic effects to ensure that thermophoresis achieved equilibrium. After data analysis, GUSSI was used for figure rendering. The top panel in Fig 4B indicates the normalized fluorescence data points, the middle panel represents the binding curve, and the lower panel represents the residuals (a plot between the fitted line and the data). Error bars indicate the standard deviation of four independent replicates.

### Murine infection models

*S. aureus* strains were struck onto fresh TSA plates and incubated overnight at 37°C with shaking at 200 RPM. The next day, a single colony from the plate was inoculated into 3 mL of TSB in a 15 mL conical tube and incubated overnight at 37°C with shaking at 200 RPM. The overnight cultures were diluted 1:100 in 3 mL fresh TSB and grown for 3 h at 37°C with shaking at 200 RPM. Bacteria were pelleted by centrifugation at 3900 RPM at 4°C for 5 minutes. The cells were washed three times with 5 mL of 1X PBS. Bacterial cell suspensions were normalized to an OD_600_ of ~0.32 (~1 × 10^8^ CFU/mL), and serial dilutions were plated onto TSA plates to enumerate CFU and ensure accuracy of inocula. Six-week-old female Swiss Webster mice (Envigo) were anesthetized with ketamine/xylazine (100/10 mg/kg) via intraperitoneal injection. For systemic infections, 100 μL of OD-normalized *S. aureus* in PBS (~1.0 × 10^7^ CFU) was injected directly into the bloodstream via the retro-orbital sinus. Mice were monitored daily. At 96 h postinfection, mice were euthanized, and the kidneys were recovered, homogenized, serially diluted, dilutions were plated onto tryptic soy agar followed by incubation at 37°C overnight to enumerate CFU. For skin infections, bacterial suspensions (~1.0 × 10^7^ CFU) were injected intradermally into anesthetized mice on each side of a shaved flank region. Mice were monitored daily. At 72 h postinfection, mice were euthanized, and abscesses were excised, homogenized, and plated to enumerate CFU.

### Statistical analysis

For *in vitro* growth assays, western blots, and metabolite quantifications, data shown are representative of at least three independent experiments conducted in triplicate. qRT-PCR experiments included two independent biological replicates conducted in technical triplicate with all data shown. All animal studies include combined data from at least two independent experiments. Statistical analyses were conducted using GraphPad Prism version 10. Comparisons among three or more groups were conducted using either one-way ANOVA followed by Tukey’s post hoc test or Kruskal–Wallis test followed by Dunn’s post-test, whereas differences between two groups were analyzed with the Mann–Whitney test. A *p*-value below 0.05 was considered statistically significant. Statistical significance is indicated as follows: *, *p* < 0.05; **, *p* < 0.01; ***, *p* < 0.001; ****, *p* < 0.0001.

## Supporting information

S1 Fig Growth of WT, Δ*pta*, Δ*lipL*, Δ*pta* + *pta*, and Δ*lipL* + *lipL* strains in TSB and TSB + BCCAs [10 mM isobutyric acid (IB), 9 mM 2-methylbutyric acid (2MB), 9 mM isovaleric acid (IV) + 10 mM sodium acetate (NaAc)] containing 0 mM glucose (A) or 3.5 mM glucose (B). (C) Quantification of glucose, acetate, lactate and formate from WT, Δ*pta*, Δ*lipL*, Δ*pta* + *pta*, and Δ*lipL* + *lipL* culture supernatants over time in TSB with 0 mM glucose. (D) Quantification of glucose, acetate, lactate and formate from WT, Δ*pta*, Δ*lipL*, Δ*pta* + *pta*, and Δ*lipL* + *lipL* culture supernatants over time in TSB with 3.5 mM glucose. Each metabolite quantification assay is representative of three independent experiments, with each timepoint measured in technical triplicate. Errors bars indicate standard deviation from the mean.
(TIF)

S2 Fig (A) qRT-PCR analysis of RNA extracted from WT, Δ*pta*, and Δ*lipL* strains at 3 and 6 hours of growth. qRT-PCR experiments were conducted with two biological replicates in technical triplicate. (B) Top - schematic of the *cid* operon. Bottom - normalized and mapped transcript reads across the *cid* operon in WT and Δ*lipL* strains. (B) Bacterial adenylate cyclase two-hybrid (BACTH) assay was used to test interactions between N-terminal-tagged Pta with LipL and C-terminal-tagged Pta with E2-PDH. Graph displays β-galactosidase activity (Miller Units) from *E. coli* BTH101 co-transformed with pUT18-*pta* and pKT25-*lipL*, pUT18C-*pta* and pKT25-*pdhC*, pUT18C-zip and pKT25-zip (positive control – PC) or pUT18C and pKT25 vectors (negative control – NC). ****, p < 0.0001 by one-way ANOVA with Tukey’s post hoc test. Cultures of *E. coli* BTH101strains co-transformed with the aforementioned plasmid combinations were also spotted on LB agar plates with X-Gal as an indicator. Data are representative of at least three independent experiments. (D) AlphaFold3 prediction of the interaction between Pta (blue) and E2-PDH (green). pTM (0.48) and an ipTM (0.2) are displayed graphically to the right of the model.
(TIF)

S1 Table Strains used in this study. (PDF)

S2 Table List of Oligonucleotides used in this study. (PDF)

S3 Table Significant differentially expressed genes (Δ*lipL* mutant compared to WT *S. aureus*). (XLSX)

S4 Table NIH DAVID pathway analysis. (XLSX)
