# Correction: *Staphylococcus aureus* adapts to the host nutritional environment by coordinating the activity of central metabolic enzymes

**DOI:** 10.1371/journal.ppat.1014398

**Published:** 2026-07-06

**Authors:** The PLOS Pathogens Staff

## Notice of Republication

This article was republished on June 11, 2026, to correct errors in the author Francis Alonzo III’s name. The publisher apologizes for the errors. Please download this article again to view the correct version. The originally published, uncorrected article and the republished, corrected articles are provided here for reference.

## Supporting information

S1 File Originally published, uncorrected article. (PDF)

S2 File Republished, corrected article. (PDF)
