# Supplementary material for: Staphylococcus aureus adapts to the host nutritional environment by coordinating the activity of central metabolic enzymes
Source: PLoS Pathog. 2026 May 4;22(5):e1014183. doi: 10.1371/journal.ppat.1014183 (PMC13155669; doi:10.1371/journal.ppat.1014183)
Supplement: S1 Table — (PDF) [file ppat.1014183.s003.pdf]

Supplemental Table 1. Strains used in this study.

Strain	Genotype	Designation	Reference
USA300-LAC	WT *S. aureus* USA300 (AH-1263) parent strain, plasmid cured.	LAC (WT)	(1)
FA-S1776	LAC with an in-frame deletion of *pta*	Δ*pta*	This study
FA-S1795	LAC Δ*pta* transduced with pJC1111-P_{HELP}-*pta*	Δ*pta* + *pta*	This study
FA-S1176	LAC with an in-frame deletion of *lipL*	Δ*lipL*	(2)
FA-S1691	LAC Δ*lipL* transduced with pJC1111- P_{HELP}-*lipL*	Δ*lipL* + *lipL*	(3)
FA-S1765	LAC with an in-frame deletion of *pta-lipL*	Δ*pta* Δ*lipL*	This study
FA-S1779	LAC Δ*pta* Δ*lipL* transduced with pJC1111- P_{HELP}-*pta*	Δ*pta* Δ*lipL* + *pta*	This study
FA-S1781	LAC Δ*pta* Δ*lipL* transduced with pJC1111- P_{HELP}-*lipL*	Δ*pta* Δ*lipL* + *lipL*	This study
FA-S3223	LAC with an in-frame deletion of *cidC*	Δ*cidC*	This study
FA-S3460	LAC Δ*cidC* transduced with pJC1111- P_{HELP}-*cidC*	Δ*cidC* + *cidC*	This study
FA-S3468	LAC Δ*pta* with an in-frame deletion of *cidC*	Δ*pta* Δ*cidC*	This study
FA-S3464	LAC Δ*pta* Δ*cidC* transduced with pJC1111- P_{HELP}-*pta*	Δ*pta* Δ*cidC* + *pta*	This study
FA-S3462	LAC Δ*pta* Δ*cidC* transduced with pJC1111- P_{HELP}-*cidC*	Δ*pta* Δ*cidC* + *cidC*	This study
FA-S1008	LAC with gene replacement of *agrBDCA* with tetracycline resistance cassette	Δ*agr*	(4)
FA-S3540	LAC Δ*pta* Δ*cidC* with gene replacement of *agrBDCA* with tetracycline resistance cassette	Δ*agr* Δ*pta* Δ*cidC*	This study
RN4220	Restriction deficient *S. aureus* strain for plasmid passage	RN4220	(5)
RN9011	RN4220 with pRN7203 plasmid expressing the SaPI-1 integrase	RN9011	(6)
DH5α	*E. coli* strain for propagating recombinant pIMAY, pJC1111, pUT18C, and pKT25 plasmids	DH5α	NEB, Cat # C2989K
IMO8B	*E. coli* strain for propagating recombinant pIMAY	IMO8B	(7)
BTH101	Adenylate cyclase deficient (*cya*) *E. coli* reporter strain for BACTH assay	BTH101	(8)
FA-S3572	*E. coli* BTH101 expressing pUT18C-*pta* and pKT25-*lipL*	BTH101 + pUT18C-*pta* + pKT25-*lipL*	This study
FA-S3573	*E. coli* BTH101 expressing pUT18C-zip and pKT25-zip (BACTH assay positive control)	BTH101 + pUT18C-zip + pKT25-zip	This study
FA-S3574	*E. coli* BTH101 expressing pUT18C (empty) and pKT25 (empty) (BACTH assay negative control)	BTH101 + pUT18C + pKT25	This study
FA-S3645	*E. coli* BTH101 expressing pUT18-*pta* and pKT25-*lipL*	BTH101 + pUT18N-*pta* + pKT25-*lipL*	This study
FA-S3646	*E. coli* BTH101 expressing pUT18C-*pta* and pKT25-*pdhC*	BTH101 + pUT18C-*pta* + pKT25-*pdhC*	This study
T7 Express *lysY/I^q^*	*E. coli* strain for expressing recombinant Pta-6xHis and LipL-6xHis	*lysY/I*^{q}	NEB, Cat # C3013I
FA-S1469	T7 Express *E. coli* strain expressing recombinant Pta-6xHis	*lysY/I*^{q} + p21a-*pta-6xHis*	This study
FA-S3562	T7 Express *E. coli* strain expressing recombinant LipL-6xHis	*lysY/I*^{q} + p21a-*lipL-6xHis*	This study

References

1. Boles BR, Thoendel M, Roth AJ, Horswill AR. Identification of Genes Involved in Polysaccharide-Independent Staphylococcus aureus Biofilm Formation. PLoS One. 2010 Apr 14;5(4):e10146.

2. Zorzoli A, Grayczyk JP, Alonzo F. Staphylococcus aureus Tissue Infection During Sepsis Is Supported by Differential Use of Bacterial or Host-Derived Lipoic Acid. Peschel A, editor. PLoS Pathog. 2016 Oct 4;12(10):e1005933.

3. Teoh WP, Resko ZJ, Flury S, Alonzo F. Dynamic Relay of Protein-Bound Lipoic Acid in Staphylococcus aureus. Journal of Bacteriology. 2019 Oct 21;201(22):e00446-19.

4. Cosgriff CJ, White CR, Teoh WP, Grayczyk JP, Alonzo F. Control of Staphylococcus aureus Quorum Sensing by a Membrane-Embedded Peptidase. Infection and Immunity. 2019 Apr 23;87(5):10.1128/iai.00019-19.

5. Fairweather N, Kennedy S, Foster TJ, Kehoe M, Dougan G. Expression of a cloned Staphylococcus aureus alpha-hemolysin determinant in Bacillus subtilis and Staphylococcus aureus. Infect Immun. 1983 Sep;41(3):1112–7.

6. Chen J, Yoong P, Ram G, Torres VJ, Novick RP. Single-copy vectors for integration at the SaPI1 attachment site for Staphylococcus aureus. Plasmid. 2014 Nov;76:1–7.

7. Monk IR, Tree JJ, Howden BP, Stinear TP, Foster TJ. Complete Bypass of Restriction Systems for Major Staphylococcus aureus Lineages. mBio. 2015 May 26;6(3):10.1128/mbio.00308-15.

8. Battesti A, Bouveret E. The bacterial two-hybrid system based on adenylate cyclase reconstitution in Escherichia coli. Methods. 2012 Dec;58(4):325–34.
