# Supplementary material for: Staphylococcus aureus adapts to the host nutritional environment by coordinating the activity of central metabolic enzymes
Source: PLoS Pathog. 2026 May 4;22(5):e1014183. doi: 10.1371/journal.ppat.1014183 (PMC13155669; doi:10.1371/journal.ppat.1014183)
Supplement: S2 Table — (PDF) [file ppat.1014183.s004.pdf]

Supplemental Table 2. List of oligonucleotides used in this study.

Name	Sequence	Reference
ptaSOE1-KpnI	ATATGGTACCAGAAACAAATATAATCAGAATG	This study
dptaSOE2	CTCGCTAAATCCATCGAATGTCCTCCTGTAATA	This study
dptaSOE3	TATTACAGGAGGACATTGCATGGATTTAGCGAG	This study
ptaSOE4-SacI	ATATGAGCTCCTTCACCTTTTAAAGCATGT	This study
pta-0571SOE1-KpnI	ATATGGTACCAGAAACAAATATAATCAGAATG	This study
pta-0571 SOE2	GGCTGTTAAATATTTAACTATTGAATGTCCTCCTGTAATA	This study
pta-0571 SOE3	TATTACAGGAGGACATTCAATAGTTAAATATTTAACAGCC	This study
pta-0571SOE4-SacI	ATATGAGCTCCCTTTTCTTGTCATGTCAAT	This study
ptaSOE1-Pst	ATATCTGCAGATCCCATTATGCTTTGGCA	(1)
ptaSOE2	ATACATTTAATAAATCAGCCATGGGTTTCACTCTCCTTCTA	(1)
ptaSOE3	TAGAAGGAGAGTGAAACCCATGGCTGATTTATTAAATGTAT	(1)
ptsSOE4-SacI	ATATGAGCTCCCTTTTCTTGTCATGTCAAT	(1)
UniCompSOE1-PstI	ATATCTGCAGATCCCATTATGCTTTGGCA	(2)
LipLCompSOE2	ATTTACTCGCTAAATCCATGGGTTTCACTCTCCTTCTA	(2)
LipLCompSOE3	TAGAAGGAGAGTGAAACCCATGGATTTAGCGAGTAAAT	(2)
LipLCompSOE4-SalI	ATAGTCGACCTATTGCATTTGATCTATCATT	(2)
cidC Primer 1 FWD-Kpn1	CCCGGTACCAGCCGGCAGTATTGTTG	This study
cidC Primer 2 REV	TGTGATAACCTTTAAATCAGATTACTAATAGCCTCCCTT	This study
cidC Primer 3 FWD	AAGGGAGGCTATTAGTAATCTGATTTAAAGGTTATCACA	This study
cidC Primer 4 REV-Sac1	CCCGAGCTCTAAACCAAATGGAATTAATG	This study
Phelp Fwd-Sal1	CCCGTCGACATCCCATTATGCTTTGGC	This study
Phelp CidC Rev	TAATAGCCTCCCTTTCTG-GGGTTTCACTCTCCTTC	This study
CidC Fwd	GAAGGAGAGTGAAACCC-CAGAAAGGGAGGCTATTA	This study
CidC Rev-Sac1	CCCGAGCTCCAATTGTGATAACCTTTAAAT	This study
Pta BACTH F-XbaI	CCCTCTAGAGCTGATTTATTAAATGTATTAA	This study
Pta BACTH R-Xma1	CCCCCCGGGTTGTAAGGCTTGCGCTG	This study
LipL BACTH F-Pst1	CCCCTGCAGGATTTAGCGAGTAAATATTTT	This study
LipL BACTH R-Xma1	CCCCCCGGGTTGCATTTGATCTATCATTTT	This study
PDH-F-BamHI	CCCGGATCCGCATTTGAATTTAGATTACC	This study
PDH-R-EcoRI	CCCGAATTCCCCCTCCATTAATAATAATT	This study
21a-Pta-F-Nde1	TATACATATGGCTGATTTATTAAATGTATTAA	This study
21a-Pta-R-Xho1	TATACTCGAGTTGTAAGGCTTGCGCTG	This study
21a-LipL-F-Nde1	CCCCATATGGATTTAGCGAGTAAATATTTT	This study
21a-LipL R-Xho1	CCCCTCGAGTTGCATTTGATCTATCATTTT	This study
gyrB F (qPCR)	CTAGAACGCAGGCGATTTTACC	This study
gyrB R (qPCR)	CTTTCGCTAGATCAAAGTCGCC	This study
sigA F (qPCR)	AATTGCACGAGTGATTGCTTGT	This study
sigA R (qPCR)	CTTGCAGAAGCGAACTTACGTT	This study
pta-lipL F (qPCR)	TTATCACGTGGCTGCTCAATTG	This study
pta-lipL R (qPCR)	ATATCTTTGCCCACACTTTCGC	This study
pta F (qPCR)	CGACGTAAAGGTAAAGCGACTG	This study
pta R (qPCR)	TGATTTGTAAAGCTGGACGCAC	This study
lipL F (qPCR)	TGGTTCAGAACGTGCATTGATG	This study
lipL R (qPCR)	CCGCCAAGATCTTTGATTGCAT	This study
cidC F (qPCR)	GATCCTAATGCTGCGCCATTAC	This study
cidC R (qPCR)	CGTTTTGCTGCCACTGAAATTG	This study
sucB F (qPCR)	TTGCAGATGCTTGTTGTGTTGT	This study
sucB R (qPCR)	TTGCAGATGCTTGTTGTGTTGT	This study
pdhC F (qPCR)	GCACCTGCAGCAGTAACATTAG	This study
pdhC R (qPCR)	CCTTGTTCAGCTGCGATTTCTT	This study

References

1. Zorzoli A, Grayczyk JP, Alonzo F. Staphylococcus aureus Tissue Infection During Sepsis Is Supported by Differential Use of Bacterial or Host-Derived Lipoic Acid. Peschel A, editor. PLoS Pathog. 2016 Oct 4;12(10):e1005933.

2. Teoh WP, Resko ZJ, Flury S, Alonzo F. Dynamic Relay of Protein-Bound Lipoic Acid in Staphylococcus aureus. Journal of Bacteriology. 2019 Oct 21;201(22):e00446-19.
