# Supplementary material for: Correction: Staphylococcus aureus adapts to the host nutritional environment by coordinating the activity of central metabolic enzymes
Source: PLoS Pathog. 2026 Jul 6;22(7):e1014398. doi: 10.1371/journal.ppat.1014398 (PMC13336151; doi:10.1371/journal.ppat.1014398)
Supplement: S2 File — (PDF) [file ppat.1014398.s002.pdf]

PLOS Pathogens

RESEARCH ARTICLE

Staphylococcus aureus adapts to the host nutritional environment by coordinating the activity of central metabolic enzymes

Reginald A. Woods[1,2], Iván C. Acosta[2], Zachary J. Resko[3], Charles Agbavor[4], Luka Svet[5], Manaar Yousef[2], Wei Ping Teoh[3], Victor J. Torres[5], Laty A. Cahoon[4], Francis Alonzo III[2,6]*

1 Department of Pharmaceutical Sciences, University of Illinois at Chicago – College of Pharmacy, Chicago, Illinois, United States of America, **2** Department of Microbiology and Immunology, University of Illinois at Chicago – College of Medicine, Chicago, Illinois, United States of America, **3** Department of Microbiology and Immunology, Loyola University Chicago - Stritch School of Medicine, Maywood, Illinois, United States of America, **4** Department of Biological Sciences, University of Pittsburgh, Pittsburgh, Pennsylvania, United States of America, **5** Department of Host-Microbe Interactions, St Jude Children's Research Hospital, Memphis, Tennessee, United States of America, **6** Lead contact

* falonzo@uic.edu

OPEN ACCESS

Citation: Woods RA, Acosta IC, Resko ZJ, Agbavor C, Svet L, Yousef M, et al. (2026) *Staphylococcus aureus* adapts to the host nutritional environment by coordinating the activity of central metabolic enzymes. PLoS Pathog 22(5): e1014183. https://doi.org/10.1371/journal.ppat.1014183

Editor: Rachel M McLoughlin, Trinity College Dublin, IRELAND

Received: December 16, 2025

Accepted: April 20, 2026

Published: May 4, 2026

Copyright: © 2026 Woods et al. This is an open access article distributed under the terms of the Creative Commons Attribution License, which permits unrestricted use, distribution, and reproduction in any medium, provided the original author and source are credited.

Data availability statement: The data that support the findings of this study are publicly available from Mendeley Data with the identifier: doi: 10.17632/f3vc62ng3y.1.

Funding: This work was supported by National Institutes of Health grants R01AI120994

Abstract

The nutritional demands imposed by disparate infection sites represent a significant barrier to bacterial survival. Yet, the proclivity of pathogens such as *Staphylococcus aureus* to cause disease at nearly all host sites implies significant metabolic flexibility to promote infection. *S. aureus* catabolizes glucose in several ways, including the phosphotransacetylase (Pta) – acetate kinase (AckA) pathway. The Pta-AckA pathway uses acetyl-CoA derived from the major glycolytic end-product, pyruvate, to rapidly generate ATP, producing acetate as a byproduct. Yet, flux through Pta-AckA necessitates coordinating glycolytic activity with the production of acetyl-CoA and its delivery to Pta-AckA. The generation of acetyl-CoA occurs through the pyruvate dehydrogenase (PDH) complex, an enzyme that requires attachment of the metabolic cofactor, lipoic acid, for its function. Thus, delivery of lipoic acid to enzyme complexes has the potential to serve as a determinant of metabolic adaptation. Here, we provide evidence for a functional link between the lipoic acid transfer enzyme, LipL, and Pta. We demonstrate *pta* and *lipL* are transcriptionally coupled and that Pta and LipL directly interact, with potential to direct metabolic flux through Pta-AckA. Both Δ*pta* and Δ*lipL* mutants are defective for acetate production with evidence for alternative fates for pyruvate that depend on blockade upstream or downstream of the pyruvate node. The functional pairing of Pta and LipL is required for optimal skin infection, whereas Pta and LipL have separable functions in bloodstream infection. Furthermore, we found that a complete block to acetate production leads to significant attenuation *in vivo*, cementing a direct role for acetogenesis in infection. Together, our results establish a mechanism by which *S. aureus* regulates metabolite flux by

(F.A.), F30AI183565 (R.A.W.), R35GM159731 (L.A.C.), and the American Lebanese Syrian Associated Charities, a fundraising and awareness organization for St. Jude Children's Research Hospital (V.J.T.). The funders had no role in the study design, data collection and analysis, decision to publish, or preparation of the manuscript.

Competing interests: The authors have declared that no competing interests exist.

coupling enzymes in linked metabolic pathways to promote energy balance and survival during infection.

Author summary

Staphylococcus aureus causes infections within nearly all host tissues. Several virulence traits contribute to its survival at diverse sites, including the ability to produce myriad toxins, adhesins, and immunomodulatory enzymes. *S. aureus* also has remarkable metabolic plasticity, allowing it to overcome host-imposed nutrient restriction. In this work, we establish a genetic and functional coupling that links two metabolic pathways involved in *S. aureus* central carbon metabolism. Our work suggests that the coordinated expression and activity of an enzyme required for the delivery of the metabolic cofactor, lipoic acid, and an enzyme involved in acetogenesis, facilitates optimal metabolite flux and promotes *S. aureus* survival during infection. Furthermore, our studies support a direct role for acetate production in *S. aureus* survival during infection. Altogether, this work introduces a new facet of regulatory control over metabolic plasticity in *S. aureus* with important implications for pathogenesis.

Introduction

The host nutritional landscape can dramatically affect the ability of bacterial pathogens to establish infection [1–6]. The adaptation of microbes to host nutrient deprivation is crucial for pathogens that cause infection in a wide range of host tissues [7]. *Staphylococcus aureus* is a Gram-positive pathogen that can infect nearly all host tissues and causes significant morbidity and mortality globally [8,9]. To successfully establish infection, this bacterium not only uses tissue-specific defenses that block immune clearance, but also harnesses the host environment to optimize metabolism and energy expenditures [3,4,6,10,11]. *S. aureus* adapts to the host nutritional milieu by employing a complex network of transcriptional regulators that fine-tune metabolic gene expression to promote survival and growth [4,12]. The ability of *S. aureus* to optimize metabolic efficiency during infection extends beyond transcriptional regulation. Several bioinformatic and functional studies indicate that the directionality and efficiency of metabolite production are enhanced by high-affinity or transient protein-protein interactions and post-translational modifications [13–16], highlighting critical post-translational checkpoints in metabolic adaptation. As a pathogen that evolved closely with humans, *S. aureus* also adapts by engaging in piracy of host nutrients during infection [1,2,5,17,18]. Indeed, *S. aureus* can use host lipids, carbohydrates, metals, and proteins to augment metabolite flux or bypass energetically demanding biological processes [2–4,19–21]. Work from our lab and others suggests that the ability of *S. aureus* to establish infection in diverse tissues is due in part to adaptations that permit scavenging of key nutrients or redirecting of metabolism to promote survival [1,2,5,17,18,22].

One molecule with low bioavailability in the host is the metabolic cofactor lipoic acid, on account of its synthesis as a protein-bound precursor and its rapid clearance after dietary consumption [23–25]. Lipoic acid is an organosulfur derivative of the medium-chain fatty acid, octanoic acid, that is required for the activation of α-ketoacid dehydrogenase complexes. Its redox-sensitive dithiolane ring aids in shuttling substrates through each complex [25]. The lipoic acid-dependent enzymes of *S. aureus* include pyruvate dehydrogenase (PDH), 2-oxoglutarate dehydrogenase (OGDH), and branched-chain 2-oxoacid dehydrogenase (BCODH) complexes, as well as the glycine cleavage system (GCS) [1,26]. *S. aureus* synthesizes lipoic acid *de novo* and scavenges it from host environments to promote survival [1]. The use of both pathways during infection is exemplified by evidence that shows lipoic acid scavenging is required for survival in some host tissues (kidneys), but not others (heart) [1]. Central to lipoic acid acquisition is the amidotransferase, LipL, which harnesses lipoic acid bound to the H subunits of the glycine cleavage system (GcvH) or the GcvH homolog, GcvH-L, for delivery to conserved lysine residues on the E2 subunits of each α-ketoacid dehydrogenase complex (E2-PDH, E2-OGDH, and E2-BCODH) [1,26]. Lipoic acid attachment to these enzyme complexes represents an essential post-translational modification, as LipL-deficient strains of *S. aureus* have compromised growth *in vitro* and are highly attenuated during bloodstream infection [5,26]. Altogether, these studies suggest that dynamic delivery of lipoic acid to metabolic enzyme complexes is likely required to facilitate metabolic plasticity and pathway adaptations needed to promote bacterial survival during infection.

LipL-dependent lipoylation of the E2 subunits of PDH and OGDH is critical for glycolysis and the tricarboxylic acid (TCA) cycle, respectively [26]. PDH generates acetyl-CoA from pyruvate upon exit from glycolysis, and OGDH generates succinyl-CoA from α-ketoglutarate in the TCA cycle [1,26]. The acetyl-CoA generated by PDH has several fates depending on the status of the cell and the nutrients available for use. These include the potential to enter fermentative pathways, fatty acid biosynthesis, TCA cycle, and nucleotide biosynthesis pathways [27–29]. During carbon overflow *in vitro, S. aureus* primarily ferments glucose by shuttling acetyl-CoA into the phosphotransacetylase (Pta) - acetate kinase (AckA) pathway to rapidly synthesize ATP [28]. Pta catalyzes the generation of acetyl-phosphate from acetyl-CoA, which is used in a substrate-level phosphorylation reaction by AckA to generate acetate and ATP [27,28]. The Pta-AckA pathway promotes rapid growth *in vitro* during overflow metabolism when glucose and oxygen are abundant [27]. Extensive prior work established that the Pta-AckA pathway is vital for energy production and metabolic homeostasis during overflow metabolism in *S. aureus* [28,30]. Though *pta* is commonly positioned near, or co-transcribed with, *ackA* in many bacterial pathogens, we previously determined that in *S. aureus,* the start codon of *lipL* is located two nucleotides downstream of the *pta* stop codon [26]. The potential genetic linkage between *pta* and *lipL* and the dependency on efficient LipL-dependent lipoyl transfer for PDH activity highlights the relevance of LipL to central metabolism and suggests a potential functional link to acetate fermentation pathways.

As a facultative anaerobe, *S. aureus* can replicate in both high and low oxygen environments [3,31]. Past studies indicate that Pta-AckA activity increases during growth in low oxygen to support NAD+ generation and fermentative processes [27,32]. This extends the role of Pta beyond overflow metabolism and implies relevance of the pathway to conditions that occur during host infection (e.g., low oxygen). Inactivation of the Pta-AckA pathway shifts *S. aureus* metabolism away from acetate fermentation, leading to increased carbon flux through glycolysis and the TCA cycle [28]. Pathway inactivation also leads to early bacterial cell death in some *S. aureus* strains due to the induction of the *cid* genes [28]. This is thought to be due to the imposed metabolic block at pyruvate and the cellular requirement to combat intracellular pyruvate accumulation [28]. The *cid* locus comprises three genes, *cidABC*, which play a vital role in stationary phase cell death, autolysis, and biofilm formation [33–36]. CidA is a functional holin that supports endolysin-induced cell lysis, and CidC is a pyruvate:menaquinone oxidoreductase that converts pyruvate directly to acetate [37,38]. Although its mechanism of action is not fully understood, CidB is thought to act in tandem with CidA to regulate cell lysis and is necessary for full activation of CidC [39]. Work from Thomas *et al.* found that inactivation of CidC decreased the rate of stationary phase cell death, which disrupts *S. aureus* biofilm development and biofilm-related disease outcomes [36]. Despite the connection between

Pta-AckA and CidC as compensatory pathways to accommodate carbon flow, we do not yet have a complete picture of how Pta, CidC, and LipL interface to promote metabolic adaptability during infection. To date, no studies have assessed the individual versus combined contributions of Pta and CidC during *S. aureus* infection with planktonic cells.

The prevailing evidence thus far suggests there are contributions from several metabolic pathways, including glycolysis, as well as lipoic acid biosynthesis and salvage, to *S. aureus* adaptation to host tissues [1,40–42]. The unusual positioning of *pta* and *lipL* genes in the *S. aureus* genome may further link lipoic acid transfer and acetogenesis in ways that dictate the fate of major glycolytic metabolites. In this work, we investigated the convergence of lipoic acid relay and Pta-AckA pathways and determined the relative contribution of each to pathogenesis. We show that *pta* and *lipL* share a distinct genetic organization in *Staphylococci* that diverges from pathogenic firmicutes and Gram-negative pathogens. *pta* and *lipL* are co-transcribed, and the two encoded proteins (Pta and LipL) directly interact. This genetic and functional linkage promotes infection in the skin, whereas separable functions for Pta and LipL are required for systemic infection. Furthermore, we verified the established block at the pyruvate node in the absence of Pta and determined that a similar block occurs in the absence of LipL, leading to a commensurate increase in *cidC* transcripts and lactate production during aerobic growth. Finally, we found that bacterial acetate production is required for survival in both skin and systemic infection. Altogether, these studies provide new insight into the factors governing the metabolic plasticity of *S. aureus* during infection with potential applicability to other pathogens.

Results

pta and *lipL* are genetically linked in *Staphylococci*

S. aureus preferentially uses glycolysis to metabolize glucose (Fig 1A) [4,19,43]. The lipoic acid-dependent PDH complex serves as a major exit point from the glycolytic pathway, where the decarboxylation of pyruvate leads to the generation of acetyl-CoA. During periods of carbon overflow *in vitro*, acetyl-CoA is predominantly shuttled into the Pta-AckA pathway to generate ATP and acetate via substrate-level phosphorylation (Fig 1A), in part because carbon catabolite repression suppresses TCA cycle gene expression [44,45]. Following glucose consumption, acetate is reassimilated via acetyl-CoA synthase (Acs), for use in the TCA cycle (Fig 1A). Central to glycolysis and TCA cycle function is LipL-dependent lipoylation of the E2 subunits of PDH and OGDH (Fig 1A). In a prior study, we determined that the *lipL* (SAUSA300_0571) open reading frame begins two nucleotides downstream from the stop codon of *pta* (SAUSA300_0570), implying a gene pairing that links lipoyl transfer activity with the Pta-AckA pathway [26]. Considering *pta* and *ackA* are genetically linked in classically studied microorganisms such as *Escherichia coli*, we wondered if this unusual *pta*-*lipL* linkage observed in *S. aureus* was widespread. We conducted a comparative synteny analysis of the *pta* gene environment. We surveyed representative Gram-negative or Gram-positive bacterial pathogens and *Mycobacterium tuberculosis*, as well as commensals *Lactococcus lactis* subs. *cremoris, Bacillus subtilis*, and *E. coli,* and found that most Gram-negative pathogens surveyed were likely to have *ackA* and *pta* positioned in an operon, like *E. coli* (Fig 1B). By contrast, analysis of *M. tuberculosis* and Gram-positive pathogens showed that *pta* and *lipL* were in close genetic proximity for some Gram-positives (*Bacillus anthracis* and *S. aureus*), whereas others were positioned at greater than 70kb nucleotide distances (~70kb for *Streptococcus pyogenes,* ~ 2500kb for *Listeria monocytogenes*, and ~300kb for *Enterococcus faecalis*) (Fig 1C). *S. aureus* was the only species with *pta* and *lipL* genetically linked in a putative operon, potentially implying a unique genetic arrangement for *Staphylococci* (Fig 1C). Indeed, coagulase-positive and negative *Staphylococci* all have the same *pta*-*lipL* organization, indicating it is a conserved feature of the genus (Fig 1D).

To establish that *S. aureus* co-transcribes *pta* and *lipL*, we conducted RNA sequencing of WT *S. aureus* and a Δ*lipL* mutant grown to late exponential phase. The read analysis indicated that *pta* and *lipL* transcripts consist of a single mRNA encoding both genes (Fig 1E). The deletion of *lipL* led to the generation of a truncated transcript but did not negatively impact *pta* expression (Fig 1E). To validate the RNAseq results, we measured transcript levels of *pta*, *lipL* and the junction between the *pta* and *lipL* genes via qRT-PCR. Transcript levels were similar between the *pta*, *lipL,* and the junction in WT

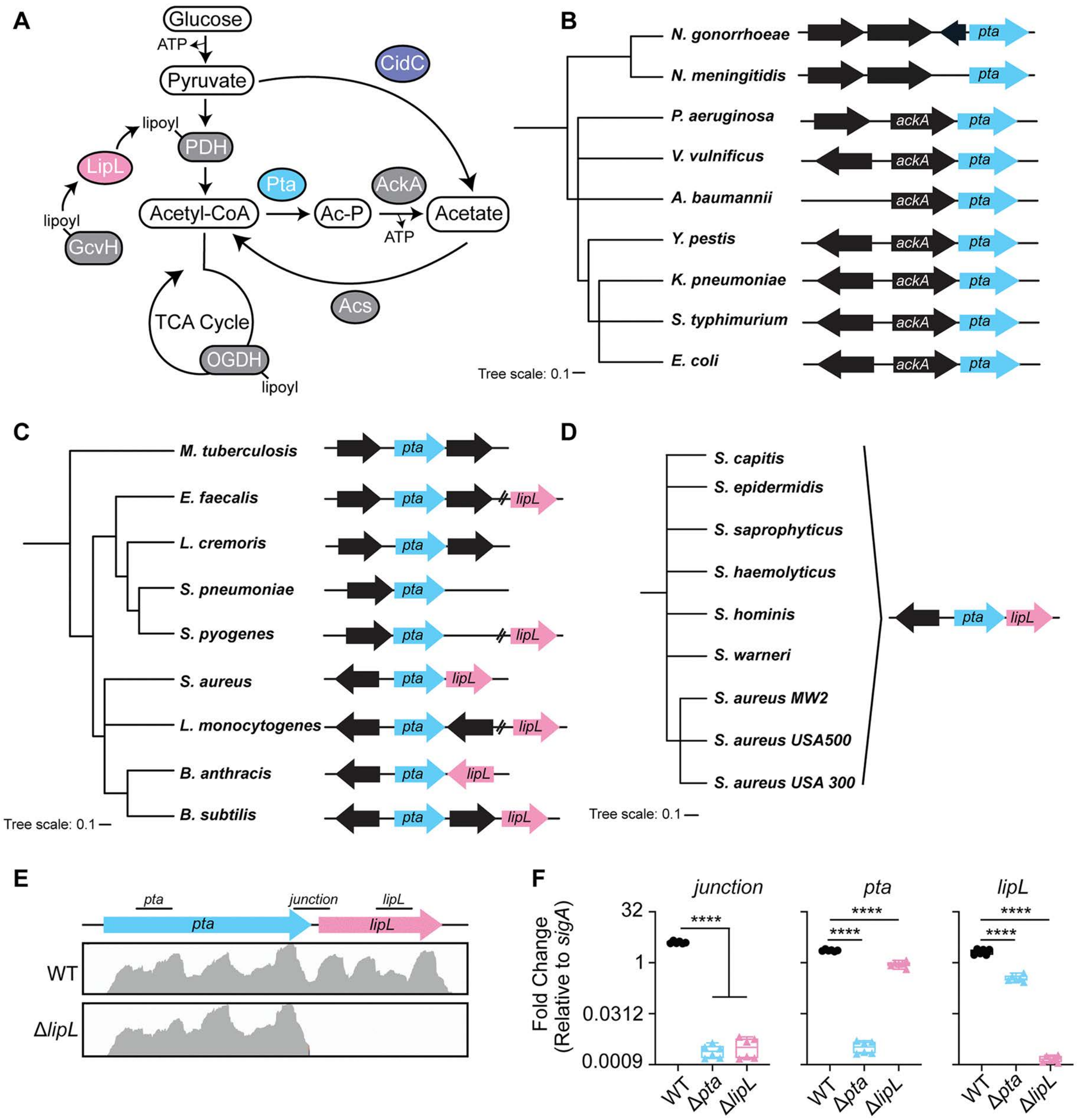

Fig 1. *pta* and *lipL* are genetically linked in *Staphylococci.* **(A)** Model of acetate fermentation in *S. aureus*. Glucose is converted to pyruvate, the primary end-product of glycolysis. Pyruvate has several fates, one of which is its oxidative decarboxylation by pyruvate dehydrogenase (PDH) to generate Acetyl-CoA. Pyruvate can also be directly decarboxylated (CidC) to produce acetate. Acetyl-CoA can enter the tricarboxylic acid (TCA) cycle or is used by the phosphotransacetylase (Pta) to generate Acetyl-phosphate (Ac-P), the substrate for acetate kinase (AckA). The activity of AckA generates Acetate and ATP. PDH activity is regulated by lipoic acid (lipoyl) attachment, which is mediated by delivery of lipoic acid from the H subunit of the glycine cleavage system (GcvH) by the amidotransferase, LipL. **(B-D)** Synteny analysis of *pta* and *lipL* genes. **(B)** Phylogenetic tree of Gram-negatives, including *pta* synteny analysis adjacent to the relevant microorganism. **(C)** Phylogenetic tree of Gram-positives and *M. tuberculosis*, including *pta* synteny analysis adjacent to the respective microorganism. **(D)** Phylogenetic tree of *Staphylococci* including *pta* synteny analysis. **(E)** Normalized and mapped transcript reads across the *pta*-*lipL* region in WT and a Δ*lipL* mutant. **(F)** qRT-PCR analysis of RNA extracted from WT, Δ*pta,* and Δ*lipL* strains at 6 hours of growth. ****, $p < 0.0001$ by one-way ANOVA with Tukey's post hoc test. qRT-PCR experiments were conducted with two independent biological replicates in technical triplicate. Boxplots indicate the median and quartiles. Whiskers indicate the range.

https://doi.org/10.1371/journal.ppat.1014183.g001

S. aureus (Fig 1F). Junctional amplicons were not detected from the Δ*pta* and Δ*lipL* mutants (Fig 1F). Altogether, these data indicate that the genetic link between *pta* and *lipL* is conserved in Staphylococci and the genes are co-transcribed.

Loss of either *pta* or *lipL* compromises acetate production

Given the evidence for co-transcription of *pta* and *lipL* in *S. aureus* and the role of Pta in overflow metabolism, we sought to determine how these genes contribute to acetate production via Pta-AckA and metabolic flux in general. We generated in-frame single and double deletions of *pta* and *lipL* in the methicillin-resistant *Staphylococcus aureus* (MRSA) strain LAC [46,47]. We complemented *pta* and *lipL* in single copy in the chromosome with constitutive expression [48], and subsequently determined if the loss of *pta* or *lipL* resulted in growth defects or shifts in metabolite production. The Δ*pta* mutant had a marginal delay in exit from logarithmic phase growth in tryptic soy broth (TSB) compared to WT (Fig 2A). A Δ*lipL* mutant, on the other hand, had compromised growth kinetics, including dramatically reduced growth in TSB, consistent with previous work from our group (Fig 2A) [1]. A significant component of the Δ*lipL* mutant growth defect in TSB is due to loss of LipL-dependent lipoylation of the BCODH complex, which is essential for branched-chain fatty acid synthesis [5]. Indeed, growth of Δ*lipL* mutant was substantially restored in TSB supplemented with branched-chain carboxylic acids (BCCAs) (isobutyric acid, 2-methylbutyric acid, and isovaleric acid) that bypass the requirement for BCODH or with use of the *lipL* complement strain [1,26] (Fig 2A). To test for defects in lipoylation of E2 subunits of enzyme complexes, we conducted immunoblots on whole cell lysates of the Δ*pta* and Δ*lipL* mutants and their complement strains grown in TSB + BCCAs. Consistent with prior results, the Δ*lipL* mutant had defective lipoylation compared to WT [26] (Fig 2B), whereas the Δ*pta* mutant had evidence for increased lipoylation on E2 subunits (Fig 2B). Thus, while lipoylation itself is not a direct effect of Pta, the degree of lipoylation on E2 subunits is increased in its absence (see below). These data indicate that the effects of *pta* and *lipL* on growth kinetics and lipoylation in broth are separable and largely driven by LipL-dependent effects on BCODH activity.

We next assessed glycolytic flux over time by measuring glucose and acetate levels in media containing 14 mM glucose under aerobic conditions to promote carbon overflow metabolism [28,49]. In addition to acetate, we measured lactate and formate as alternative pyruvate fermentation products in *S. aureus* [31,32,50]. As previously reported, glucose was rapidly consumed within 6 hours of growth along with a commensurate increase in acetate that peaked at 6 hours and was reassimilated in stationary phase (Fig 2C) [27,28]. The Δ*pta* mutant consumed all the glucose in the media by 6 hours with no differences compared to the WT strain (Fig 2C). Peak acetate levels from the Δ*pta* mutant were ~13 mM, a ~ 57% reduction compared to WT (Fig 2C). There were no differences in lactate or formate levels produced by the Δ*pta* mutant compared to WT (Fig 2C). Conversely, the Δ*lipL* mutant had a delay in the glucose consumption rate relative to WT (Fig 2D). Peak acetate production from the Δ*lipL* mutant was around 8 mM, a ~ 70% reduction compared to WT (Fig 2D). There was also a commensurate increase in lactate production by the Δ*lipL* mutant, which peaked at ~8 mM at 6 hours (Fig 2D). Growth and metabolite quantification assays using glucose-free media or low glucose supplementation (3.5 mM) established that acetate production requires LipL and that levels depend on the amount of glucose added. Compensatory increases in lactate production by a Δ*lipL* mutant are only observed when glucose is provided as a carbon source (14 mM and 3.5 mM) (S1 Fig and Fig 2D). Together, these data suggest that both Pta and LipL are required for optimal acetate fermentation during overflow metabolism and under conditions where glucose is limiting. While deletion of either *pta* or *lipL* leads to compensatory acetate production, presumably by other acetogenic pathways*,* lactate fermentation is only induced upon deletion of *lipL* in the presence of glucose, potentially to offset a pyruvate bottleneck during overflow that is associated with inactivation of PDH.

LipL and Pta coordinate PDH activity and acetate production

pta and *lipL* are co-transcribed (Fig 1). The deletion of either gene alone shifts acetate production and metabolic flux either downstream (Δ*pta*) or upstream (Δ*lipL*) of PDH (Figs 1A and 2). We hypothesized that the tandem expression of *pta-lipL* might promote coordination of pyruvate decarboxylation with the delivery of acetyl-CoA to Pta-AckA to improve metabolic

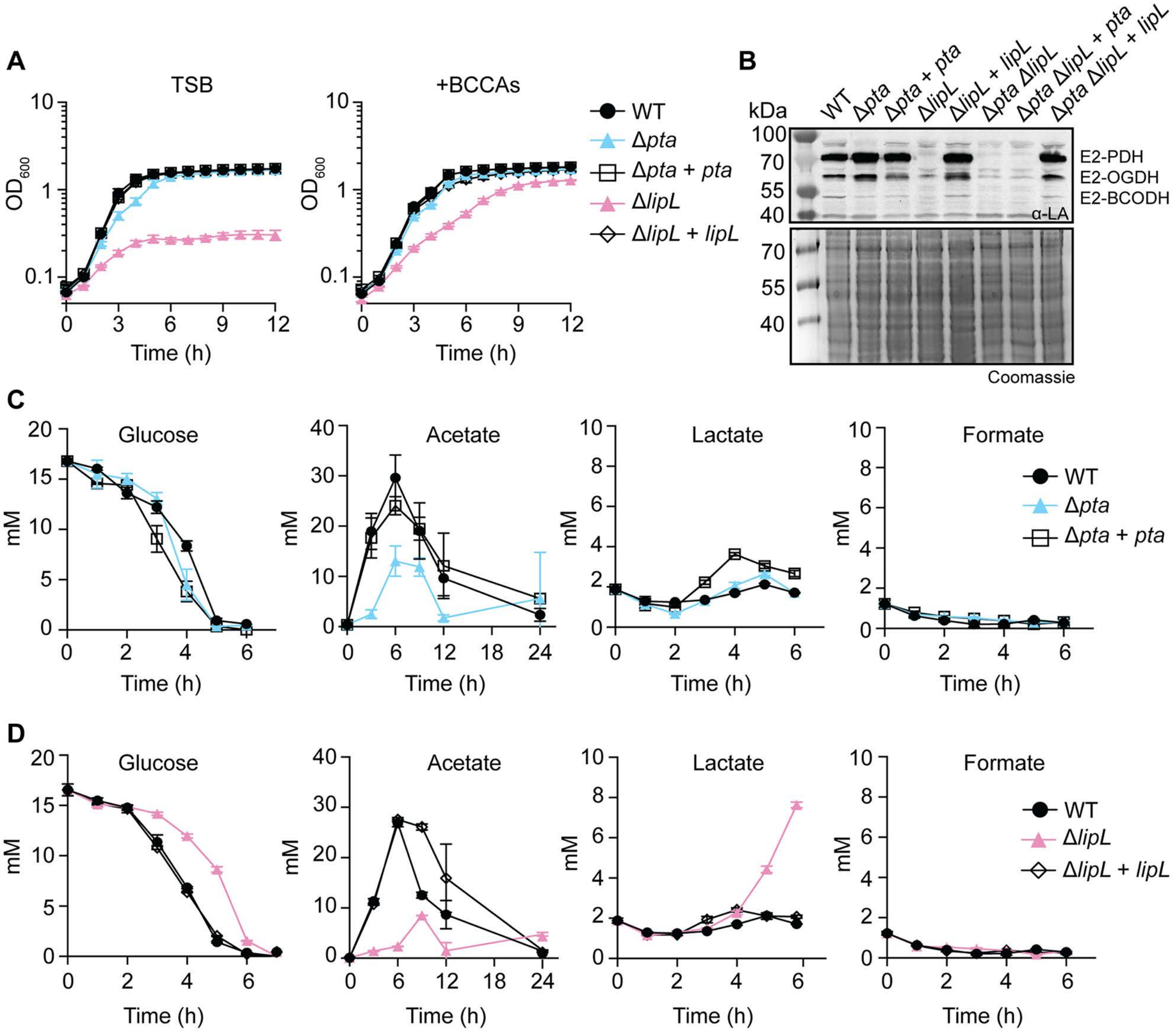

Fig 2. Loss of either *pta* or *lipL* compromises acetate production. (A) Growth of WT, Δ*pta,* Δ*lipL,* Δ*pta* + *pta,* and Δ*lipL* + *lipL* strains in TSB and TSB + BCCAs [10 mM isobutyric acid (IB), 9 mM 2-methylbutyric acid (2MB), 9 mM isovaleric acid (IV) + 10 mM sodium acetate (NaAc)] containing 14 mM glucose. **(B)** α-lipoic acid immunoblots of whole cell lysates from the indicated strains after 9-hours of growth in TSB medium. The presented blot and Coomassie-stained gel are representative of at least three independent experiments. **(C)** Quantification of glucose, acetate, lactate and formate from WT, Δ*pta,* Δ*pta* + *pta* culture supernatants over time in TSB with 14 mM glucose. **(D)** Quantification of glucose, acetate, lactate and formate from WT, Δ*lipL,* Δ*lipL* + *lipL* culture supernatants over time in TSB with 14 mM glucose and BCCA supplementation. Each metabolite quantification assay is representative of three independent experiments, with each timepoint measured in technical triplicate. Errors bars indicate standard deviation from the mean. Some lines on growth curve graphs (Δ*pta* + *pta,* and Δ*lipL* + *lipL*) are obscured because they overlap.

https://doi.org/10.1371/journal.ppat.1014183.g002

efficiency. To test this, we assessed the effect of loss of the entire *pta*-*lipL* locus on growth and metabolite production. The Δ*pta* Δ*lipL* mutant had severely compromised growth in TSB, which was largely complemented when TSB was supplemented with BCCAs or when using a Δ*pta* Δ*lipL* + *lipL* complement strain (Fig 3A). The Δ*pta* Δ*lipL* + *pta* strain did not restore growth in TSB without BCCAs (Fig 3A). Immunoblots on whole cell lysates of the Δ*pta* Δ*lipL* double mutant and complement strains revealed that only those strains lacking *lipL* had defective lipoylation profiles compared to WT (Fig 2B).

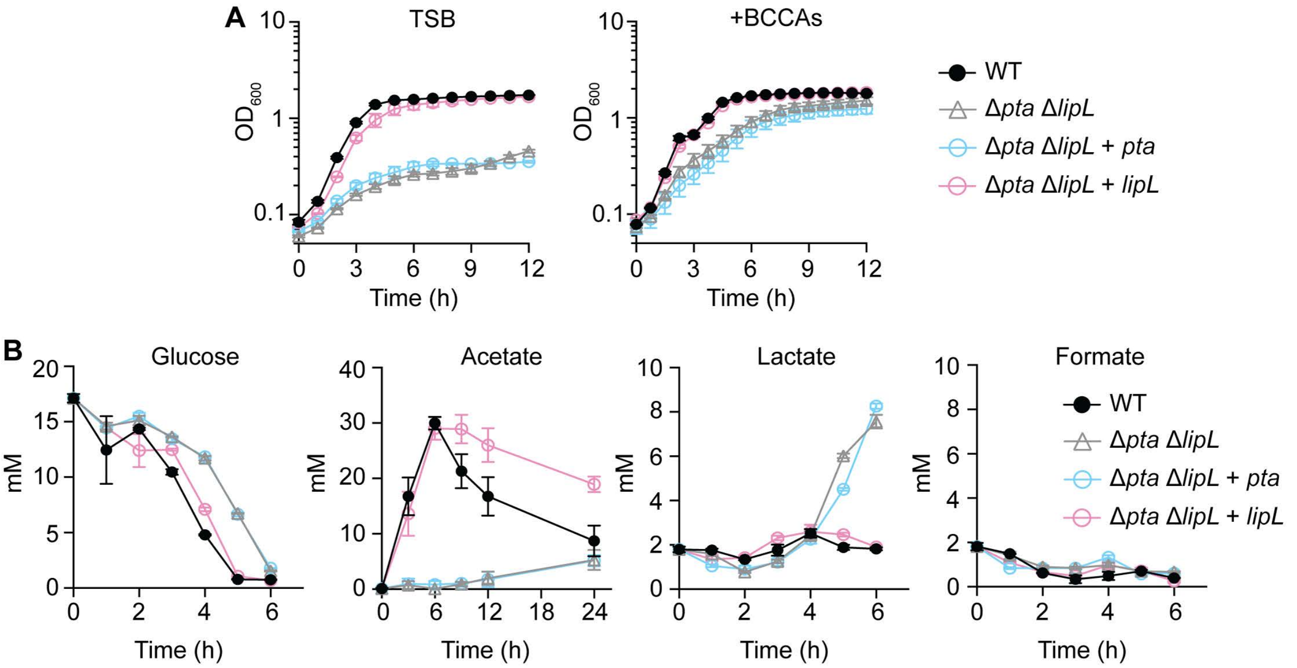

Fig 3. Pta and LipL coordinate PDH activity and acetate production. (A) Growth of WT, Δ*pta* Δ*lipL,* Δ*pta* Δ*lipL* + *pta,* and Δ*pta* Δ*lipL* + *lipL* strains in TSB and TSB + BCCAs [10 mM isobutyric acid (IB), 9 mM 2-methylbutyric acid (2MB), 9 mM isovaleric acid (IV) + 10 mM sodium acetate (NaAc)] containing 14 mM glucose. **(B)** Quantification of glucose, acetate, lactate, and formate from WT, Δ*pta* Δ*lipL,* Δ*pta* Δ*lipL* + *pta,* and Δ*pta* Δ*lipL* + *lipL* culture supernatants over time in TSB with 14 mM glucose and BCCA supplementation. Each metabolite quantification assay is representative of three independent experiments, with each timepoint measured in technical triplicate. Error bars indicate standard deviation from the mean.

https://doi.org/10.1371/journal.ppat.1014183.g003

The WT and Δ*pta* Δ*lipL* + *lipL* strains consumed all glucose by 6 hours, whereas the Δ*pta* Δ*lipL* double mutant and Δ*pta* Δ*lipL* + *pta* had delays in glucose consumption (Fig 3B). Notably, the Δ*pta* Δ*lipL* double mutant did not produce additional acetate (Fig 3B). The Δ*pta* Δ*lipL* + *pta* strain mirrored this phenotype and acetate levels did not accumulate beyond ~5 mM, a ~ 83% reduction compared to peak acetate production in the WT and Δ*pta* Δ*lipL* + *lipL* strains (Fig 3B). Intriguingly, the Δ*pta* Δ*lipL* + *lipL* strain had delayed acetate reassimilation. Furthermore, compensatory lactate production by the Δ*pta* Δ*lipL* double mutant and Δ*pta* Δ*lipL* + *pta* strains was consistent with a redirection of pyruvate toward lactate fermentation (Fig 3B). Taken together, these data confirm that defects in growth associated with the Δ*pta* Δ*lipL* mutant are due to activities of LipL that are independent of Pta-AckA and flux through PDH. Furthermore, loss of both *pta* and *lipL* halts acetate production entirely, redirecting pyruvate towards alternative fates, such as lactate fermentation. Constitutive expression of *lipL* favors alternative modes of acetate production that do not depend on Pta-AckA.

Pta and LipL proteins directly interact

Prior functional and computational studies suggest that metabolic flux can be enhanced by protein-protein interactions and protein complex formation [13–15]. Considering our demonstration of the coordinated expression of *pta* and *lipL* and the necessity of both for optimal acetate production, we hypothesized there might be a direct interaction between Pta and LipL that could facilitate acetate fermentation. We first used AlphaFold3 to predict the likelihood of a direct interaction between Pta and LipL. The AlphaFold3 model predicted a potential interaction between Pta and LipL, with a template modeling of 0.76 and an interface predicted template modeling (ipTM) score of 0.61 (Fig 4A). Considering the ipTM score of the predicted interaction falls at the likelihood threshold for a potential interaction, we opted to validate this

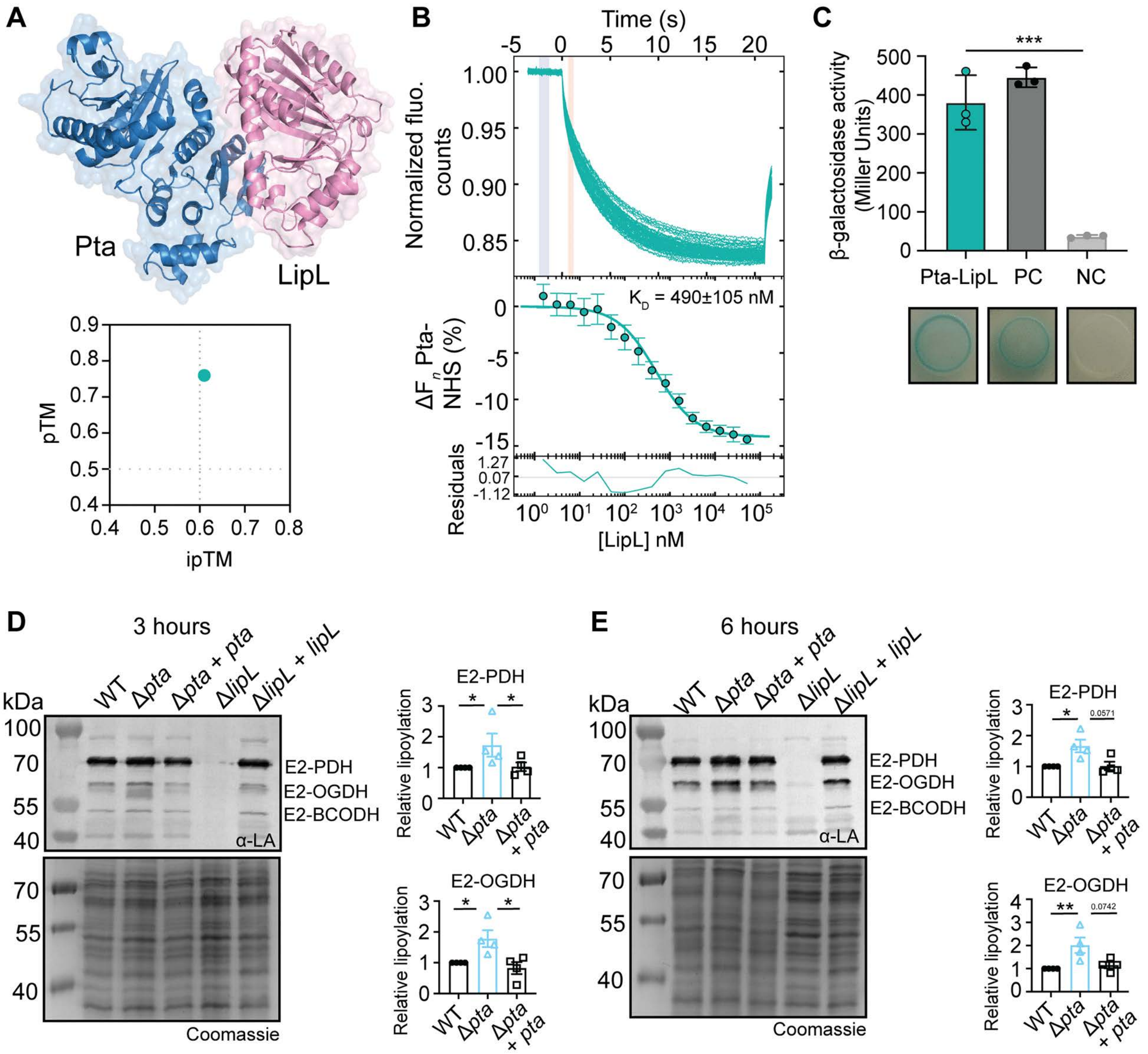

Fig 4. Pta and LipL proteins directly interact. (A) AlphaFold3 prediction of the interaction between Pta (blue) and LipL (pink). pTM (0.76) and an ipTM (0.61) are displayed graphically below the model. **(B)** Microscale thermogram of Pta-NHS titrated against the recombinant LipL. The top panel shows the thermophoretic time-traces of four independent experiments, and the blue and pink areas represent time spans used to obtain the fluorescence cold (F_c) and hot (F_h) regions, respectively. The middle panel shows the binding curve with the line of best fit using the 1:1 binding model with an error surface projection confidence of 95%. The residuals between the data and fit are shown in the bottom panel. *Kd* is presented as the mean ± standard deviation of four independent replicates. **(C)** Bacterial adenylate cyclase two-hybrid (BACTH) assay was used to assay an interaction between Pta and LipL. Graph displays β-galactosidase activity (Miller Units) from *E. coli* BTH101 co-transformed with pUT18C-*pta* and pKT25-*lipL*, pUT18C-zip and pKT25-zip (positive control – PC) or pUT18C and pKT25 vectors (negative control – NC). ***, $p < 0.001$ by one-way ANOVA with Tukey's post hoc test. Cultures of *E. coli* BTH101strains co-transformed with the aforementioned plasmid combinations were also spotted on LB agar plates with X-Gal as an indicator. Data are representative of at least three independent experiments. **(D-E)** α-lipoic acid immunoblots of whole cell lysates from WT, Δ*pta,* and Δ*pta* + *pta* strains after 3 and 6-hours of growth in TSB medium. Densitometric quantification of E2-PDH and E2-OGDH bands in Δ*pta* and Δ*pta* + *pta* strains relative to WT bands. *, $p < 0.05$; **, $p < 0.01$ by Kruskal-Wallis test with Dunn's post hoc analysis. The presented blots and Coomassie-stained gels are representative of four independent experiments. The mean and standard error of the mean are shown.

https://doi.org/10.1371/journal.ppat.1014183.g004

prediction by expressing and purifying Pta (Pta-6xHis) and LipL (LipL-6xHis) and conducting a biophysical assessment of a direct interaction using microscale thermophoresis (MST). Recombinant Pta was labeled with the amine-reactive dye, N-hydroxysuccinimide (NHS) and titrated against unlabeled LipL (Fig 4B). MST determined that Pta interacts with LipL in solution with moderate affinity (*Kd* of 490 +/- 105 nM) (Fig 4B). To determine if the proteins interact *in vivo*, we cloned the coding sequences of both *pta* and *lipL* into the adenylate cyclase-based Bacterial two-hybrid (BACTH) system [51,52]. Pta and LipL interacted strongly when expressed in *E. coli*, achieving similar levels of β-galactosidase activity as the leucine zipper positive control (Fig 4C). In considering the possibility that an interaction between Pta and LipL might improve efficiency of metabolic flux through PDH to Pta-AckA, we reasoned that loss of Pta would enhance the demand for LipL-mediated delivery of lipoic acid to E2 proteins of PDH and OGDH as a compensatory measure to drive carbon flux. Indeed, a Δ*pta* mutant had increased lipoylation on E2-PDH and E2-OGDH at 3 and 6 hours compared with WT and the Δ*pta* + *pta* strain (Fig 4D and 4E). This increase in lipoylation was not associated with an increase in *pdhC* (E2-PDH) or *sucB* (E2-OGDH) transcripts at 3 or 6 hours (S2A Fig), suggesting increased delivery of lipoic acid to E2-PDH and E2-OGDH by LipL. Collectively, these results indicate Pta and LipL directly interact *in vitro* and *in vivo*. Furthermore, the loss of Pta results in increased delivery of lipoic acid to E2-PDH and E2-OGDH as a compensatory measure.

The coordinated activity of Pta and LipL is important during infection of the skin, whereas additional functions of LipL promote systemic infection

Previous work from our group found that LipL is required for optimal *S. aureus* infection in both a murine sepsis model and a murine skin and soft tissue infection model [26]. Follow up studies determined that *S. aureus* is mostly refractory to lipoic acid deficiency in murine skin but not kidneys on account of its ability to bypass LipL-dependent branched-chain fatty acid synthesis by incorporating host unsaturated fatty acids [5]. These results do not preclude the possibility that the coordinated activity of Pta and LipL that drives acetate fermentation might contribute to infection in one or both infection models. To test this, we infected mice intravenously or intradermally with WT, Δ*pta*, Δ*pta* + *pta*, Δ*lipL*, Δ*lipL* + *lipL*, Δ*pta* Δ*lipL,* Δ*pta* Δ*lipL* + *pta*, and Δ*pta* Δ*lipL* + *lipL* strains. At 96 hours post intravenous infection, kidneys from Δ*pta* mutant-infected animals had ~100-fold fewer CFUs compared to WT (Fig 5A). By contrast, the kidneys from Δ*lipL* mutant-infected mice had ~ 10000-fold fewer CFUs (Fig 5A). The kidneys from Δ*pta* Δ*lipL,* and Δ*pta* Δ*lipL* + *pta* infected mice phenocopied Δ*lipL* mutant-infected mice with ~10000-fold fewer CFUs (Fig 5A). Systemic infection with Δ*pta* + *pta, ΔlipL* + *lipL, and* Δ*pta* Δ*lipL* + *lipL* complement strains restored CFUs in the kidney to nearly WT levels (Fig 5A). At 72 hours post intradermal infection, abscesses from mice infected either with Δ*pta*, Δ*lipL*, and Δ*pta* Δ*lipL* mutants phenocopied one another with ~10-fold fewer CFUs compared to WT (Fig 5B). Infection with the Δ*pta* + *pta* and Δ*lipL* + *lipL* complement strains restored abscess CFUs to nearly WT levels (Fig 5B). Infection with either Δ*pta* Δ*lipL* + *pta* or Δ*pta* Δ*lipL* + *lipL* strains did not restore CFU to WT levels (Fig 5B). Considering the identical attenuation for Δ*pta*, Δ*lipL*, and Δ*pta* Δ*lipL*, Δ*pta* Δ*lipL* + *pta* or Δ*pta* Δ*lipL* + *lipL* strains during infection of the skin, we conclude that the coordinated activity between Pta and LipL is required for *S. aureus* survival at this site. In contrast, during systemic infection, the contributions of Pta and LipL to virulence are separable and dominated by independent roles for LipL in promoting branched fatty acid synthesis, as we have previously published [5].

Loss of *lipL* induces significant transcriptional changes in *S. aureus*

Our data suggest that a block to exit from glycolytic metabolism at PDH redirects metabolic flux on account of pyruvate accumulation in the cell. To test this, we quantified intracellular pyruvate levels from WT, Δ*pta*, Δ*pta* + *pta*, Δ*lipL*, Δ*lipL* + *lipL*, Δ*pta* Δ*lipL,* Δ*pta* Δ*lipL* + *pta*, and Δ*pta* Δ*lipL* + *lipL* strains at 3 hours. We found that all strains harboring a Δ*lipL* mutation had approximately twice the amount of intracellular pyruvate compared to WT (~745 pmol for a Δ*lipL* mutant versus ~363 pmol for WT) (Fig 6A). The redirection of pyruvate metabolism is further substantiated by increases in lactate and Pta-AckA - independent generation of acetate (Figs 2D and 3B). Given the importance of LipL in activating the PDH, OGDH, and BCODH complexes [1,26], we hypothesized that, in addition to the shifts in glucose metabolism at the PDH node,

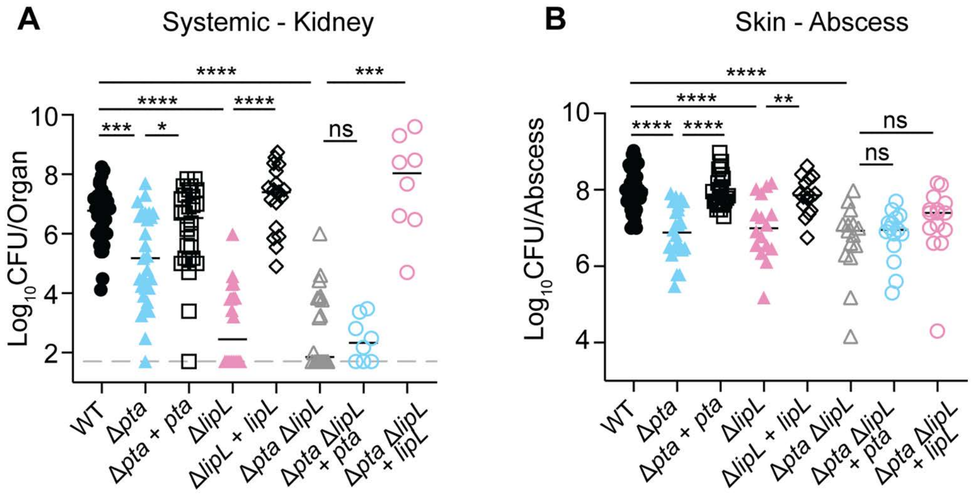

Fig 5. The coordinated activity of Pta and LipL is important during infection of the skin, whereas additional functions of LipL promote systemic infection. **(A)** Bacterial burden in the kidneys of mice 96 hours after bloodstream infection with 1.0 x 10^7 CFU of WT, Δ*pta,* Δ*pta* + *pta,* Δ*lipL,* Δ*lipL* + *lipL*, Δ*pta* Δ*lipL,* Δ*pta* Δ*lipL* + *pta, and* Δ*pta* Δ*lipL* + *lipL* strains. Animal numbers displayed are as follows: WT, N = 36; Δ*pta,* N = 29; Δ*pta* + *pta,* N = 28; Δ*lipL,* N = 20; Δ*lipL* + *lipL,* N = 19; Δ*pta* Δ*lipL,* N = 20; Δ*pta* Δ*lipL* + *pta,* N = 8; and Δ*pta* Δ*lipL* + *lipL,* N = 8. ns, not significant; *, p < 0.05; ***, p < 0.001; ****, p < 0.0001 by Kruskal-Wallis test with Dunn's post hoc analysis. **(B)** Bacterial burden in the skin of mice 72 hours after intradermal infection with 1.0 x 10^7 CFU of WT, Δ*pta,* Δ*pta* + *pta,* Δ*lipL,* Δ*lipL* + *lipL*, Δ*pta* Δ*lipL,* Δ*pta* Δ*lipL* + *pta,* and Δ*pta* Δ*lipL* + *lipL*. Animal numbers displayed are as follows: WT, N = 16; Δ*pta,* N = 16; Δ*pta* + *pta,* N = 16; Δ*lipL,* N = 16; Δ*lipL* + *lipL,* N = 16; Δ*pta* Δ*lipL,* N = 16; Δ*pta* Δ*lipL* + *pta,* N = 16; and Δ*pta* Δ*lipL* + *lipL,* N = 16. ns, not significant; **, p < 0.01; ****, p < 0.0001 by Kruskal-Wallis test with Dunn's post hoc analysis. $\log_{10}$ CFU per organ or abscess is displayed for each infected mouse along with the median as a measure of central tendency. Graphs represent combined data from at least two independent experiments.

https://doi.org/10.1371/journal.ppat.1014183.g005

LipL might also drive a metabolic landscape that is shifted in a Δ*lipL* mutant on account of disruptions to TCA cycle activity and branched fatty acid synthesis. To test this possibility, we grew WT and Δ*lipL* mutant strains to late-exponential phase and performed bulk RNA-sequencing. Our results confirmed several major changes in gene expression patterns between the WT and Δ*lipL* mutant (Fig 6B and 6C). The Δ*lipL* mutant significantly upregulated genes involved in virulence, histidine catabolism and oxidative metabolism and significantly downregulated genes involved in purine nucleotide biosynthesis and branched-chain amino acid biosynthesis (Fig 6D). Specifically, the loss of *lipL* increased expression of several cytoxin genes (*lukS-PV, hla, psmβ1, and psmβ2)*, *gudB* (glutamate dehydrogenase), the *hut* operon (histidine uptake) and the *qox* operon (electron transport chain), while decreasing expression of the *pur* operon (purine biosynthesis), as well as the *leu* and the *ilv* operons (branched-chain amino acid biosynthesis) (Fig 6E). Furthermore, we saw substantially increased levels of the pyruvate oxidase *cidC* (SAUSA300_2477) (Fig 6C, 6E and 6F). CidC generates acetate directly from pyruvate and does not require PDH activity [37]. We also saw increased levels of *alsD* transcripts (Fig 6E). AlsSD diverts carbon flow towards neutral byproducts to prevent CidC-mediated acidification of the cell [36]. To validate our RNAseq results, we assessed *cidC* transcript levels in WT, Δ*pta,* Δ*lipL,* Δ*pta* + *pta*, and Δ*lipL* + *lipL* strains via qRT-PCR. Indeed, we found a ~ 8-fold increase of *cidC* transcripts in both the Δ*pta* and Δ*lipL* mutants (Fig 6F). Overall, RNAseq analysis confirmed that the loss of *lipL* significantly alters the *S. aureus* transcriptome, including changes to pathways involved in virulence, oxidative metabolism, amino acid metabolism, and nucleotide biosynthesis. Furthermore, the RNAseq dataset revealed increased levels of *cidC* transcripts, which were confirmed by qRT-PCR in both the Δ*pta* and Δ*lipL* mutants.

The deletion of both *pta* and *cidC* significantly reduces acetate production by *S. aureus*

Prior studies showed that inactivation of the Pta-AckA pathway led to redirection of carbon into the TCA cycle [28,53]. Furthermore, loss of LipL or Pta leads to transcriptional changes that are suggestive of premature entry into the TCA cycle,

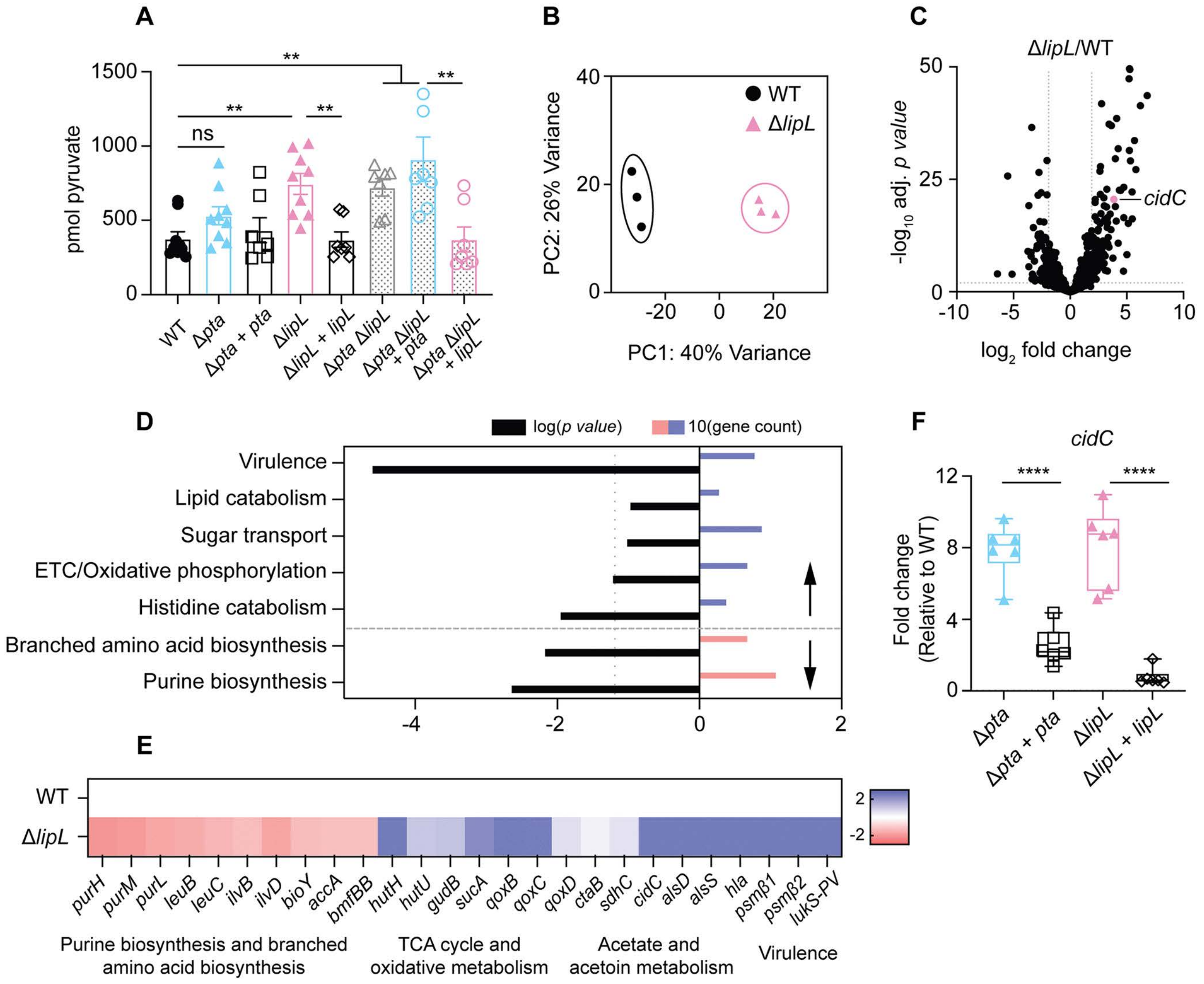

Fig 6. Loss of *lipL* induces significant transcriptional changes in *S. aureus*. **(A)** Quantification of intracellular pyruvate from WT, Δ*pta*, Δ*lipL*, Δ*pta* + *pta*, Δ*lipL* + *lipL*, Δ*pta* Δ*lipL*, Δ*pta* Δ*lipL* + *pta*, and Δ*pta* Δ*lipL* + *lipL* strains after 3 hours of growth in TSB. ns, not significant; **, $p < 0.01$ by Kruskal-Wallis test with Dunn's post hoc analysis. Graphs represent combined data from three independent experiments. **(B)** Principal component analysis (PCA) of RNA sequencing datasets for the WT and Δ*lipL* mutant triplicate samples. **(C)** Volcano plot of transcriptional changes observed in a Δ*lipL* mutant compared to WT *S. aureus* during late-exponential phase growth. Each dot represents one gene. Vertical dotted lines represent $\log_2$ fold change = -2 and 2 cut-offs. Horizontal dotted line represents adj. p value = 0.05. **(D)** Analysis of upregulated and downregulated pathways in a Δ*lipL* mutant compared to WT. Black bars represent log(p-value); vertical dotted line is p-value < 0.05 threshold of significance as determined by NIH DAVID Bioinformatics pipeline. Blue bars represent the number of downregulated genes associated with the respective pathway, and pink bars represent the number of upregulated genes associated with the respective pathway. **(E)** Fold change of specific genes in the Δ*lipL* mutant compared to WT. (F) qRT-PCR analysis of RNA extracted from WT, Δ*pta*, Δ*lipL*, Δ*pta* + *pta*, and Δ*lipL* + *lipL* strains after 3 hours of growth in TSB. ****, p < 0.0001 by one-way ANOVA with Tukey's post hoc test. qRT-PCR experiments were conducted with two independent biological replicates in technical triplicate. Boxplots indicate the median and quartiles. Whiskers indicate the range.

https://doi.org/10.1371/journal.ppat.1014183.g006

increased oxidative metabolism, and increased acetate production by CidC (Fig 6F) [28]. Increased *cidC* levels in both Δ*pta* and Δ*lipL* strains signify demand for acetate production in *S. aureus* regardless of whether the block occurs before or after PDH. To confirm prior studies and test if Pta and CidC are the primary sources of acetate, we generated an in-frame deletion of *cidC* in both WT and a Δ*pta* mutant of *S. aureus*. The Δ*cidC* mutant had no effect on growth in TSB, whereas a

Δ*pta* Δ*cidC* double mutant had a marginal delay in transition to stationary phase growth (Fig 7A), like the Δ*pta* single mutant (Fig 2A). The Δ*pta* Δ*cidC* and Δ*pta* Δ*cidC* + *cidC* strains, but not the Δ*cidC* or the Δ*pta* Δ*cidC* + *pta* strains had evidence of increased lipoylation on E2 subunits consistent with more lipoylation occurring in the absence of Pta, but not CidC (Fig 7B).

When measuring glucose, acetate, lactate, and formate levels, we noted the Δ*cidC* mutant consumed all glucose in the media by 6 hours with no difference compared to the WT (Fig 7C). Peak acetate production was ~ 30 mM for all strains (Fig 7C). Furthermore, there was negligible production of lactate and formate (Fig 7C). The Δ*pta* Δ*cidC* double mutant also

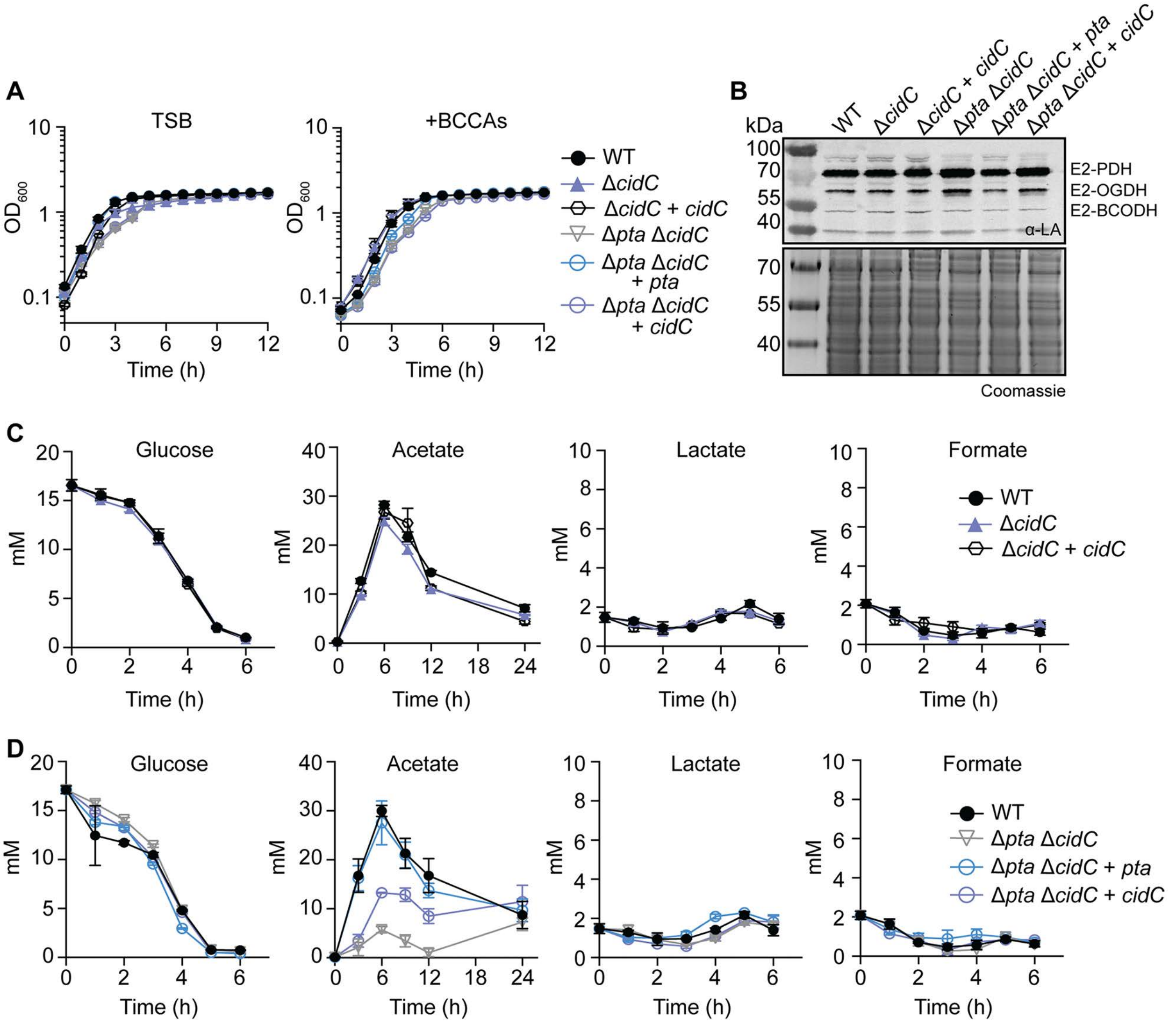

Fig 7. The deletion of both *pta* and *cidC* significantly reduces acetate production by *S. aureus*. (A) Growth of WT, Δ*cidC*, Δ*pta* Δ*cidC*, Δ*cidC* + *cidC*, Δ*pta* Δ*cidC* + *pta*, and Δ*pta* Δ*cidC* + *cidC* strains in TSB and TSB + BCCAs [10 mM isobutyric acid (IB), 9 mM 2-methylbutyric acid (2MB), 9 mM isovaleric acid (IV) + 10 mM sodium acetate (NaAc)] containing 14 mM glucose. **(B)** α-lipoic acid immunoblots of whole cell lysates from the indicated strains after 9-hour subculture growth in TSB medium. The presented blot and Coomassie-stained gel are representative of three independent experiments. **(C)** Quantification of glucose, acetate, lactate, and formate from WT, Δ*cidC, and* Δ*cidC* + *cidC* culture supernatants over time in TSB with 14 mM glucose. **(D)** Quantification of glucose, acetate, lactate, and formate from WT, Δ*pta* Δ*cidC*, Δ*pta* Δ*cidC* + *pta*, and Δ*pta* Δ*cidC* + *cidC* culture supernatants over time in TSB with 14 mM glucose. Each metabolite quantification assay is representative of three independent experiments, with each timepoint measured in technical triplicate. Error bars indicate standard deviation from the mean.

https://doi.org/10.1371/journal.ppat.1014183.g007

consumed all glucose by 6 hours and had no delay in the glucose consumption compared to the WT (Fig 7D). Consistent with previous reports, there was negligible production of acetate from the Δ*pta* Δ*cidC* double mutant (~5 mM, a ~ 83% reduction compared to WT). (Fig 7D) [28]. Peak acetate production from the Δ*pta* Δ*cidC* + *cidC* was ~ 13 mM (Fig 7D), whereas acetate production from Δ*pta* Δ*cidC* + *pta* was the same as WT (Fig 7D). There was no lactate or formate produced, consistent with our earlier observations that compensatory lactate fermentation only occurs when PDH is inactivated (Fig 7D). These data corroborate prior work and indicate that *cidC* is dispensable for *S. aureus* growth in broth yet is activated to compensate for the loss of Pta to promote acetate production (compare Figs 2C and 7D) [28,36].

Acetate production is required for *S. aureus* infection

In prior studies, CidC was implicated in the pathogenesis of biofilm-associated infection in the heart, suggesting CidC has a role in virulence [36]. Furthermore, the increase of *cidC* transcripts in the Δ*pta* and Δ*lipL* mutants (Fig 6F) led us to hypothesize that acetate generation via CidC could be an important compensatory strategy to promote *S. aureus* survival *in vivo*, especially if acetate generation in and of itself were important for infection. To begin to test this possibility, we determined if *cidC* was required for *S. aureus* bloodstream and skin infection using planktonic cultures. We infected mice intravenously or intradermally with WT, Δ*cidC*, and Δ*cidC* + *cidC* strains. At 96 hours post intravenous infection, we isolated and homogenized kidneys from Δ*cidC* mutant-infected animals and found no difference in CFUs compared to WT (Fig 8A). At 72 hours post intradermal infection, there was also no difference in CFUs between strains (Fig 8A). We then infected mice intravenously or intradermally with WT, Δ*pta* Δ*cidC*, Δ*pta* Δ*cidC* + *pta,* and Δ*pta* Δ*cidC* + *cidC* strains. After systemic infection, the kidneys from Δ*pta* Δ*cidC* double mutant-infected animals had ~10000-fold fewer CFUs compared to WT (Fig 8B). This result was in stark contrast to a Δ*pta* mutant, which had a ~ 100-fold reduction in CFUs (Fig 5A). Complementation of the double mutant with *pta* restored kidney CFUs to nearly WT levels (Fig 8B), whereas complementation of the double mutant with *cidC* partially restored virulence to levels previously observed for a Δ*pta* mutant (~100-fold fewer CFUs compared to WT) (Fig 8B). At 72 hours post intradermal infection, we found ~100-fold fewer CFUs were recovered from the abscesses of Δ*pta* Δ*cidC* double mutant compared to WT (Fig 8B). Like the kidneys, complementation with *pta* restored CFU levels to WT levels (Fig 8B), whereas complementation with *cidC* in the skin was not evident (Fig 8B).

Among the pathways significantly upregulated in RNAseq analysis of a Δ*lipL* mutant (Fig 6E) were genes involved in virulence. Toxin genes, including *hla*, *psmβ1*, *psmβ2*, and *lukS-PV*, were amongst the most significantly upregulated (Fig 6F). These toxin genes are all regulated by the Accessory Gene Regulatory (Agr) two-component quorum sensing system [54,55]. Despite the increase in Agr-dependent virulence gene expression, a Δ*lipL* mutant remained attenuated *in vivo* (Fig 5) arguing that virulence factor expression is not contributing to *in vivo* phenotypes in this strain. Nevertheless, this outcome prompted us test if *in vivo* phenotypes linked to acetate production were potentially due to changes in Agr activity. To test this possibility, we infected mice intravenously or intradermally with WT, Δ*agr::tet*, Δ*pta* Δ*cidC*, and Δ*pta* Δ*cidC* Δ*agr::tet* strains. At 96 hours post-intravenous infection, kidneys from Δ*agr::tet* mutant-infected animals had similar CFUs to WT (Fig 8C), consistent with previous reports [56,57]. Conversely, kidneys from Δ*pta* Δ*cidC* and Δ*pta* Δ*cidC* Δ*agr::tet* mutant-infected animals had identical reduction in CFUs compared to WT (~10000-fold) (Fig 8C). At 72 hours post intradermal infection, abscesses from mice infected with Δ*agr::tet* mutant had ~10-fold fewer CFUs compared to WT, which is consistent with previous literature [58] (Fig 8C). Abscesses from mice infected with Δ*pta* Δ*cidC* double mutant had ~100-fold fewer CFUs compared to WT (Fig 8C). Mice infected with Δ*pta* Δ*cidC* Δ*agr::tet* triple mutant had ~1000-fold fewer CFUs, demonstrating an additive effect (Fig 8C). Taken together, these data suggest that acetate production mediated by Pta and CidC is required for infection and is Agr independent.

Discussion

In this study, we investigated the genetic and functional link between lipoic acid transfer and acetate production by *S. aureus*. We found that the genes encoding phosphotransacetylase, Pta, and the lipoic acid amidotransferase, LipL,

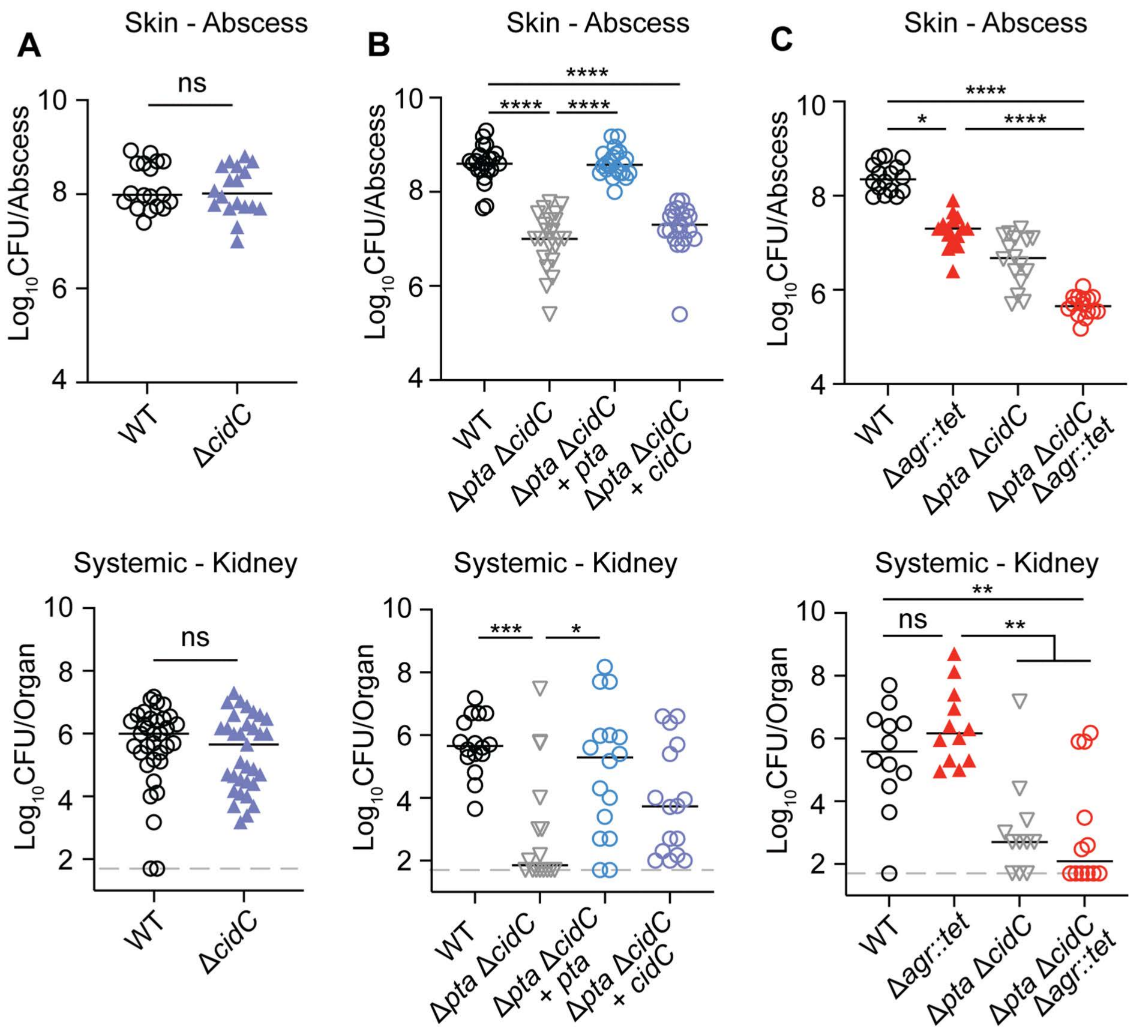

Fig 8. Acetate production is required for *S. aureus* infection. (A) Upper panel - Bacterial burden in the skin of mice 72 hours after intradermal infection with 1.0 x 10^7 CFU of WT and Δ*cidC* strains. Animal numbers displayed are as follows: WT, N = 18; Δ*cidC,* N = 18. Lower panel - Bacterial burden in the kidneys of mice 96 hours after bloodstream infection with 1.0 x 10^7 CFU of WT, Δ*cidC* strains. Animal numbers displayed are as follows: WT, N = 34; Δ*cidC,* N = 33. NS, not significant. Statistics were calculated by Mann-Whitney test. **(B)** Upper panel - Bacterial burden in the skin of mice 72 hours after intradermal infection with 1.0 x 10^7 CFU of WT, Δ*pta* Δ*cidC,* Δ*pta* Δ*cidC* + *pta,* and Δ*pta* Δ*cidC* + *cidC* strains. Animal numbers displayed are as follows: WT, N = 29; Δ*pta* Δ*cidC,* N = 29; Δ*pta* Δ*cidC* + *pta,* N = 28; Δ*pta* Δ*cidC* + *cidC,* N = 28. Lower panel - Bacterial burden in the kidneys of mice 96 hours after bloodstream infection with 1.0 x 10^7 CFU of WT, Δ*pta* Δ*cidC,* Δ*pta* Δ*cidC* + *pta,* and Δ*pta* Δ*cidC* + *cidC* strains. Animal numbers displayed are as follows: WT, N = 16; Δ*pta* Δ*cidC,* N = 16; Δ*pta* Δ*cidC* + *pta,* N = 16; Δ*pta* Δ*cidC* + *cidC,* N = 16. ***, $p < 0.001$; ****, $p < 0.0001$ by Kruskal-Wallis test with Dunn's post hoc analysis. **(C)** Upper panel - Bacterial burden in the skin of mice 72 hours after intradermal infection with 1.0 x 10^7 CFU of WT, Δ*agr::tet*, Δ*pta* Δ*cidC,* and Δ*pta* Δ*cidC* Δ*agr::tet* strains. Animal numbers displayed are as follows: WT, N = 16; Δ*agr::tet,* N = 16; Δ*pta* Δ*cidC,* N = 16; Δ*pta* Δ*cidC* Δ*agr::tet,* N = 16. Lower panel - Bacterial burden in the kidneys of mice 96 hours after bloodstream infection with 1.0 x 10^7 CFU of WT, Δ*agr::tet*, Δ*pta* Δ*cidC,* and Δ*pta* Δ*cidC* Δ*agr::tet* strains. Animal numbers displayed are as follows: WT, N = 12; Δ*agr::tet,* N = 12; Δ*pta* Δ*cidC,* N = 12; Δ*pta* Δ*cidC* Δ*agr::tet,* N = 12. *, $p < 0.05$; **, $p < 0.01$; ****, $p < 0.0001$ by Kruskal-Wallis test with Dunn's post hoc analysis. log_{10}CFU per organ or abscess is displayed for each infected mouse along with the median as a measure of central tendency. All graphs represent combined data from at least two independent experiments.

https://doi.org/10.1371/journal.ppat.1014183.g008

are co-transcribed and that this regulatory arrangement is conserved amongst *Staphylococci*. At the protein level, Pta and LipL directly interact and their activities promote acetate fermentation (Pta-AckA) over oxidative metabolism. Deletion of either Pta or LipL significantly hindered acetate production, leading to an intracellular buildup of pyruvate and

compensatory shifts in metabolism. This includes a substantial increase in *cidC* transcript, encoding the pyruvate oxidase, CidC, which generates acetate from direct decarboxylation of pyruvate. The coordinated function of Pta and LipL is required for infection of the skin, whereas the roles of Pta and LipL in systemic infection are separable (Fig 9). Moreover, we found that acetate production by Pta and CidC was required for *S. aureus* infection in both systemic and skin infections. Altogether, our data highlight a previously unidentified mechanism to regulate carbon flow by integrating the dynamic delivery of lipoic acid with metabolite shuttling. This work provides a compelling example of how metabolic adaptations that coordinate nutrient flux are beneficial for *S. aureus* survival in the host by allowing the bacterium to optimize its nutritional resources.

In prior work, we found that the *lipL* open reading frame is positioned two nucleotides downstream of the *pta* gene in *S. aureus,* suggesting the two genes are co-regulated at the transcriptional and likely translational levels (Fig 1E) [26]. RNAseq and qRT-PCR data validated the presence of a single *pta-lipL* transcript (Fig 1E and 1F). This finding raised the question of the frequency with which *lipL* and *pta* genes coincide. *In silico* synteny analyses herein revealed that, within the Gram-positive pathogens assessed, some had *lipL* in close genetic proximity to *pta* (Fig 1B-1D). This phenomenon was not observed in representative Gram-negative pathogens, where, in most instances, *ackA* and *pta* are co-transcribed (Fig 1B). The evolutionary differences between the lipoic acid biosynthesis and salvage pathways of Gram-positive and Gram-negative bacteria offer some insight into these *in silico* results. The lipoic acid biosynthesis pathway in *E. coli* and

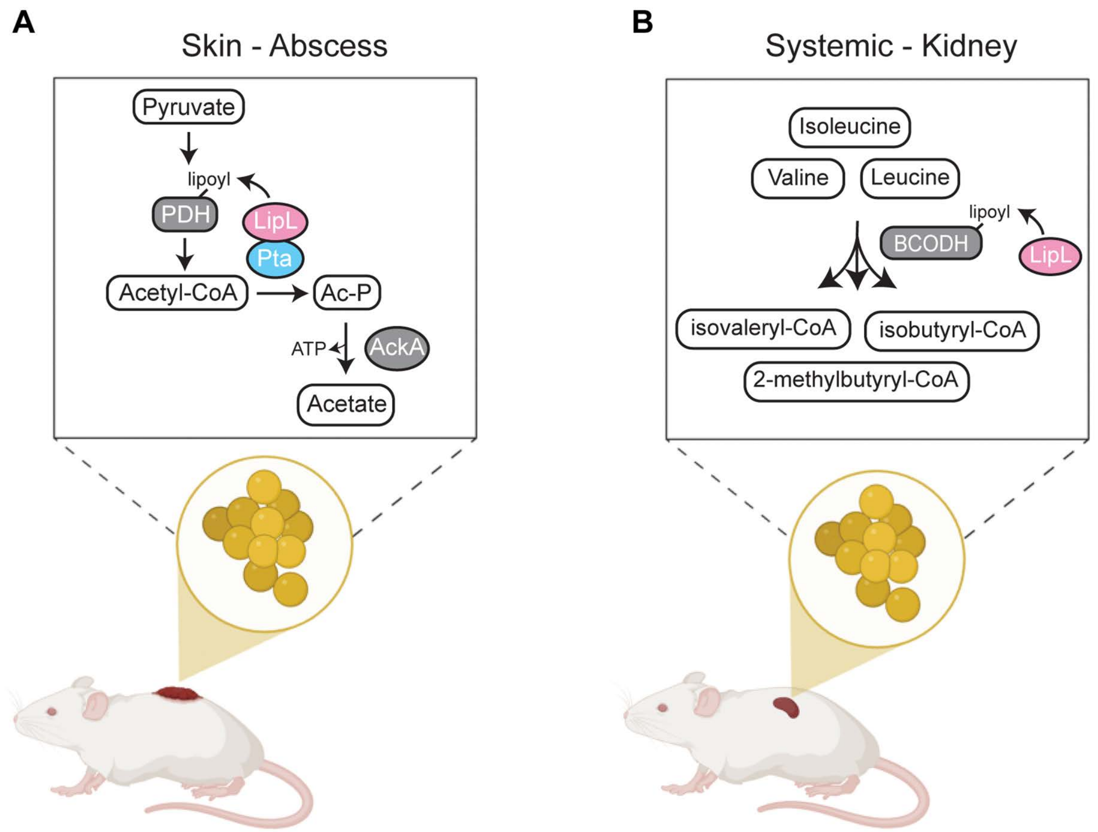

Fig 9. Proposed model of tissue-specific LipL-mediated requirement for virulence. (A) During skin and soft tissue infection, Pta and LipL activities coordinate metabolic flux through the Pta-AckA pathway. **(B)** During systemic infection, there is a dominant role for LipL-dependent lipoylation of BCODH that is vital for BCFA synthesis and *S. aureus* survival, which is distinct from roles for Pta in virulence. Figure Created in BioRender. Woods, R. (2026) https://BioRender.com/vbnk4gt.

https://doi.org/10.1371/journal.ppat.1014183.g009

other Gram-negatives consists of two enzymes, LipA and LipB [25,59,60]. The octanoyltranferase, LipB, attaches octanoic acid directly to E2 and H protein subunits, followed by sulfur insertion by LipA. Thus, lipoic acid is synthesized directly on each metabolic enzyme complex [25,59,60]. Notably, Gram-negatives do not encode amidotransferases such as LipL and thus do not perform lipoyl relay between E2 and H proteins [25]. On the other hand, Gram-positives harbor a more complex lipoic acid biosynthesis pathway that includes LipM, a transferase that delivers octanoic acid to the H protein of GcvH, the sole site of sulfur insertion by LipA [25,61,62]. The transfer of lipoic acid from GcvH to other enzyme complexes is mediated by LipL. Without LipL, complete transfer to E2 subunits does not occur [1,26,62]. Furthermore, the presence of LipL confers a dynamic flexibility that potentially allows for dynamic redistribution of lipoic acid based on the metabolic state of the cell [1,25,26,62]. The absence of LipL in Gram-negatives and direct synthesis on each enzyme complex precludes the regulatory advantages offered by a lipoyl relay pathway. These fundamental differences may explain why lipoic acid synthesis and carbon overflow pathways are only linked in Gram-positives. Indeed, the transcriptional coupling of *pta* and *lipL* in Staphylococci hinted at a direct coupling of lipoic acid transfer and acetate fermentation that was validated in this work.

Assays of acetate levels in Δ*pta* and Δ*lipL* mutants revealed different degrees of impairment in acetate production, where a Δ*pta* mutant produced more acetate over time compared to a Δ*lipL* mutant (Fig 2C and 2D). This is likely accounted for by the fact that a Δ*lipL* mutant also produces substantial lactate, suggesting that blockade upstream of PDH necessitates redirection of pyruvate through both lactate fermentation and CidC (Fig 2D). In support of this point, the Δ*lipL* mutant also had considerably higher pyruvate levels (Fig 6A). In addition to these observations that center on pyruvate accumulation, our RNA sequencing data also showed that loss of *lipL* led to a global change to the transcriptome including upregulation of genes involved in histidine catabolism (*hut* operon) and oxidative metabolism (*qox* operon) and downregulation of genes involved in branched-chain amino acid biosynthesis (*leu* and *ilv* operons) and purine biosynthesis (*pur* operon) (Fig 6D and 6E). The downregulation of genes involved in branched-chain amino acid biosynthesis was accompanied by the downregulation of *bmfBB* (encoding E2-BCODH), *accA* (a subunit of acetyl-CoA carboxylase), and *bioY* (biotin transporter) (6E). These results suggest that a Δ*lipL* mutation leads to reduced branched-chain fatty acid biosynthesis since *bmfBB* encodes the E2 subunit of the enzyme complex that generates the coA derivatives required for branched chain fatty acid synthesis, while acetyl-CoA carboxylase is required for fatty acid synthesis itself with the biotin transporter aiding in acquisition of biotin, an essential cofactor for this process [63–65]. Conversely, upregulation of the *hut* operon suggests there is increased TCA cycle activity in a Δ*lipL* mutant because it is directly involved in the catabolism of histidine to glutamate, which is directly fed into the TCA cycle via *gudB* [45]. Indeed, *gudB* transcript levels were also elevated in a Δ*lipL* mutant relative to WT (Fig 6E). The shift toward a transcriptional profile that favors TCA cycle activity in a Δ*lipL* mutant is further supported by the fact that, of the lipoylated E2 subunits, only E2-OGDH maintains its lipoylation status in the absence of LipL (Fig 2B) [1,5,26]. The residual lipoylation of E2-OGDH is mediated by the *S. aureus* encoded lipoic acid ligases, LplA1 and LplA2 [1,66]. Furthermore, our RNAseq data showed increased *sucA* (encodes E1-OGDH) transcripts (Fig 6E), supporting the idea that TCA cycle activity is increased in a Δ*lipL* mutant (Fig 2B). A Δ*lipL* mutant also had increased expression of genes with roles in oxidative metabolism (Fig 6D) such as the *qox* operon, *sdhC* and *ctaB* (Fig 6E). The *qox* operon is involved in aerobic respiration, *sdhC* is a gene involved in both the TCA cycle and the electron transport chain, and *ctaB* produces the heme O cofactor required for QoxABCD terminal oxidase function [67–71]. All together these data support the idea that LipL has a key role in precisely modulating *S. aureus* metabolic flux, favoring acetate fermentation while limiting the TCA cycle and oxidative metabolism.

Our RNAseq analysis also uncovered several other genes that were upregulated in a Δ*lipL* mutant. We observed a significant increase in *cidC* transcripts, which was corroborated by qRT-PCR (Fig 6E and 6F). These results validate work from others, which suggests that induction of *cidC* represents a compensatory strategy to overcome intracellular pyruvate accumulation [28,36]. CidC is encoded in an operon alongside genes encoding the holin, CidA, and a potential support protein, CidB [34,72]. Transcription of the *cid* operon is under the control of multiple promoters and transcriptional

regulators [33,72,73]. During exponential phase growth, sigma factor B (σB) induces the expression of *cidBC* [72]. During stationary phase, the LysR-type transcriptional regulator CidR induces expression of *cidABC* [39,33]. CidR induction of *cidABC* is known to promote maintenance of biofilm structure, where the holin activity of CidA aids in promoting bacterial cell death [39]. To determine if σB or CidR-dependent transcriptional upregulation was occurring in a Δ*lipL* mutant, we assessed our RNAseq dataset for *cidA* or *cidB* transcripts. Intriguingly, we found that the baseMean values for *cidC* were 3231.85, compared to 611.48 for *cidB* and 161.65 for *cidA,* suggesting the *cidC* transcript was upregulated independent of *cidA* and *cidB* in this scenario. Furthermore, a read analysis of *cidA*, *cidB*, and *cidC* showed a markedly higher read density across *cidC* compared to *cidA* and *cidB* (S2B Fig). The elevated *cidC* signal, coupled with its higher baseMean value, suggests the possibility of an alternative transcriptional start site for *cidC* that is independent of *cidAB*. Future work will address this possibility.

Work from Sadykov *et al.* highlights the importance of Pta and AckA for *S. aureus* fitness and metabolism. They found that the metabolome of both a Δ*pta* and a Δ*ackA* mutant is marked by a redirection of carbon flux into glycolysis, TCA cycle, pentose phosphate pathway, and amino acid biosynthetic pathways [28]. Our lipoylation profile data support this redirection of metabolic flux toward glycolysis and TCA cycle, wherein we noted increased lipoylation of E2-PDH and E2-OGDH in the Δ*pta* mutant (Fig 4D and 4E). Furthermore, we found that a Δ*pta* mutant does not have a commensurate increase in *pdhC*, or *sucB* transcripts (S2A Fig), suggesting the increase in lipoylation on E2-PDH and E2-OGDH is due to a redirection of lipoic acid transfer by LipL. Sadykov *et al.* also found that the loss of *pta* causes a fitness defect in which there is a delay in exponential phase growth, which we reproduced herein (Fig 2A) [28]. Interestingly, an *ackA* mutant in *S. aureus* has a faster growth rate during exponential phase compared to a *pta* mutant [28]. These results suggest the coordinated activity of Pta and LipL in *S. aureu*s, which occurs upstream of AckA activity, is more critical to overall fitness. Intriguingly, the reduced exponential phase growth rate of a Δ*pta* mutant compared to a Δ*ackA* mutant appears to be specific to *S. aureus*. Studies in other pathogens suggest a Δ*ackA* mutant has a more severe effect on growth rate compared to a Δ*pta* mutant, citing accumulation of acetyl-phosphate as the potential culprit [28,29,74,75]. This was the case for strains where *ackA*-*pta* were in an operon (*E. coli*), *pta* and *lipL* were in close genetic proximity but not linked (*B. subtilis*), and strains where *pta* and *lipL* were divergently transcribed (*B. anthracis*) [29,53,76]; thus, it is difficult to draw conclusions on the basis of these observations from gene positioning alone. Nevertheless, these prior studies coupled with our *in-silico* analysis (Fig 1B–1D) suggest that *Staphylococci* have adapted in ways that allow coordination of Pta and LipL activity to maintain fine-tuned control over metabolic flux through pathways that are critical for fitness.

Functional and computational studies suggest that metabolic flux is enhanced through improved channeling of substrates from one enzyme to another via protein complex formation and protein-protein interactions [13–15,77]. We used AlphaFold to predict the likelihood of a direct interaction between Pta and LipL and confirmed this model with *in vitro* and *in vivo* evidence (Fig 4). These studies suggest, at minimum, Pta and LipL may interact to drive metabolism in the direction of Pta-AckA. In these experiments, Pta and LipL had components of adenylate cyclase or 6x-his epitope tags appended to the C-terminus of each protein. Efforts to append CyaA fragments or epitope tags to the N-terminus prevented detection of an interaction between Pta and LipL (S2C Fig). Prior structural analysis of Pta in Gram-negative and positive organisms revealed that the protein contains two functional domains separated by an interdomain cleft, which is vital for the binding of acetyl-phosphate [78–80]. Crystal structures of Pta in Gram-positive organisms such as *B. subtilis* and *S. pyogenes* revealed that the N-terminus of Pta is a component of this interdomain cleft and may provide a potential reason why the N-terminus of Pta is not a productive site for epitope tags, as it is required for binding of substrates and presence in the interdomain cleft may preclude attachment of even small tags [78,80,81]. Future studies in our lab aim to identify specific residues critical for the Pta–LipL interaction and to test their impact on metabolite flux.

Additionally, previous biochemical assessments of Pta in Gram-negatives such as *E. coli* and *Salmonella enterica* Typhimurium found that the N-terminus of Pta serves a regulatory role through the allosteric binding of cofactors and substrates, such as NADH and pyruvate [82,83], whereas biochemical studies in Gram-positives reveal Pta activity is

not altered by allosteric effectors [81]. Our inability to generate epitope tags of Pta or LipL that maintain protein-protein interaction characteristics suggests that the N-terminus of *S. aureus* Pta is, at minimum, important for interactions (S2C Fig). The increase in LipL-dependent lipoylation of E2-PDH and E2-OGDH in the absence of Pta could imply that LipL increases delivery of lipoic acid to E2 subunits due to increased metabolic demand or that Pta negatively impacts LipL function (Figs 2B, 4E–4D, and 7B). It is also possible that LipL improves Pta function. Future biochemical experiments will directly examine these possibilities [84–86]. Furthermore, our bacterial two-hybrid assays also detected evidence for an interaction between Pta and E2-PDH (S2C Fig); however, Alphafold modeling of the interaction had low predictive values (PTM of 0.47 and iPTM of 0.2) (S1D Fig). Nevertheless, the bacterial two-hybrid results may support the possibility of a larger complex between Pta, LipL, and PDH that improves substrate delivery. Additional evidence in support of this idea includes a protein interaction network analysis in *S. aureus* that identified an interaction between Pta and E2-PDH [87]. Further assessment of a potential Pta-LipL-PDH complex is warranted. We are currently pursuing these areas of investigation.

In a systemic infection model, the roles of Pta and LipL in virulence are separable (Fig 5A). While Pta contributes to infection in the kidney, the attenuation of Δ*lipL* and Δ*pta* Δ*lipL* mutants is far more dramatic, with the Δ*lipL* mutant and Δ*pta* Δ*lipL* mutants phenocopying each other (Fig 5A). LipL is known to transfer lipoic acid to enzyme complexes that extend beyond overflow metabolism pathways and include enzymes that facilitate branched-chain fatty acid synthesis (BCODH) and TCA cycle activity (OGDH). In prior work, we noted a strong dependency on branched-chain fatty acid synthesis during systemic infection that is bypassed in the skin via uptake of host unsaturated fatty acids [5]. Thus, the contribution of LipL to infection in the kidney is largely driven by deficiencies in branched-chain fatty acid synthesis, whereas Pta contributes to a lesser degree (Fig 5A). This conclusion is further supported by the finding that constitutive expression of *lipL*, but not *pta*, in a Δ*pta* Δ*lipL* mutant background nearly restores full virulence in the kidneys (Fig 5A). In contrast, Δ*pta,* Δ*lipL,* and Δ*pta* Δ*lipL* strains phenocopy one another (Fig 5B) for infection defects in the skin, implying a direct role for flux through PDH and toward Pta-AckA in skin infection. In agreement with this idea, complementation with either *pta* or *lipL* alone fails to restore full virulence in the skin (Fig 5B). These results further cement the importance of regulating acetate production during *S. aureus* skin infection (Fig 9).

Several studies have considered the roles of *S. aureus* acetate production genes during infection. One study found that infection with a Δ*ackA* mutant had reduced lesion area during skin infection of hyperglycemic mice [40]. Another study assessed a Δ*cidC* mutant in a rabbit model of infective endocarditis and found no difference between the Δ*cidC* mutant and WT at the primary site of the vegetation [36]. Instead, the authors found that a Δ*cidC* mutant had significantly fewer CFUs in secondary sites of infection, leading them to suggest CidC may play a role in dispersal from biofilms in the host [36]. Here, we find that a requirement of acetate production is not contingent on a high glycemic status (mice are on standard chow). Furthermore, our Δ*cidC* mutant infection data suggest that during planktonic growth, CidC alone is not required for infection. However, infection with a Δ*pta* Δ*cidC* strain leads to dramatic attenuation compared to either single deletion, implying compensatory demand for acetate production *in vivo* (Fig 8B). Altogether, this infection data highlights the critical importance of acetate-producing genes in several models of infection.

Recent work from Thurlow *et al.* determined that glycolytic flux enhances activity of the accessory gene regulatory (Agr) system [40]. Agr is a two-component system (TCS) that responds to changes in bacterial cell density and is a master regulator of virulence factors, controlling the expression of leukotoxins, hemolysins, lipases, and proteases [88–90]. Agr-dependent toxin production by *S. aureus* requires ATP production from processes such as overflow metabolism through Pta-AckA [40]. The requirement of glycolysis to enhance Agr-dependent gene expression is accounted for by a reduced affinity of AgrC, the sensor histidine kinase of the Agr system, for ATP [40,91,92]. Thus, ATP must be high for maximal production of Agr-dependent virulence factors in *S. aureus*. We found that a Δ*lipL* mutant increases oxidative metabolism to maintain energy production when flux through the PDH node is blocked (Fig 6E–6F). RNAseq analysis further revealed increased expression of Agr-regulated virulence genes in the Δ*lipL* mutant (Fig 6E–6F). Although not yet directly tested,

these findings raise the possibility that ATP generated through oxidative metabolism might also promote Agr-dependent virulence factor production. Regardless, these studies further support the idea that the metabolic status of *S. aureus*, as defined by the nutritional environment, can have direct impacts on pathogenic potential.

The innate immune system serves as the body's first line of defense against bacterial infections, with macrophages and neutrophils acting as key early responders that initiate the host immune response. Our data demonstrates that the loss of acetate production is detrimental for *S. aureus* survival in the host (Fig 8), suggesting that acetate might play a role in modulating the host response to infection. Given this, we suspect that *S. aureus*-derived acetate might alter the function of innate immune cells. Indeed, acetate is a ligand for the G-protein-coupled receptors FFAR2 and FFAR3, which are present on the surface of macrophages and neutrophils [93–96]. FFAR2 primes innate immune cells, facilitating productive immune responses during bloodstream, gastrointestinal, and pulmonary infections caused by both Gram-negative and Gram-positive pathogens [97–100]. More recent work from Xu *et al* showed that acetate and formate produced by *S. pyogenes* inhibited innate immune cell accumulation and cytokine production, which delayed host wound healing and bacterial clearance during soft tissue infections [101]. Thus, a paradox emerges in which acetate can either enhance or suppress the innate immune response. Given the importance of Pta and CidC for *S. aureus* infection (Fig 8), our data may imply a role for acetate in dampening the innate immune response. Ongoing work aims to study the potential impact of acetate on innate immune cells during *S. aureus* infections.

Collectively, this work supports the idea that *S. aureus* coordinates metabolic flux through transcriptional and post-transcriptional mechanisms to promote adaptation and survival during infection. These findings have implications for our understanding of coordinated enzyme complex assembly that drives the delivery of key metabolites to their targets and argue for a potential direct role for acetate production in bacterial virulence. Further, it highlights the importance of tissue-specific dependencies on major metabolic processes for virulence, which supports a critical examination of pathogenesis using several infection models.

Methods

Ethics statement

All animal experiments were conducted in ABSL2 facilities under Institutional Animal Care and Use Committee (IACUC)-approved protocols from the University of Illinois Chicago (Protocol #25–042) and Loyola University Chicago Health Sciences Division (Protocol #2020025), in accordance with United States Department of Agriculture (USDA), Public Health Service (PHS), and Office of Laboratory Animal Welfare (OLAW) guidelines. The University of Illinois Chicago holds a current PHS Animal Assurance (#A3460-01, through 04/30/2027), is Association for Assessment and Accreditation of Laboratory Animal Care (AAALAC) accredited (#000186, 04/01/2024), and USDA licensed (#33-R-0018). Loyola University Chicago holds a current PHS Animal Assurance (#A3460-01, through 02/28/2026), is AAALAC-accredited (#000180, 11/18/2022), and USDA-licensed (#33-R-0024). All procedures complied with institutional ethical standards.

Phylogenetic tree construction and synteny analysis

The *pta* coding sequence was used in independent searches of the Seed analysis viewer (https://pubseed.theseed.org/FIG/seedviewer.cgi?page=Minimal) [102,103]. The *pta* gene in *Staphylococcus aureus* subsp. *aureus* USA300 used for search was taxon ID 367830; feature ID fig|367830.3.peg.650) and is annotated as phosphate acetyltransferase (EC 2.3.1.8). All genomes were compared by a standard search of the Seed database. The genomes of *M. tuberculosis* and selected Gram-positive and Gram-negative bacteria representing the most frequent genetic organizations of *pta*, *lipL,* and *ackA* were then chosen for subsequent analysis. Taxonomic assignments for these genomes were confirmed in NCBI (TaxIDs: 242231, 604162, 99287, 575584, 1051003, 272620, 83333, 196600, 187410, 451515, 224308, 1027396, 160490, 1773, 226185, 416870, 373153, 191218, 1385523, 1242971, 1155131, 904338, 1189311, 435837, 1134914,

342451). Phylogenetic trees were constructed using the selected genomes in $PhyloT_{v2}$ (https://phylot.biobyte.de) and visualized using the interactive Tree of Life tool, $iTOL_{v7}$ (https://itol.embl.de) [104].

Bacterial strains and growth conditions

The strains used in this study are described in S1 Table. *Escherichia coli* strains were grown in Lysogeny Broth (LB) (BD Biosciences), and *S. aureus* strains were grown in Tryptic Soy Broth (TSB) (BD Biosciences). For experiments with no or low glucose (3.5 mM), *S. aureus* strains were growin in TSB without dextrose (BD Biosciences) with or without D-glucose supplementation (Amresco). Unless stipulated, strains were grown at 37°C, shaking at 200 RPM. For strains harboring a Δ*lipL* mutation, branched-chain carboxylic acids (BCCAs) (10 mM isobutyric acid [Sigma-Aldrich], 9 mM 2-methylbutyric acid [Alfa Aesar], 9 mM isovaleric acid [Sigma-Aldrich]) and 10 mM sodium acetate (NaAc; Sigma-Aldrich) were added to the media to promote growth [23,61]. When needed, media were supplemented with the following agents of selection: ampicillin (100 µg/ml), chloramphenicol (10 µg/ml), kanamycin (50 µg/ml), cadmium chloride (0.1 mM), anhydrous tetracycline (1 µg/ml) (AnTet), and sodium citrate (10 mM).

Generation of in-frame deletion mutants

Oligonucleotide pairs (S2 Table) were designed to amplify fragments of ~500–1000 nucleotides upstream and downstream of the targeted genes for mutagenesis using *S. aureus* LAC genomic DNA as a template. Purified amplicons were then used as templates in a splicing by overlap extension (SOE) PCR to yield the final 1,000–2,000 bp amplicon containing a fusion of the upstream and downstream gene fragments. Each amplicon was digested with KpnI and SacI restriction endonucleases and ligated into the multiple cloning site (MCS) of pIMAY using T4 DNA ligase [105]. Ligation reactions were transformed into *E. coli* IM08B [46], and transformants containing the ligated amplicon were verified by PCR. Plasmids were purified from IM08B and subsequently transformed into *S. aureus* competent cells by electroporation (1800 V, 10 µF, 600 Ω, 2 mm cuvette). Mutagenesis was performed as previously described [47]. Briefly, *S. aureus* cells containing pIMAY plasmids were grown at 30°C to allow for plasmid replication. Plasmid recombination into the chromosome was induced at 37°C with selection on chloramphenicol. To facilitate plasmid excision, cells were returned to the permissive temperature for plasmid replication (30°C) and passaged without antibiotic to promote plasmid loss. Bacteria were then plated on TSA plates supplemented with AnTet and incubated at 30°C to counter-select for cells that still contain the pIMAY plasmid. Chloramphenicol sensitive and AnTet-resistant colonies were screened for the desired mutation by PCR.

Generation of complement strains and phage transduction

Complementation strains were generated using the pJC1111 plasmid [48]. Genes for complementation were amplified from *S. aureus* genomic DNA using the primer pairs listed in S2 Table, while the constitutive P_{HELP} promoter was amplified from the pIMAY plasmid as template [47]. Purified amplicons were fused by SOE PCR and subsequently cloned into the MCS of pJC1111 using Sac1 and Sal1 restriction endonucleases for digestion and T4 DNA ligase for ligation. The recombinant plasmids were propagated in *E. coli* DH5α and transformed into SaPI-1 integrase-expressing *S. aureus* (RN9011) to allow single-copy integration in the chromosome at the SaPI-1 site [48]. Bacteriophage Φ11 was used to package the integrated complementation plasmids from RN9011, and the phage was used for transduction of genetic material into the recipient strain of interest, as previously described [106]. Briefly, a single colony of the RN9011 donor strain was grown overnight at 37°C with shaking. The overnight culture was diluted to an OD_{600} of 0.2 in TSB. 5 mL of diluted culture was added to 5 mL of TMG buffer (10 mM Tris pH 7.5, 5 mM $MgCl_2$, 0.01% gelatin (v/v), 5 mM $CaCl_2$) in a 15 mL conical tube containing 100 µL of Φ11 phage and incubated at room temperature overnight until lysis was complete. The following day, 100 µL of the filter sterilized phage lysate was added to an overnight culture of the recipient strain and incubated at 37°C with shaking for 20 minutes. The infected recipient was washed three times with 1 mL of 40 mM sodium citrate and

then plated on TSA supplemented with 0.1 mM cadmium chloride and 40 mM sodium citrate to select for transductants. All transductants were confirmed by PCR.

Generation of Δ*agr* Δ*pta* Δ*cidC* strain

A marked Δ*agr*::*tet* mutation was transduced into a Δ*pta* Δ*cidC* mutant strain, as described above, to generate the Δ*agr*::*tet* Δ*pta* Δ*cidC* mutant strain. The Δ*agr*::*tet* mutation spans the *agr* operon [58].

Generation of 6x-Histidine tagged Pta and LipL expression plasmids

The *pta* and *lipL* genes were amplified from *S. aureus* LAC genomic DNA using the primer pairs in S2 Table. The resulting amplicons were cloned into the pET21a (Novagen) expression vector using Nde1 and Sal1 restriction endonucleases for digestion and T4 DNA ligase for ligation. The resulting plasmids were transformed into chemically competent *E. coli* DH5α. Plasmids were purified from DH5α and transformed into chemically competent *E. coli* lysY/I^q (NEB). All recombinant expression plasmids were verified by whole plasmid sequencing (Plasmidsaurus).

Purification of 6x-Histidine-tagged proteins

To purify Pta-6xHis and LipL-6xHis, a single colony of *E. coli* lysY/I^q containing pET21a-*pta-6xHis* or pET21a-*lipL-6xHis* was used to start an overnight culture in 20 mL of LB at 37°C with shaking at 200 RPM. The bacteria were diluted (1:100) in 1 L of fresh LB and incubated at 37°C with shaking at 200 RPM until reaching an OD_{600} of ~0.3. Protein expression was induced by supplementing the culture with IPTG (0.1 mM) and incubating at 37°C for 3 hours with shaking at 200 RPM. Following induction, bacteria were collected by centrifugation at 10000 RPM for 20 min, and pellets were stored at -80°C until use. Frozen bacterial pellets were thawed and resuspended in 60 mL lysis buffer (25 mM imidazole, 50 mM Tris-HCl, 300 mM NaCl, 1 mM phenylmethylsulfonyl fluoride (PMSF), pH 8). The bacterial suspensions were sonicated on an ice bath for 10 minutes with intervals of 10 s ON and 50 s OFF at an amplitude of 340 W using a Branson 550 Sonicator. Cell debris was removed by centrifugation at 15000 RPM for 30 min, and the lysate was collected and passed through a 0.22 μm syringe filter. The clarified lysates were then mixed with 1 ml of nickel-NTA resin (Qiagen), preequilibrated with lysis buffer, and incubated on a rotisserie overnight a 4°C. After incubation, the mixture was poured into a glass gravity flow chromatography column (Bio-Rad), and the resin was washed with 50 mL of lysis buffer, followed by elution of the bound protein with 6 ml elution buffer (500 mM imidazole, 50 mM Tris-HCl, 300 mM NaCl, pH 8). Purified proteins were dialyzed at 4°C in 10-kDa molecular weight cutoff (MWCO) dialysis cassettes (Thermo Scientific). Dialysis conditions were as follows: 1 L buffer 1 (100 mM imidazole, 50 mM Tris-HCl, 300 mM NaCl, pH 8) for 3 hours, 1 L buffer 2 (25 mM imidazole, 50 mM Tris-HCl, 300 mM NaCl, pH 8) for 3 hours, 1 L buffer 3 (50 mM Tris-HCl, 300 mM NaCl, pH 8) overnight, and 1 L buffer 4 (50 mM Tris-HCl, 300 mM NaCl, pH 8) for 3 hours. Protein concentration was measured using a bicinchoninic acid (BCA) kit (Thermo Scientific). Protein purity was confirmed by resolving the purified proteins using sodium dodecyl sulfate polyacrylamide gel electrophoresis (SDS-PAGE), followed by staining gels with Coomassie blue. Purified proteins were stored in aliquots at -80°C until use.

RNA purification

S. aureus strains were struck onto fresh TSA plates and incubated overnight at 37°C. The next day, a single colony from the plate was inoculated into 3 mL of TSB in a 15 mL conical tube and incubated overnight at 37°C with shaking at 200 RPM. The following day, 250 μL of overnight culture was added to 25 mL of TSB + BCCAs in a 250 mL flask. Flasks were placed at 37°C with shaking at 200 RPM. For RNA sequencing, cultures were grown to late exponential phase (OD_{600} = 0.6) and for qPCR, strains were grown for 6 hours. 10 mL of each culture was removed and subsequently normalized to the OD_{600} of the strain with the lowest turbidity to ensure equivalent biomass. Cultures were centrifuged at 11000 RPM and washed twice with 15 mL PBS. Cell pellets were fixed in 15 mL of a 1:1 ethanol: acetone solution and stored at -80°C until RNA extraction.

RNA was subsequently purified using a RNeasy Micro kit (Qiagen) with an additional DNase digestion using Turbo DNase (Thermo). Briefly, bacterial pellets were thawed on ice and centrifuged for 15 minutes at 4000 RPM at 4°C to remove the ethanol:acetone solution. The pellet was air-dried and was washed twice with 500 µL of 1x PBS, followed by suspension of the washed pellet in 500 µL RLT buffer containing 10 µL/mL of β-mercaptoethanol. Suspensions were transferred to RNase-free Lysing Matrix B tubes (MP Biomedicals). Tubes were placed in a Fast Prep-24 5G (MP Biomedicals) bead disruption system, followed by two sequential cell lysis steps at 5.0 speed for 20 s and 4.5 speed for 20 s. Samples were kept on ice for 5 minutes between the two lysis steps. After lysis, samples were centrifuged at 10000 RPM for 20 min, and the supernatant was removed. 500 µL of RLT buffer was added to supernatants and the samples were vortexed, followed by addition of 500 µL of 100% ethanol to precipitate the RNA, followed by addition to a Qiagen RNeasy spin column. All remaining steps were performed according to the manufacturer's protocol. The RNA was eluted in 40 µL of DEPC-treated water. A second DNase digestion was conducted using 1 U Turbo DNase in solution (Invitrogen). The RNA was precipitated with 100% ethanol, added to an RNeasy spin column, washed 3 times in 450 µL wash buffer, and eluted in 50 µL of DEPC-treated water. The purified RNA was quantified via Nanodrop and RNA quality was assessed either by resolving the RNA on a 1.2% agarose/formaldehyde gel or by tape station analysis. RNA was stored at -80°C until cDNA synthesis or RNA sequencing.

qRT-PCR and RNA sequencing

For qRT-PCR, cDNA was first synthesized from 5 ng of purified RNA using the GoScript reverse transcription system following the manufacturer's protocol (Promega). Gene expression was assessed using SYBR Green (Bio-Rad) with the primer pairs listed in S2 Table on a QuantStudio 5 real-time PCR system. Fold change relative to housekeeping genes (*gyrB* or *sigA*) was calculated using the threshold cycle ($2^{-\Delta\Delta CT}$) method. RNA sequencing was completed by SeqCoast Genomics LLC. Briefly, samples were prepared for RNA sequencing using an Illumina Stranded Total RNA Prep Ligation with Ribo-Zero Plus Microbiome and IDT For Illumina Unique Dual Indexes. Sequencing was performed on the Illumina NextSeq2000 platform using a 300-cycle flow cell kit to produce 2 x 150 bp paired reads. Read demultiplexing, read trimming, and run analytics were performed using DRAGEN v4.2.7, an on-board analysis software on the NextSeq2000. The quality of the sequencing data was first assessed using FastQC v0.12.0. Next, all reads were trimmed and quality filtered using BBDuk v39.33 employing Illumina's adapters and the program's default settings. To remove any potential contaminating reads, the data were subjected to another round of quality processing. This was performed by splitting the reads and removing the contaminating reads, using BBSplit v39.33 and the reference genome of *S. aureus* subsp. *aureus* LAC. Due to the lower support of *S. aureus* subsp. *aureus* LAC reference genome (NC_007793) in the downstream analysis tools and databases, the processed reads were instead aligned to the reference genome of *S. aureus* subsp. *aureus* TCH1516 using Bowtie 2 v2.5.4. SAMtools v1.22.1 were then employed to convert the output files of Bowtie 2, followed by the counting of the mapped reads to the genomic features using featureCounts (from the Subread package, v2.1.1). Finally, differential expression analysis was conducted employing DESeq2 (version 1.40.2). Specifically, count matrices and sample metadata were imported with the DESeqDataSetFromMatrix function and normalized using DESeq2's median-of-ratios method. Afterwards, gene-wise dispersions were estimated, and a negative-binomial generalized linear model was fit to the counts using a design formula that included the condition factor. Statistical significance of coefficients was assessed using Wald test, and resulting p-values were adjusted for multiple testing by the Benjamini–Hochberg procedure to control the false discovery rate. Log2-fold changes were reported after effect-size shrinkage using the apeglm method, which provides a more stable and interpretable log-fold change estimate for ranking and visualization. Genes with adjusted p-value ≤ 0.05 were considered differentially expressed and consequently further analyzed. The data obtained from the differential expression analysis were further processed employing Pathview v1.48.0 for mapping and rendering the differentially expressed genes on relevant pathway graphs, clusterProfiler v4.16.0 for gene set enrichment analysis, and the individual genes manually parsed using Microsoft Excel (v2505). Normalized and mapped reads were visualized in Integrative Genomics Viewer (IGV).

Pathway analysis

Functional pathway enrichment was carried out using approaches similar to those described in a prior study [107]. In summary, the set of differentially expressed genes was assigned to biological pathways using the free Database for Annotation, Visualization and Integrated Discovery (DAVID) provided by NIAID/NIH [108,109]. Gene identifiers were converted to NCTC8325 locus tags in the SAOUHSC ortholog format with the help of AureoWiki, an online resource for *S. aureus*. The DAVID analysis returned unbiased functional annotations, suggested pathway groupings, and the number of genes associated with each pathway.

Growth curves

S. aureus strains were struck onto fresh TSA plates and incubated overnight at 37°C. The next day, a single colony from the plate was inoculated into 3 mL of TSB in a 15 mL conical tube and incubated overnight at 37°C with shaking at 200 RPM. Strains containing a Δ*lipL* mutation were grown with complete BCCAs overnight. The next day, strains were washed three times with 5 mL 1 x PBS and subcultured 1:100 (2 μL into 198 μL fresh TSB) in a flat-bottom 96-well plate (Costar) in technical triplicate. Strains were grown in TSB + BCCAs without the addition of sodium acetate. The plate was incubated in a Tecan Spark plate reader at 37°C with orbital shaking at 3 mm with a frequency of 180 RPM. Bacterial growth was monitored by measuring OD_{600} every hour for 24 hours.

Metabolite quantification

S. aureus strains were struck onto fresh TSA plates and incubated overnight at 37°C. The next day, a single colony from the plate was inoculated into 3 mL of TSB in a 15 mL conical tube and incubated overnight at 37°C with shaking at 200 RPM. The following day, 250 μL of overnight culture was added to 25 mL of TSB + BCCAs without sodium acetate in a 250 mL flask. Flasks were placed at 37°C with shaking at 200 RPM. For glucose, lactate, and formate measurements, 120 μL of culture was removed every hour for six hours. For acetate measurements, 60 μL of culture was collected every three hours for 12 hours, with a final collection at 24 hours. Samples were collected in 1.5 mL Eppendorf tubes at each time point and were centrifuged at 13000 RPM for 5 minutes. The cellfree supernatant was stored at -20°C until assaying metabolite levels. Acetate, glucose, formate, and D- and L-lactate concentrations were determined using kits purchased from R-Biopharm, according to the manufacturer's protocol with minor changes. Briefly, for all metabolites, 10 μL of each sample was incubated with 200 μL of reagent 1 for 3 minutes at room temperature in a flat-bottom 96-well plate (Costar) in technical triplicate. Following the primary incubation, 50 μL of reagent 2 was added to the reaction mixture and incubated for 15 minutes. A Tecan Spark plate reader was used to read the absorbance at 340 nm for each metabolite. Final calculations of metabolite concentrations were determined using a standard curve.

Intracellular pyruvate quantification

S. aureus strains were struck onto fresh TSA plates and incubated overnight at 37°C. The next day, a single colony from the plate was inoculated into 3 mL of TSB in a 15 mL conical tube and incubated overnight at 37°C with shaking at 200 RPM. The following day, 250 μL of overnight culture was added to 25 mL of TSB + BCCAs without sodium acetate in a 250 mL flask and placed at 37°C with shaking at 200 RPM. At 3 hours, cultures were normalized to the lowest optical density and 1 mL of normalized culture was added to 1.5 mL Eppendorf tubes, centrifuged at 10,000 RPM for two minutes, and washed twice with 1 mL 1x PBS. The bacterial suspension was mixed with pyruvate assay buffer from a commercial Pyruvate Assay Kit (Abcam) and transferred to screw cap microcentrifuge tubes (Fisher Scientific), which were preloaded with 250 μL of 0.1 mm glass beads (Electron Microscopy Sciences). Tubes were placed in a Fast Prep-24 5G (MP Biomedicals) bead disruption system, followed by two sequential cell lysis steps at 5.0 speed for 20 s and 4.5 speed for 20 s.

Samples were kept on ice for 5 minutes between the two lysis steps. After lysis, samples were centrifuged at 10000 RPM for 20 min, and 150 μL of the supernatant was collected and assayed according to the manufacturer's protocol.

Determination of protein lipoylation and densitometry

S. aureus strains were struck onto fresh TSA plates and incubated overnight at 37°C. The next day, a single colony from the plate was inoculated into 3 mL of TSB in a 15 mL conical tube and incubated overnight at 37°C with shaking at 200 RPM. Overnight cultures were diluted 1:100 in 10 mL fresh TSB in 50 mL conical tubes and incubated for 9 hours at 37°C with shaking at 200 RPM. Bacteria were centrifuged at 8000 RPM for 10 minutes, supernatants were removed, and cell pellets were resuspended in 1 mL 1x PBS. Bacterial suspensions were transferred to screw cap microcentrifuge tubes (Fisher Scientific), which were preloaded with 250 μL of 0.1 mm glass beads (Electron Microscopy Sciences). Tubes were placed in a Fast Prep-24 5G (MP Biomedicals) bead disruption system, followed by two sequential cell lysis steps at 5.0 speed for 20 s and at 4.5 speed for 20 s. Samples were kept on ice for 5 minutes between the two lysis steps. After lysis, samples were centrifuged at 11,000 RPM for 20 minutes, and 150 μL of the supernatant was collected in 1.5 mL centrifuge tubes containing 50 μL of 4X SDS sample buffer (0.2 M Tri-HCl, pH 6.8, 8% SDS, 5.5 M glycerol, 0.02 M EDTA, 0.6 M β-mercaptoethanol, and 6 mM Bromophenol blue). Samples were boiled for 10 minutes and stored at -20°C. Thawed cell lysates were loaded onto 15% polyacrylamide gels after normalizing to the original OD_{600} of the culture, followed by SDS-PAGE at 100 V for 2 hours. Resolved proteins were either stained with Coomassie blue or were transferred to 0.2 μm Immobilon polyvinylidene difluoride (PVDF) membranes (Millipore Sigma) at 20 V for 90 min. After transfer, membranes were incubated for 1 hour in Tris-buffered saline + 0.1% TWEEN 20 (Amresco) (TBST) supplemented with 5% bovine serum albumin (BSA) (GoldBio) and human IgG (Sigma) (1:2,000). Rabbit polyclonal α-lipoic acid antibody (Calbiochem) was added to the membranes at a 1:7,500 dilution followed by incubation overnight at 4°C. The following day, the membrane was washed three times for 10 minutes each in ~20 mL of TBST. Alkaline phosphatase (AP)-conjugated goat anti-rabbit IgG (H + L) (Invitrogen) was then added at a 1:5000 dilution in 5% BSA in TBST for 1 hour followed by three 10 minutes washes in ~20 mL of TBST. Membranes were developed with 5-bromo-4-chloro-3-indoyl-phosphate/nitro blue tetrazolium color development substrate (GoldBio). Densitometry analysis was performed using the ImageJ software on four independent experiments to determine the lipoylation of each band relative to the control band set at a value of 1.

Bacterial Adenylate Cyclase Two-Hybrid (BACTH) system

S. aureus pta and *lipL* genes were amplified (S2 Table) and cloned into plasmids pKT25 and pUT18c to generate translational fusions with the T25 or T18 domains of the *Bordetella pertussis* adenylate cyclase [51]. To clone the *lipL* gene into BACTH vectors, Sma1 and Pst1 restriction endonucleases were used for digestion and T4 DNA ligase for ligation. To clone the *pta* gene into BACTH vectors, Sma1 and Xba1 restriction endonucleases were used for digestion and T4 DNA ligase for ligation. The resulting plasmids were transformed into *E. coli* DH5α and plated on LB agar containing kanamycin (50 μg/mL) or ampicillin (100 μg/mL). Transformants containing the ligated amplicon were verified by PCR. The recombinant plasmids were purified from DH5α *E. coli* and then co-transformed into *E. coli* strain BTH101 and plated on LB agar containing both kanamycin (50 μg/mL) and ampicillin (100 μg/mL). To assay for protein-protein interactions, single colonies were inoculated into 3 mL LB in 15 mL conical tubes containing both antibiotics overnight. The following day, strains were diluted 1:100 and grown for 6 hours at 30°C with shaking at 200 RPM. Upon reaching late-exponential phase (~6 hours), 10 μL culture was spotted on LB agar containing IPTG (0.5 mM), ampicillin (100 μg/mL), kanamycin (50 μg/mL), and X-Gal (100 μg/mL). Plates were incubated for 24 hours at 30°C to allow for bacterial growth. An interaction was defined by the presence of blue color relative to positive and negative controls. The negative control, empty BACTH plasmids, yield colorless colonies. The positive control was BTH101 co-transformed with pKT25-zip and pUT18C-zip, plasmids expressing leucine zipper - adenylate cyclase fusions that dimerize to yield blue colonies.

β-galactosidase activity was quantified based on previous methods [110]. Briefly, single colonies were inoculated in 3 mL LB in 15 mL conical tubes containing both antibiotics overnight. The next day, the cultures were diluted 1:100 in 3 mL LB and grown for 6 hours with IPTG (0.5 mM), ampicillin (100 µg/mL) and kanamycin (50 µg/mL) with shaking at 200 RPM at 30°C until reaching an OD_{600} ~ 1.0. Strains were OD normalized and 100 µL of each sample was added to a 96-well plate (Costar) in triplicate. The cultures were centrifuged at 4000 RPM, and the bacterial pellets were resuspended in 80 µL of Z-buffer (60 mM $Na_2HPO_4 \cdot 7H_2O$, 40 mM $NaH_2PO_4 \cdot H_2O$, 10 mM KCl, 1 mM $MgSO_4$, 50 mM β-mercaptoethanol, pH 7). The resuspended cells were then permeabilized with 10 µL of 0.1% SDS and 10 µL of chloroform. Plates were centrifuged at 4000 RPM, the supernatant was transferred to a new 96-well plate, mixed with 20 µL ONPG (4 mg/mL), and the reaction was allowed to proceed for 15–30 min. Reactions were stopped by adding 30 µL 1 M Na_2CO_3 and absorbance was measured at 420 nm. The negative control, BTH101 carrying the empty pKT25 and pUT18c plasmids, and the positive control, BTH101 containing pKT25-zip and pUT18c-zip plasmids, are described above. β-galactosidase activity is reflected relative to the negative and positive controls, with the negative control assigned an activity value of 0 in arbitrary units and positive control assigned an activity of 1000 arbitrary units.

Microscale Thermophoresis (MST)

Experiments were performed as described previously with minor modifications [111]. Briefly, pure recombinant Pta was labeled with a NanoTemper 2nd Generation Red N-hydroxysuccinimide (NHS) Dye (Nanotemper Technologies). For protein labeling, Pta was first buffer exchanged into buffer M (20 mM MES, 100 mM NaCl, 10% glycerol, pH 7.5) to remove incompatible buffer components. 10 µM Pta was mixed with 3X excess of the Red NHS dye (300 µM) and incubated in the dark for 30 minutes for the dye to react with the protein. The protein-dye mixture was passed through a gel filtration column (Column B, Nanotemper Technologies) to remove unreacted and excess dye. For a 1:1 protein-dye conjugate, the degree of label (DOL) of the protein was determined using the formula: A650/195,000/M/cm × concentration of labeled protein. A650; absorbance at 650 nm, and 195,000/M/cm is the molar absorbance of the Red NHS dye. A 1:1 protein dye ratio occurred at a DOL value between 0.6 and 1. After labeling, protein samples were aliquoted into 10µL volumes, flash frozen using liquid nitrogen, and stored at -80°C until used. For microscale thermophoresis experiments, the unlabeled protein partner was serially diluted in low-binding tubes with buffer M supplemented with 1 mM BME and 0.05% Tween 20, pH 7.5, and titrated against 20 nM of the labeled proteins. The protein mixtures were loaded into standard Monolith NT.115 capillary tubes, and thermophoresis was determined using the following parameters: 20% excitation power and high MST Power at 25°C.

Thermophoresis results were analyzed using the PALMIST [112] and GUSSI [112] analysis pipeline. Briefly, data from the MST software (Mo. Control v1.6.1) were imported into the PALMIST software and a preset T-jump (TJ) was applied to the data using a 1:1 binding model with a 95% confidence interval. Datasets were examined for kinetic effects to ensure that thermophoresis achieved equilibrium. After data analysis, GUSSI was used for figure rendering. The top panel in Fig 4B indicates the normalized fluorescence data points, the middle panel represents the binding curve, and the lower panel represents the residuals (a plot between the fitted line and the data). Error bars indicate the standard deviation of four independent replicates.

Murine infection models

S. aureus strains were struck onto fresh TSA plates and incubated overnight at 37°C with shaking at 200 RPM. The next day, a single colony from the plate was inoculated into 3 mL of TSB in a 15 mL conical tube and incubated overnight at 37°C with shaking at 200 RPM. The overnight cultures were diluted 1:100 in 3 mL fresh TSB and grown for 3 h at 37°C with shaking at 200 RPM. Bacteria were pelleted by centrifugation at 3900 RPM at 4°C for 5 minutes. The cells were washed three times with 5 mL of 1X PBS. Bacterial cell suspensions were normalized to an OD_{600} of ~0.32 (~1×10^8 CFU/mL), and serial dilutions were plated onto TSA plates to enumerate CFU and ensure accuracy

of inocula. Six-week-old female Swiss Webster mice (Envigo) were anesthetized with ketamine/xylazine (100/10 mg/kg) via intraperitoneal injection. For systemic infections, 100 μL of OD-normalized *S. aureus* in PBS (~1.0×10^7 CFU) was injected directly into the bloodstream via the retro-orbital sinus. Mice were monitored daily. At 96 h postinfection, mice were euthanized, and the kidneys were recovered, homogenized, serially diluted, dilutions were plated onto tryptic soy agar followed by incubation at 37°C overnight to enumerate CFU. For skin infections, bacterial suspensions (~1.0×10^7 CFU) were injected intradermally into anesthetized mice on each side of a shaved flank region. Mice were monitored daily. At 72 h postinfection, mice were euthanized, and abscesses were excised, homogenized, and plated to enumerate CFU.

Statistical analysis

For *in vitro* growth assays, western blots, and metabolite quantifications, data shown are representative of at least three independent experiments conducted in triplicate. qRT-PCR experiments included two independent biological replicates conducted in technical triplicate with all data shown. All animal studies include combined data from at least two independent experiments. Statistical analyses were conducted using GraphPad Prism version 10. Comparisons among three or more groups were conducted using either one-way ANOVA followed by Tukey's post hoc test or Kruskal–Wallis test followed by Dunn's post-test, whereas differences between two groups were analyzed with the Mann–Whitney test. A *p*-value below 0.05 was considered statistically significant. Statistical significance is indicated as follows: *, $p < 0.05$; **, $p < 0.01$; ***, $p < 0.001$; ****, $p < 0.0001$.

Supporting information

S1 Fig. Growth of WT, Δ*pta*, Δ*lipL*, Δ*pta* + *pta*, and Δ*lipL* + *lipL* strains in TSB and TSB + BCCAs [10 mM isobutyric acid (IB), 9 mM 2-methylbutyric acid (2MB), 9 mM isovaleric acid (IV) + 10 mM sodium acetate (NaAc)] containing 0 mM glucose (A) or 3.5 mM glucose (B). (C) Quantification of glucose, acetate, lactate and formate from WT, Δ*pta*, Δ*lipL*, Δ*pta* + *pta*, and Δ*lipL* + *lipL* culture supernatants over time in TSB with 0 mM glucose. (D) Quantification of glucose, acetate, lactate and formate from WT, Δ*pta*, Δ*lipL*, Δ*pta* + *pta*, and Δ*lipL* + *lipL* culture supernatants over time in TSB with 3.5 mM glucose. Each metabolite quantification assay is representative of three independent experiments, with each time-point measured in technical triplicate. Errors bars indicate standard deviation from the mean.
(TIF)

S2 Fig. (A) qRT-PCR analysis of RNA extracted from WT, Δ*pta,* and Δ*lipL* strains at 3 and 6 hours of growth. qRT-PCR experiments were conducted with two biological replicates in technical triplicate. (B) Top - schematic of the *cid* operon. Bottom - normalized and mapped transcript reads across the *cid* operon in WT and Δ*lipL* strains. (B) Bacterial adenylate cyclase two-hybrid (BACTH) assay was used to test interactions between N-terminal-tagged Pta with LipL and C-terminal-tagged Pta with E2-PDH. Graph displays β-galactosidase activity (Miller Units) from *E. coli* BTH101 co-transformed with pUT18-*pta* and pKT25-*lipL*, pUT18C-*pta* and pKT25-*pdhC*, pUT18C-zip and pKT25-zip (positive control – PC) or pUT18C and pKT25 vectors (negative control – NC). ****, $p < 0.0001$ by one-way ANOVA with Tukey's post hoc test. Cultures of *E. coli* BTH101strains co-transformed with the aforementioned plasmid combinations were also spotted on LB agar plates with X-Gal as an indicator. Data are representative of at least three independent experiments. (D) AlphaFold3 prediction of the interaction between Pta (blue) and E2-PDH (green). pTM (0.48) and an ipTM (0.2) are displayed graphically to the right of the model.
(TIF)

S1 Table. Strains used in this study.
(PDF)

S2 Table. List of Oligonucleotides used in this study.
(PDF)

S3 Table. Significant differentially expressed genes (*ΔlipL* mutant compared to WT *S. aureus*).
(XLSX)

S4 Table. NIH DAVID pathway analysis.
(XLSX)

Acknowledgments

We thank Christoper Curme for his assistance with the pathway analysis. We are also grateful to the members of the Alonzo laboratory for their valuable discussions and constructive feedback on the manuscript.

Author contributions

Conceptualization: Reginald A. Woods, Francis Alonzo III.

Data curation: Reginald A. Woods, Francis Alonzo III.

Formal analysis: Reginald A. Woods, Charles Agbavor, Luka Svet, Manaar Yousef.

Funding acquisition: Victor J. Torres, Laty A. Cahoon, Francis Alonzo III.

Investigation: Reginald A. Woods, Iván C. Acosta, Zachary J. Resko, Charles Agbavor, Manaar Yousef, Wei Ping Teoh.

Methodology: Reginald A. Woods, Iván C. Acosta, Zachary J. Resko, Charles Agbavor, Luka Svet, Manaar Yousef, Wei Ping Teoh, Laty A. Cahoon.

Project administration: Francis Alonzo III.

Resources: Victor J. Torres.

Supervision: Victor J. Torres, Laty A. Cahoon, Francis Alonzo III.

Visualization: Luka Svet.

Writing – original draft: Reginald A. Woods, Francis Alonzo III.

Writing – review & editing: Reginald A. Woods, Iván C. Acosta, Zachary J. Resko, Charles Agbavor, Luka Svet, Victor J. Torres, Laty A. Cahoon, Francis Alonzo III.

References

1. Zorzoli A, Grayczyk JP, Alonzo F 3rd. Staphylococcus aureus tissue infection during sepsis is supported by differential use of bacterial or host-derived lipoic acid. PLoS Pathog. 2016;12(10):e1005933. https://doi.org/10.1371/journal.ppat.1005933 PMID: 27701474
2. Juttukonda LJ, Berends ETM, Zackular JP, Moore JL, Stier MT, Zhang Y, et al. Dietary manganese promotes staphylococcal infection of the heart. Cell Host Microbe. 2017;22(4):531-542.e8. https://doi.org/10.1016/j.chom.2017.08.009 PMID: 28943329
3. Balasubramanian D, Harper L, Shopsin B, Torres VJ. Staphylococcus aureus pathogenesis in diverse host environments. Pathog Dis. 2017;75(1):ftx005. https://doi.org/10.1093/femspd/ftx005 PMID: 28104617
4. Richardson AR. Virulence and metabolism. Microbiol Spectr. 2019;7(2):7.2.39. https://doi.org/10.1128/microbiolspec.GPP3-0011-2018 PMID: 31025624
5. Teoh WP, Chen X, Laczkovich I, Alonzo F III. Staphylococcus aureus adapts to the host nutritional landscape to overcome tissue-specific branched-chain fatty acid requirement. Proc Natl Acad Sci USA. 2021;118(13):e2022720118. https://doi.org/10.1073/pnas.2022720118
6. Chen X, Teoh WP, Stock MR, Resko ZJ, Iii FA. Branched chain fatty acid synthesis drives tissue-specific innate immune response and infection dynamics of Staphylococcus aureus. PLOS Pathogens. 2021;17(9):e1009930. https://doi.org/10.1371/journal.ppat.1009930
7. Richardson AR, Somerville GA, Sonenshein AL. Regulating the intersection of metabolism and pathogenesis in gram-positive bacteria. Microbiol Spectr. 2015;3(3):3.3.11. https://doi.org/10.1128/microbiolspec.MBP-0004-2014 PMID: 26185086

8. Tong SYC, Davis JS, Eichenberger E, Holland TL, Fowler VG Jr. Staphylococcus aureus infections: epidemiology, pathophysiology, clinical manifestations, and management. Clin Microbiol Rev. 2015;28(3):603–61. https://doi.org/10.1128/CMR.00134-14 PMID: 26016486
9. Naghavi M, Vollset SE, Ikuta KS, Swetschinski LR, Gray AP, Wool EE, et al. Global burden of bacterial antimicrobial resistance 1990–2021: a systematic analysis with forecasts to 2050. The Lancet. 2024;404(10459):1199–226. https://doi.org/10.1016/S0140-6736(24)01867-1 PMID: 39299261
10. Chen X, Alonzo F. Bacterial lipolysis of immune-activating ligands promotes evasion of innate defenses. Proc Natl Acad Sci U S A. 2019;116(9):3764–73. https://doi.org/10.1073/pnas.1817248116 PMID: 30755523
11. Watson SP, Clements MO, Foster SJ. Characterization of the starvation-survival response of Staphylococcus aureus. J Bacteriol. 1998;180(7):1750–8. https://doi.org/10.1128/JB.180.7.1750-1758.1998 PMID: 9537371
12. Jenul C, Horswill AR. Regulation of Staphylococcus aureus virulence. Microbiol Spectr. 2019;7(2):10.1128/microbiolspec.gpp3-0031–2018. https://doi.org/10.1128/microbiolspec.GPP3-0031-2018 PMID: 30953424
13. Tuganova A, Popov KM. Role of protein–protein interactions in the regulation of pyruvate dehydrogenase kinase activity. Biochem J. 2005;387(1):147–53. https://doi.org/10.1042/BJ20040805 PMID: 15504108
14. Durek P, Walther D. The integrated analysis of metabolic and protein interaction networks reveals novel molecular organizing principles. BMC Syst Biol. 2008;2:100. https://doi.org/10.1186/1752-0509-2-100 PMID: 19032748
15. Aboulwafa M, Zhang Z, Saier MH Jr. Protein:Protein interactions in the cytoplasmic membrane apparently influencing sugar transport and phosphorylation activities of the e. coli phosphotransferase system. PLoS One. 2019;14(11):e0219332. https://doi.org/10.1371/journal.pone.0219332 PMID: 31751341
16. Burckhardt RM, Buckner BA, Escalante-Semerena JC. Staphylococcus aureus modulates the activity of acetyl-Coenzyme A synthetase (Acs) by sirtuin-dependent reversible lysine acetylation. Mol Microbiol. 2019;112(2):588–604. https://doi.org/10.1111/mmi.14276 PMID: 31099918
17. Kelliher JL, Radin JN, Grim KP, Párraga Solórzano PK, Degnan PH, Kehl-Fie TE. Acquisition of the phosphate transporter NptA enhances Staphylococcus aureus pathogenesis by improving phosphate uptake in divergent environments. Infect Immun. 2017;86(1):e00631-17. https://doi.org/10.1128/IAI.00631-17 PMID: 29084897
18. Kelliher JL, Radin JN, Kehl-Fie TE. PhoPR contributes to Staphylococcus aureus growth during phosphate starvation and pathogenesis in an environment-specific manner. Infect Immun. 2018;86(10):e00371-18. https://doi.org/10.1128/IAI.00371-18 PMID: 30061377
19. Vitko NP, Grosser MR, Khatri D, Lance TR, Richardson AR. Expanded glucose import capability affords Staphylococcus aureus optimized glycolytic flux during infection. mBio. 2016;7(3):10.1128/mbio.00296-16. https://doi.org/10.1128/mbio.00296-16
20. Kaiser JC, Omer S, Sheldon JR, Welch I, Heinrichs DE. Role of BrnQ1 and BrnQ2 in branched-chain amino acid transport and virulence in Staphylococcus aureus. Infect Immun. 2015;83(3):1019–29. https://doi.org/10.1128/IAI.02542-14 PMID: 25547798
21. Frank MW, Yao J, Batte JL, Gullett JM, Subramanian C, Rosch JW. Host fatty acid utilization by Staphylococcus aureus at the infection site. mBio. 2020;11(3):e00920-20. https://doi.org/10.1128/mBio.00920-20
22. Thomsen IP, Liu GY. Targeting fundamental pathways to disrupt Staphylococcus aureus survival: clinical implications of recent discoveries. JCI Insight. 2018;3(5):e98216. https://doi.org/10.1172/jci.insight.98216 PMID: 29515041
23. Martin N, Lombardía E, Altabe SG, de Mendoza D, Mansilla MC. A lipA (yutB) mutant, encoding lipoic acid synthase, provides insight into the interplay between branched-chain and unsaturated fatty acid biosynthesis in Bacillus subtilis. J Bacteriol. 2009;191(24):7447–55. https://doi.org/10.1128/JB.01160-09 PMID: 19820084
24. Christensen QH, Cronan JE. Lipoic acid synthesis: a new family of octanoyltransferases generally annotated as lipoate protein ligases. Biochemistry. 2010;49(46):10024–36. https://doi.org/10.1021/bi101215f PMID: 20882995
25. Cronan JE. Assembly of lipoic acid on its cognate enzymes: an extraordinary and essential biosynthetic pathway. Microbiol Mol Biol Rev. 2016;80(2):429–50. https://doi.org/10.1128/MMBR.00073-15 PMID: 27074917
26. Teoh WP, Resko ZJ, Flury S, Alonzo F 3rd. Dynamic relay of protein-bound lipoic acid in Staphylococcus aureus. J Bacteriol. 2019;201(22):e00446-19. https://doi.org/10.1128/JB.00446-19 PMID: 31451544
27. Wolfe AJ. The acetate switch. MMBR. 2005;69(1):12–50. https://doi.org/10.1128/MMBR.69.1.12-50.2005
28. Sadykov MR, Thomas VC, Marshall DD, Wenstrom CJ, Moormeier DE, Widhelm TJ, et al. Inactivation of the Pta-AckA pathway causes cell death in Staphylococcus aureus. J Bacteriol. 2013;195(13):3035–44. https://doi.org/10.1128/JB.00042-13 PMID: 23625849
29. Schütze A, Benndorf D, Püttker S, Kohrs F, Bettenbrock K. The impact of ackA, pta, and ackA-pta mutations on growth, gene expression and protein acetylation in Escherichia coli K-12. Front Microbiol [Internet]. 2020 [cited 2023 May 9];11. Available from: https://www.frontiersin.org/articles/10.3389/fmicb.2020.00233
30. Shahreen N, Ahn J, Alsiyabi A, Chowdhury NB, Shinde D, Chaudhari SS, et al. A thermodynamic bottleneck in the TCA cycle contributes to acetate overflow in Staphylococcus aureus. mSphere. 2025;10(1):e0088324. https://doi.org/10.1128/msphere.00883-24 PMID: 39745366
31. Troitzsch A, Loi VV, Methling K, Zühlke D, Lalk M, Riedel K, et al. Carbon source-dependent reprogramming of anaerobic metabolism in Staphylococcus aureus. J Bacteriol. 2021;203(8):e00639-20. https://doi.org/10.1128/JB.00639-20 PMID: 33526614
32. Fuchs S, Pané-Farré J, Kohler C, Hecker M, Engelmann S. Anaerobic gene expression in Staphylococcus aureus. J Bacteriol. 2007;189(11):4275–89. https://doi.org/10.1128/JB.00081-07 PMID: 17384184

33. Yang SJ, Rice KC, Brown RJ, Patton TG, Liou LE, Park YH, et al. A lysR-type regulator, cidR, is required for induction of the Staphylococcus aureus cidABC operon. J Bacteriol. 2005;187(17):5893–900. https://doi.org/10.1128/JB.187.17.5893-5900.2005 PMID: 16109930
34. Rice KC, Nelson JB, Patton TG, Yang S-J, Bayles KW. Acetic acid induces expression of the Staphylococcus aureus cidABC and lrgAB murein hydrolase regulator operons. J Bacteriol. 2005;187(3):813–21. https://doi.org/10.1128/JB.187.3.813-821.2005 PMID: 15659658
35. Rice KC, Mann EE, Endres JL, Weiss EC, Cassat JE, Smeltzer MS, et al. The cidA murein hydrolase regulator contributes to DNA release and biofilm development in Staphylococcus aureus. Proc Natl Acad Sci U S A. 2007;104(19):8113–8. https://doi.org/10.1073/pnas.0610226104 PMID: 17452642
36. Thomas VC, Sadykov MR, Chaudhari SS, Jones J, Endres JL, Widhelm TJ, et al. A central role for carbon-overflow pathways in the modulation of bacterial cell death. PLoS Pathog. 2014;10(6):e1004205. https://doi.org/10.1371/journal.ppat.1004205 PMID: 24945831
37. Patton TG, Rice KC, Foster MK, Bayles KW. The Staphylococcus aureus cidC gene encodes a pyruvate oxidase that affects acetate metabolism and cell death in stationary phase. Mol Microbiol. 2005;56(6):1664–74. https://doi.org/10.1111/j.1365-2958.2005.04653.x PMID: 15916614
38. Endres JL, Chaudhari SS, Zhang X, Prahlad J, Wang SQ, Foley LA. The Staphylococcus aureus CidA and LrgA proteins are functional holins involved in the transport of by-products of carbohydrate metabolism. mBio. 2021;13(1):e0282721. https://doi.org/10.1128/mbio.02827-21
39. Chaudhari SS, Thomas VC, Sadykov MR, Bose JL, Ahn DJ, Zimmerman MC, et al. The LysR-type transcriptional regulator, CidR, regulates stationary phase cell death in Staphylococcus aureus. Mol Microbiol. 2016;101(6):942–53. https://doi.org/10.1111/mmi.13433 PMID: 27253847
40. Thurlow LR, Stephens AC, Hurley KE, Richardson AR. Lack of nutritional immunity in diabetic skin infections promotes Staphylococcus aureus virulence. Sci Adv. 2020;6(46):eabc5569. https://doi.org/10.1126/sciadv.abc5569 PMID: 33188027
41. Spalding MD, Prigge ST. Lipoic acid metabolism in microbial pathogens. Microbiol Mol Biol Rev. 2010;74(2):200–28. https://doi.org/10.1128/MMBR.00008-10 PMID: 20508247
42. Choueiry F, Xu R, Zhu J. Adaptive metabolism of Staphylococcus aureus revealed by untargeted metabolomics. J Proteome Res. 2022;21(2):470–81. https://doi.org/10.1021/acs.jproteome.1c00797 PMID: 35043624
43. Vitko NP, Spahich NA, Richardson AR. Glycolytic dependency of high-level nitric oxide resistance and virulence in Staphylococcus aureus. mBio. 2015;6(2):e00045-15. https://doi.org/10.1128/mBio.00045-15
44. Somerville GA, Chaussee MS, Morgan CI, Fitzgerald JR, Dorward DW, Reitzer LJ, et al. Staphylococcus aureus aconitase inactivation unexpectedly inhibits post-exponential-phase growth and enhances stationary-phase survival. Infect Immun. 2002;70(11):6373–82. https://doi.org/10.1128/IAI.70.11.6373-6382.2002 PMID: 12379717
45. Halsey CR, Lei S, Wax JK, Lehman MK, Nuxoll AS, Steinke L. Amino acid catabolism in Staphylococcus aureus and the function of carbon catabolite repression. mBio. 2017;8(1):e01434-16. https://doi.org/10.1128/mBio.01434-16 PMID: 28196956
46. Monk IR, Shah IM, Xu M, Tan MW, Foster TJ. Transforming the untransformable: application of direct transformation to manipulate genetically Staphylococcus aureus and Staphylococcus epidermidis. mBio. 2012;3(2):e00277-11. https://doi.org/10.1128/mBio.00277-11 PMID: 22434850
47. Monk IR, Stinear TP. From cloning to mutant in 5 days: rapid allelic exchange in Staphylococcus aureus. Access Microbiol. 2021;3(2):000193. https://doi.org/10.1099/acmi.0.000193 PMID: 34151146
48. Chen J, Yoong P, Ram G, Torres VJ, Novick RP. Single-copy vectors for integration at the SaPI1 attachment site for Staphylococcus aureus. Plasmid. 2014;76:1–7. https://doi.org/10.1016/j.plasmid.2014.08.001 PMID: 25192956
49. Somerville GA, Saïd-Salim B, Wickman JM, Raffel SJ, Kreiswirth BN, Musser JM. Correlation of acetate catabolism and growth yield in Staphylococcus aureus: implications for host-pathogen interactions. Infect Immun. 2003;71(8):4724–32. https://doi.org/10.1128/IAI.71.8.4724-4732.2003 PMID: 12874354
50. Schlag S, Fuchs S, Nerz C, Gaupp R, Engelmann S, Liebeke M, et al. Characterization of the oxygen-responsive NreABC regulon of Staphylococcus aureus. J Bacteriol. 2008;190(23):7847–58. https://doi.org/10.1128/JB.00905-08 PMID: 18820014
51. Karimova G, Pidoux J, Ullmann A, Ladant D. A bacterial two-hybrid system based on a reconstituted signal transduction pathway. Proc Natl Acad Sci U S A. 1998;95(10):5752–6. https://doi.org/10.1073/pnas.95.10.5752 PMID: 9576956
52. Battesti A, Bouveret E. The bacterial two-hybrid system based on adenylate cyclase reconstitution in Escherichia coli. Methods. 2012;58(4):325–34. https://doi.org/10.1016/j.ymeth.2012.07.018 PMID: 22841567
53. Won HI, Watson SM, Ahn J-S, Endres JL, Bayles KW, Sadykov MR. Inactivation of the Pta-AckA pathway impairs fitness of Bacillus anthracis during overflow metabolism. J Bacteriol. 2021;203(9):e00660-20. https://doi.org/10.1128/JB.00660-20 PMID: 33593944
54. Torres VJ, Benson MA, Voyich JM. Staphylococcus aureus pathogenesis and virulence factor regulation. In: Regulation of bacterial virulence [Internet]. John Wiley & Sons, Ltd; 2012 [cited 2026 Feb 26]. p. 58–78. Available from: https://onlinelibrary.wiley.com/doi/abs/10.1128/9781555818524.ch4 https://doi.org/10.1128/9781555818524.ch4
55. Alonzo F 3rd, Torres VJ. The bicomponent pore-forming leucocidins of Staphylococcus aureus. Microbiol Mol Biol Rev. 2014;78(2):199–230. https://doi.org/10.1128/MMBR.00055-13 PMID: 24847020
56. Benson MA, Lilo S, Wasserman GA, Thoendel M, Smith A, Horswill AR, et al. Staphylococcus aureus regulates the expression and production of the staphylococcal superantigen-like secreted proteins in a Rot-dependent manner. Mol Microbiol. 2011;81(3):659–75. https://doi.org/10.1111/j.1365-2958.2011.07720.x PMID: 21651625

57. Anil A, Braza RED, Liu B, Altouma V, Adedeji C, Welling A, et al. Staphylococcus aureus exhibits spatiotemporal heterogeneity in Sae activity during kidney abscess development. mBio. 2025;16(12):e0204325. https://doi.org/10.1128/mbio.02043-25 PMID: 41369255

58. Stock MR, Fang L, Johnson KR, Cosgriff C, Teoh WP, Alonzo F 3rd. Characterization of MroQ-dependent maturation and export of the Staphylococcus aureus accessory gene regulatory system autoinducing peptide. Infect Immun. 2022;90(10):e0026322. https://doi.org/10.1128/iai.00263-22 PMID: 36073934

59. Jordan SW, Cronan JE Jr. The Escherichia coli lipB gene encodes lipoyl (octanoyl)-acyl carrier protein:protein transferase. J Bacteriol. 2003;185(5):1582–9. https://doi.org/10.1128/JB.185.5.1582-1589.2003 PMID: 12591875

60. Miller JR, Busby RW, Jordan SW, Cheek J, Henshaw TF, Ashley GW, et al. Escherichia coli LipA is a lipoyl synthase: in vitro biosynthesis of lipoylated pyruvate dehydrogenase complex from octanoyl-acyl carrier protein. Biochemistry. 2000;39(49):15166–78. https://doi.org/10.1021/bi002060n PMID: 11106496

61. Martin N, Christensen QH, Mansilla MC, Cronan JE, de Mendoza D. A novel two-gene requirement for the octanoyltransfer reaction of Bacillus subtilis lipoic acid biosynthesis. Mol Microbiol. 2011;80(2):335–49. https://doi.org/10.1111/j.1365-2958.2011.07597.x PMID: 21338420

62. Christensen QH, Martin N, Mansilla MC, de Mendoza D, Cronan JE. A novel amidotransferase required for lipoic acid cofactor assembly in Bacillus subtilis. Mol Microbiol. 2011;80(2):350–63. https://doi.org/10.1111/j.1365-2958.2011.07598.x PMID: 21338421

63. Freiberg C, Brunner NA, Schiffer G, Lampe T, Pohlmann J, Brands M, et al. Identification and characterization of the first class of potent bacterial acetyl-CoA carboxylase inhibitors with antibacterial activity. J Biol Chem. 2004;279(25):26066–73. https://doi.org/10.1074/jbc.M402989200 PMID: 15066985

64. Singh VK, Sirobhushanam S, Ring RP, Singh S, Gatto C, Wilkinson BJ. Roles of pyruvate dehydrogenase and branched-chain α-keto acid dehydrogenase in branched-chain membrane fatty acid levels and associated functions in Staphylococcus aureus. J Med Microbiol. 2018;67(4):570–8. https://doi.org/10.1099/jmm.0.000707 PMID: 29498620

65. Satiaputra J, Eijkelkamp BA, McDevitt CA, Shearwin KE, Booker GW, Polyak SW. Biotin-mediated growth and gene expression in Staphylococcus aureus is highly responsive to environmental biotin. Appl Microbiol Biotechnol. 2018;102(8):3793–803. https://doi.org/10.1007/s00253-018-8866-z PMID: 29508030

66. Laczkovich I, Teoh WP, Flury S, Grayczyk JP, Zorzoli A, Alonzo F 3rd. Increased flexibility in the use of exogenous lipoic acid by *Staphylococcus aureus*. Mol Microbiol. 2018;109(2):150–68. https://doi.org/10.1111/mmi.13970 PMID: 29660187

67. Gaupp R, Schlag S, Liebeke M, Lalk M, Götz F. Advantage of upregulation of succinate dehydrogenase in Staphylococcus aureus biofilms. J Bacteriol. 2010;192(9):2385–94. https://doi.org/10.1128/JB.01472-09 PMID: 20207757

68. Hammer ND, Reniere ML, Cassat JE, Zhang Y, Hirsch AO, Indriati Hood M, et al. Two heme-dependent terminal oxidases power Staphylococcus aureus organ-specific colonization of the vertebrate host. mBio. 2013;4(4):e00241-13. https://doi.org/10.1128/mBio.00241-13 PMID: 23900169

69. Xu T, Han J, Zhang J, Chen J, Wu N, Zhang W, et al. Absence of protoheme IX farnesyltransferase CtaB causes virulence attenuation but enhances pigment production and persister survival in MRSA. Front Microbiol. 2016;7:1625. https://doi.org/10.3389/fmicb.2016.01625 PMID: 27822202

70. Hammer ND, Schurig-Briccio LA, Gerdes SY, Gennis RB, Skaar EP. CtaM is required for menaquinol oxidase aa3 function in Staphylococcus aureus. mBio. 2016;7(4):e00823-16. https://doi.org/10.1128/mBio.00823-16

71. Chaudhari SS, Kim M, Lei S, Razvi F, Alqarzaee AA, Hutfless EH. Nitrite derived from endogenous bacterial nitric oxide synthase activity promotes aerobic respiration. mBio. 2017;8(4):e00887-17. https://doi.org/10.1128/mBio.00887-17 PMID: 28765220

72. Rice KC, Patton T, Yang S-J, Dumoulin A, Bischoff M, Bayles KW. Transcription of the Staphylococcus aureus cid and lrg murein hydrolase regulators is affected by sigma factor B. J Bacteriol. 2004;186(10):3029–37. https://doi.org/10.1128/JB.186.10.3029-3037.2004 PMID: 15126464

73. Sadykov MR, Windham IH, Widhelm TJ, Yajjala VK, Watson SM, Endres JL, et al. CidR and CcpA synergistically regulate Staphylococcus aureus cidABC expression. J Bacteriol. 2019;201(23):e00371-19. https://doi.org/10.1128/JB.00371-19 PMID: 31501288

74. Wolfe AJ, Chang D-E, Walker JD, Seitz-Partridge JE, Vidaurri MD, Lange CF, et al. Evidence that acetyl phosphate functions as a global signal during biofilm development. Mol Microbiol. 2003;48(4):977–88. https://doi.org/10.1046/j.1365-2958.2003.03457.x PMID: 12753190

75. Kim JN, Ahn S-J, Burne RA. Genetics and physiology of acetate metabolism by the Pta-Ack pathway of Streptococcus mutans. Appl Environ Microbiol. 2015;81(15):5015–25. https://doi.org/10.1128/AEM.01160-15 PMID: 25979891

76. Presecan-Siedel E, Galinier A, Longin R, Deutscher J, Danchin A, Glaser P, et al. Catabolite regulation of the pta gene as part of carbon flow pathways in Bacillus subtilis. J Bacteriol. 1999;181(22):6889–97. https://doi.org/10.1128/JB.181.22.6889-6897.1999 PMID: 10559153

77. Huthmacher C, Gille C, Holzhütter H-G. A computational analysis of protein interactions in metabolic networks reveals novel enzyme pairs potentially involved in metabolic channeling. J Theor Biol. 2008;252(3):456–64. https://doi.org/10.1016/j.jtbi.2007.09.042 PMID: 17988690

78. Xu QS, Jancarik J, Lou Y, Kuznetsova K, Yakunin AF, Yokota H, et al. Crystal structures of a phosphotransacetylase from Bacillus subtilis and its complex with acetyl phosphate. J Struct Funct Genomics. 2005;6(4):269–79. https://doi.org/10.1007/s10969-005-9001-9 PMID: 16283428

79. Iyer PP, Lawrence SH, Luther KB, Rajashankar KR, Yennawar HP, Ferry JG, et al. Crystal structure of phosphotransacetylase from the methanogenic archaeon Methanosarcina thermophila. Structure. 2004;12(4):559–67. https://doi.org/10.1016/j.str.2004.03.007 PMID: 15062079

80. Xu QS, Shin D-H, Pufan R, Yokota H, Kim R, Kim S-H. Crystal structure of a phosphotransacetylase from Streptococcus pyogenes. Proteins. 2004;55(2):479–81. https://doi.org/10.1002/prot.20039 PMID: 15048838

81. Rado TA, Hoch JA. Phosphotransacetylase from Bacillus subtilis: purification and physiological studies. Biochim Biophys Acta. 1973;321(1):114–25. https://doi.org/10.1016/0005-2744(73)90065-x PMID: 4201530

82. Brinsmade SR, Escalante-Semerena JC. In vivo and in vitro analyses of single-amino acid variants of the Salmonella enterica phosphotransacetylase enzyme provide insights into the function of its N-terminal domain. J Biol Chem. 2007;282(17):12629–40. https://doi.org/10.1074/jbc.M611439200 PMID: 17339319

83. Campos-Bermudez VA, Bologna FP, Andreo CS, Drincovich MF. Functional dissection of Escherichia coli phosphotransacetylase structural domains and analysis of key compounds involved in activity regulation. FEBS J. 2010;277(8):1957–66. https://doi.org/10.1111/j.1742-4658.2010.07617.x PMID: 20236319

84. Oberlies G, Fuchs G, Thauer RK. Acetate thiokinase and the assimilation of acetate in methanobacterium thermoautotrophicum. Arch Microbiol. 1980;128(2):248–52. https://doi.org/10.1007/BF00406167 PMID: 6111300

85. Bologna FP, Campos-Bermudez VA, Saavedra DD, Andreo CS, Drincovich MF. Characterization of Escherichia coli EutD: a phosphotransacetylase of the ethanolamine operon. J Microbiol. 2010;48(5):629–36. https://doi.org/10.1007/s12275-010-0091-0 PMID: 21046341

86. Acetate kinase and phosphotransacetylase. In: Methods in enzymology [Internet]. Academic Press; 2011 [cited 2026 Feb 26]. p. 219–31. Available from: https://www.sciencedirect.com/science/chapter/bookseries/pii/B9780123851123000111

87. Cherkasov A, Hsing M, Zoraghi R, Foster LJ, See RH, Stoynov N, et al. Mapping the protein interaction network in methicillin-resistant Staphylococcus aureus. J Proteome Res. 2011;10(3):1139–50. https://doi.org/10.1021/pr100918u PMID: 21166474

88. Cheung GYC, Wang R, Khan BA, Sturdevant DE, Otto M. Role of the accessory gene regulator agr in community-associated methicillin-resistant Staphylococcus aureus pathogenesis. Infect Immun. 2011;79(5):1927–35. https://doi.org/10.1128/IAI.00046-11 PMID: 21402769

89. Thompson TA, Brown PD. Association between the agr locus and the presence of virulence genes and pathogenesis in Staphylococcus aureus using a Caenorhabditis elegans model. Int J Infect Dis. 2017;54:72–6. https://doi.org/10.1016/j.ijid.2016.11.411 PMID: 27915107

90. Cheung GYC, Bae JS, Otto M. Pathogenicity and virulence of Staphylococcus aureus. Virulence. 2021;12(1):547–69. https://doi.org/10.1080/21505594.2021.1878688 PMID: 33522395

91. Wang B, Zhao A, Novick RP, Muir TW. Activation and inhibition of the receptor histidine kinase AgrC occurs through opposite helical transduction motions. Mol Cell. 2014;53(6):929–40. https://doi.org/10.1016/j.molcel.2014.02.029 PMID: 24656130

92. Wang B, Zhao A, Xie Q, Olinares PD, Chait BT, Novick RP, et al. Functional plasticity of the AgrC receptor histidine kinase required for staphylococcal virulence. Cell Chem Biol. 2017;24(1):76–86. https://doi.org/10.1016/j.chembiol.2016.12.008 PMID: 28065658

93. Brown AJ, Goldsworthy SM, Barnes AA, Eilert MM, Tcheang L, Daniels D, et al. The Orphan G protein-coupled receptors GPR41 and GPR43 are activated by propionate and other short chain carboxylic acids. J Biol Chem. 2003;278(13):11312–9. https://doi.org/10.1074/jbc.M211609200 PMID: 12496283

94. Le Poul E, Loison C, Struyf S, Springael JY, Lannoy V, Decobecq ME, et al. Functional characterization of human receptors for short chain fatty acids and their role in polymorphonuclear cell activation. J Biol Chem. 2003;278(28):25481–9. https://doi.org/10.1074/jbc.M301403200 PMID: 12711604

95. Nakajima A, Nakatani A, Hasegawa S, Irie J, Ozawa K, Tsujimoto G, et al. The short chain fatty acid receptor GPR43 regulates inflammatory signals in adipose tissue M2-type macrophages. PLoS One. 2017;12(7):e0179696. https://doi.org/10.1371/journal.pone.0179696 PMID: 28692672

96. Ang Z, Xiong D, Wu M, Ding JL. FFAR2-FFAR3 receptor heteromerization modulates short-chain fatty acid sensing. FASEB J. 2018;32(1):289–303. https://doi.org/10.1096/fj.201700252RR PMID: 28883043

97. Schlatterer K, Beck C, Schoppmeier U, Peschel A, Kretschmer D. Acetate sensing by GPR43 alarms neutrophils and protects from severe sepsis. Commun Biol. 2021;4(1):928. https://doi.org/10.1038/s42003-021-02427-0 PMID: 34330996

98. Galvão I, Tavares LP, Corrêa RO, Fachi JL, Rocha VM, Rungue M, et al. The metabolic sensor GPR43 receptor plays a role in the control of Klebsiella pneumoniae infection in the lung. Front Immunol. 2018;9:142. https://doi.org/10.3389/fimmu.2018.00142 PMID: 29515566

99. Sencio V, Barthelemy A, Tavares LP, Machado MG, Soulard D, Cuinat C, et al. Gut dysbiosis during influenza contributes to pulmonary pneumococcal superinfection through altered short-chain fatty acid production. Cell Rep. 2020;30(9):2934-2947.e6. https://doi.org/10.1016/j.celrep.2020.02.013 PMID: 32130898

100. Schlatterer K, Peschel A, Kretschmer D. Short-chain fatty acid and FFAR2 activation – A new option for treating infections? Front Cell Infect Microbiol. 2021;11:785833. https://doi.org/10.3389/fcimb.2021.785833 PMID: 34926327

101. Xu W, Bradstreet TR, Zou Z, Hickerson S, Zhou Y, He H, et al. Reprogramming aerobic metabolism mitigates Streptococcus pyogenes tissue damage in a mouse necrotizing skin infection model. Nat Commun. 2025;16(1):2559. https://doi.org/10.1038/s41467-025-57348-x PMID: 40089471

102. Overbeek R, Begley T, Butler RM, Choudhuri JV, Chuang H-Y, Cohoon M, et al. The subsystems approach to genome annotation and its use in the project to annotate 1000 genomes. Nucleic Acids Res. 2005;33(17):5691–702. https://doi.org/10.1093/nar/gki866 PMID: 16214803

103. Overbeek R, Olson R, Pusch GD, Olsen GJ, Davis JJ, Disz T, et al. The SEED and the Rapid Annotation of microbial genomes using Subsystems Technology (RAST). Nucleic Acids Res. 2014;42(Database issue):D206-14. https://doi.org/10.1093/nar/gkt1226 PMID: 24293654

104. Letunic I, Bork P. Interactive Tree of Life (iTOL) v6: recent updates to the phylogenetic tree display and annotation tool. Nucleic Acids Res. 2024;52(W1):W78–82. https://doi.org/10.1093/nar/gkae268 PMID: 38613393

105. Monk IR, Tree JJ, Howden BP, Stinear TP, Foster TJ. Complete bypass of restriction systems for major Staphylococcus aureus lineages. mBio. 2015;6(3):e00308-15. https://doi.org/10.1128/mBio.00308-15 PMID: 26015493

106. Acosta IC, Albers A, Fang L, Serrato G, Teoh WP, Glanville DG, et al. Staphylococcus aureus exploits lipoic acid salvage to combat host oxidative stress. Cell Rep. 2025;44(8):116095. https://doi.org/10.1016/j.celrep.2025.116095 PMID: 40751910

107. Urso A, Monk IR, Cheng Y-T, Predella C, Wong Fok Lung T, Theiller EM, et al. Staphylococcus aureus adapts to exploit collagen-derived proline during chronic infection. Nat Microbiol. 2024;9(10):2506–21. https://doi.org/10.1038/s41564-024-01769-9 PMID: 39134708

108. Huang DW, Sherman BT, Lempicki RA. Systematic and integrative analysis of large gene lists using DAVID bioinformatics resources. Nat Protoc. 2009;4(1):44–57. https://doi.org/10.1038/nprot.2008.211 PMID: 19131956

109. Sherman BT, Hao M, Qiu J, Jiao X, Baseler MW, Lane HC, et al. DAVID: a web server for functional enrichment analysis and functional annotation of gene lists (2021 update). Nucleic Acids Res. 2022;50(W1):W216–21. https://doi.org/10.1093/nar/gkac194 PMID: 35325185

110. Miller J. Assay of β-galactosidase. In: Experiments in molecular genetics. Cold Spring Harbor (NY): Cold Spring Harbor Laboratory; 1972. p. 352–5.

111. Agbavor C, Torres M, Inniss NL, Latimer S, Minasov G, Shuvalova L, et al. Structural analysis of extracellular ATP-independent chaperones of streptococcal species and protein substrate interactions. mSphere. 2025;10(2):e01078-24. https://doi.org/10.1128/msphere.01078-24

112. Scheuermann TH, Padrick SB, Gardner KH, Brautigam CA. On the acquisition and analysis of microscale thermophoresis data. Anal Biochem. 2016;496:79–93. https://doi.org/10.1016/j.ab.2015.12.013 PMID: 26739938
